# Ethnopharmacological Study of Medicinal Plants Used for the Treatment of Cardiovascular Diseases and Their Associated Risk Factors in sub-Saharan Africa

**DOI:** 10.3390/plants11101387

**Published:** 2022-05-23

**Authors:** Johnson Oluwaseun Odukoya, Julianah Olayemi Odukoya, Edwin Mpho Mmutlane, Derek Tantoh Ndinteh

**Affiliations:** 1 Centre for Natural Products Research, Department of Chemical Sciences, University of Johannesburg, Doornfontein, P.O. Box 17011, Johannesburg 2028, South Africa; dndinteh@uj.ac.za; 2 Department of Chemistry, The Federal University of Technology, Akure PMB 704, Ondo State, Nigeria; 3 Department of Biotechnology and Food Technology, Faculty of Science, University of Johannesburg, Doornfontein, P.O. Box 17011, Johannesburg 2028, South Africa; julianahodukoya@gmail.com; 4 Department of Food Science and Technology, Kwara State University, Malete, Ilorin PMB 1530, Kwara State, Nigeria

**Keywords:** diabetes mellitus, ethnobotany, food safety, hyperlipidemia, hypertension, nutraceuticals, obesity, phytochemicals, stroke, traditional medicine

## Abstract

Cardiovascular diseases (CVDs) are the leading cause of global mortality, including deaths arising from non-communicable diseases in sub-Saharan Africa (SSA). Consequently, this study aimed to provide details of medicinal plants (MPs) employed in SSA for the treatment of CVDs and their related risk factors to open new avenues for the discovery of novel drugs. The extensive ethnopharmacological literature survey of these MPs in 41 SSA countries was based on studies from 1982 to 2021. It revealed 1,085 MPs belonging to 218 botanical families, with Fabaceae (9.61%), Asteraceae (6.77%), Apocynaceae (3.93%), Lamiaceae (3.75%), and Rubiaceae (3.66%) being the most represented. Meanwhile, *Allium sativum* L., *Persea americana* Mill., *Moringa oleifera* Lam., *Mangifera indica* L., and *Allium cepa* L. are the five most utilised plant species. The preferred plant parts include the leaves (36%), roots (21%), barks (14%), fruits (7%), and seeds (5%), which are mostly prepared by decoction. Benin, Mauritius, Nigeria, South Africa, and Togo had the highest reported use while most of the investigations were on diabetes and hypertension. Despite the nutraceutical advantages of some of these MPs, their general toxicity potential calls for caution in their human long-term use. Overall, the study established the need for governments of SSA countries to validate the efficacy/safety of these MPs as well as provide affordable, accessible, and improved modern healthcare services.

## 1. Introduction

Cardiovascular diseases (CVDs), a group of disorders involving/affecting the heart and blood vessels, are the chief cause of death in the world [1,2,3,4,5]. According to WHO [6], CVDs claim an estimated 17.9 million lives every year with four out of five CVD deaths resulting from heart attack and stroke. This specialized agency of the United Nations in charge of international public health (WHO) added that one-third of these deaths occurs prematurely in people that are less than 70 years old. Olorunnisola et al. [7] indicated that between 1990 and 2020, death arising from CVDs was projected to increase from 28.9 to 36.3%.

In sub-Saharan Africa (SSA), with 47 countries and a land mass of approximately 24 million km^2^ [8] lying completely or partially south of the Sahara desert [9], CVDs are the most common cause of deaths arising from non-communicable diseases [10] and responsible for most of the mortality of those above 45 years old in Africa [11]. As reported in Amegah [12], SSA was the only geographical region of the world between 1990 and 2013 where an increase in CVD deaths increased. Cardiac diseases have also been identified as a major non-communicable disease among children in SSA [13]. Several authors such as BeLue et al. [9], Monti et al. [14], Amegah [12], van der Sande [15], Yach et al. [16], Tokoudagba et al. [17], Nkoke and Luchuo [18], and Owusu and Acheamfour-Akowuah [19], among others, have attributed the increase in the burden of CVDs and related risk factors in the region to certain agents. These include the epidemiological transition brought about by increasing urbanization, changing lifestyle/dietary patterns, socioeconomic development, and modernization. van der Sande [15] pointed out that age-specific rates of many CVDs are growing faster among adults in SSA than those in industrialised countries.

Generally, CVDs can be defined as “the pathologic process (usually atherosclerosis) affecting the entire arterial circulation, not just the coronary arteries” [20]. They include heart failure (also referred to as congestive heart failure), stroke, coronary artery, coronary heart, cerebrovascular, and rheumatic heart diseases [1,6,21,22], as well as other conditions, with diabetes, hypertension, high cholesterol/hyperlipidemia, obesity (abnormal or excessive fat accumulation), tobacco/cigarettes use, increase in age, sedentary lifestyle, and genetic predisposition, among others, as risk factors [2,3,7,20,23,24]. In SSA countries, the CVDs challenge has given rise to additional pressure on the healthcare systems, which are still struggling to cope with other diseases that affect the region [19].

With respect to CVDs’ treatment in the region, biologically based therapy (such as herbal therapy) is the most employed traditional, complementary, and alternative medicine (TCAM) [25]. According to Bussmann et al. [26], traditional medicine (TM) is a means of treatment in developing countries, whereas Complementary Alternative Medicine (CAM) is common in developed nations. The World Health Organization [27] gave a clear difference between “traditional medicine” (indigenous-based) and “complementary medicine” or “alternative medicine” (not indigenous-based or conventional), and indicated that the terms are used interchangeably in some countries. Meanwhile, herbal medicines involve the use of herbs and herbal materials/preparations/products with plant parts/materials, or their combinations, as active ingredients [27,28].

From the published literature, the use of TM in developing countries can be attributed to (a) accessibility, familiarity, and tradition [29,30], (b) inadequate/limited access to medical service providers or modern health care systems [29,31,32,33], (c) unavailability or high cost of modern medicine [29,30,32], (d) increased awareness of the potential of alternative medicines [32], and (e) perceived safety or comparatively less toxicity to synthetic drugs [30,33,34,35], among others. Generally, in Africa, Nafiu et al. [28] noted that it is arguably because of cultural and economic reasons. Among other factors, Rahmatullah et al. [36] added that people are gradually resorting back to TM, which involves the use of medicinal plants (MPs) as a result of the evolution of multi-drug-resistant microorganisms and the inability of modern medicine to effectively cure some diseases.

As several researchers have documented the use of MPs, i.e., plants containing secondary metabolites with therapeutic benefits [37], for the traditional treatment of CVDs as well as their associated risk factors in many SSA countries, this review article, thus, serves as a collection of these MPs to aid CVD research and the production of novel drugs for these diseases. It would also support (i) the preservation of indigenous knowledge, (ii) conservation and utilization of biological resources [38], (iii) discovery of new treatment for all types of CVDs [1], and (iv) future research works on the efficacy, as well as safety, of these MPs.

## 2. Results and Discussion

### 2.1. Medicinal Plants

Plants have continued to play important roles in supporting human life [39] in different parts of the world, Africa inclusive [33]. Among these are MPs, which contain compounds that have therapeutic properties or exert pharmacological effects on the human body [40]. Generally, MPs naturally synthesize and accumulate secondary metabolites (e.g., alkaloids, sterols, terpenes, flavonoids, saponins, glycosides, and tannins) [40] while their extracts have found applications in pharmaceutical, nutraceutical as well as other chemical industries [41].

**Figure 1:**
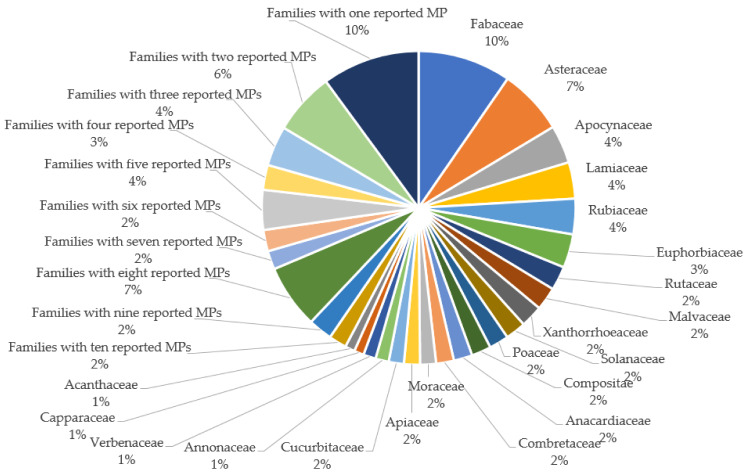
Percentage distribution of families of medicinal plants used in sub-Saharan Africa for cardiovascular diseases treatment.

The ethnobotanical data collection led to the gathering of information on 1085 plant species from 218 botanical families reported in one (Table 1) and two or more SSA countries (Table 2) for CVDs as well as their associated risk factors treatment. The use of different MPs in various parts of SSA for the treatment of these diseases in relation to Kose et al. [42] may be attributed to the different background, belief, and available plants. It is also an indication of the region’s rich traditional knowledge on MPs [38], which may be linked to the difficulty in assessing medical care [43]. The distribution of the studied MPs within botanical families is illustrated in Figure 1, where Fabaceae (9.61%), Asteraceae (6.77%), Apocynaceae (3.93%), Lamiaceae (3.75%), and Rubiaceae (3.66%) are the five most encountered families, which could be connected to their wide range of bioactive compounds [38], availability, and popularity/traditional knowledge of plant species belonging to these families in many parts of SSA. The total number of plants belonging to these five plant families represents 27.72% of the total MPs covered in this study. This indicated that these five botanical families include MPs that are mostly used for the treatment of the diseases of focus in the region.

The current review of the literature also revealed that some of the SSA MPs are used singly or as a mixture of more than one species for the treatment of CVDs and their related risk factors (e.g., see Gbolade [123]). According to Namukobe et al. [176] and Tugume et al. [38], the use of different plant combination for the effective treatment of one disease may be attributed to their synergistic effects. Some of these MPs are also employed in the treatment of other diseases, which, in line with Namukobe et al. [176] and Tugume et al. [38], is because they contain many metabolites, as well as on the basis that the same molecule can be active against different pathogens.

Most Utilised MPs for the Treatment of CVDs in SSA

**Figure 2:**
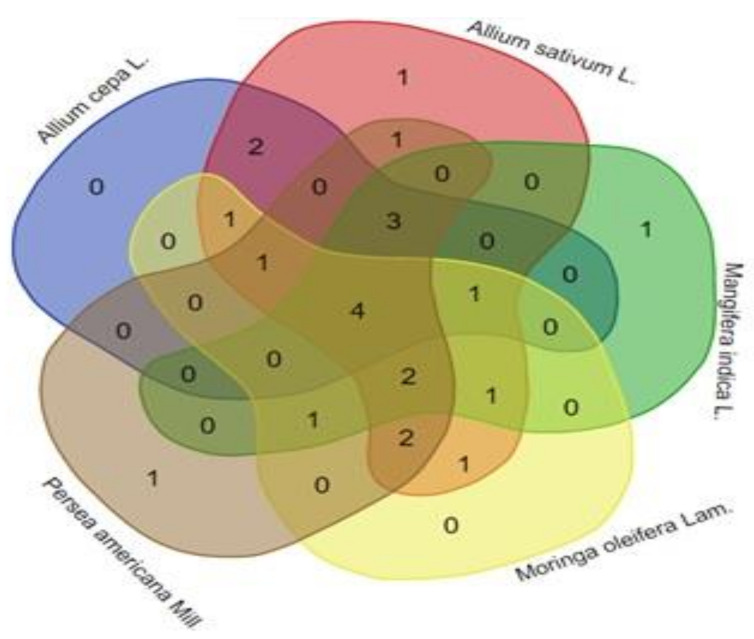
Venn diagram comparing the frequency of use of the five most utilised medicinal plants for cardiovascular diseases treatment in sub-Saharan African countries.

The ethnobotanical survey showed that *Allium sativum* L., *Persea americana* Mill., *Moringa oleifera* Lam., *Mangifera indica* L., and *Allium cepa* L. are the five most utilised MPs in SSA countries (Table 3) for the treatment of CVDs and their related risk factors. In addition, these five plants, as illustrated in Figure 2, have together found applications in four countries in SSA, namely, Benin, Mauritius, Nigeria, and Togo, for the diseases’ treatment.

### 2.2. Plants Parts Used

**Figure 3:**
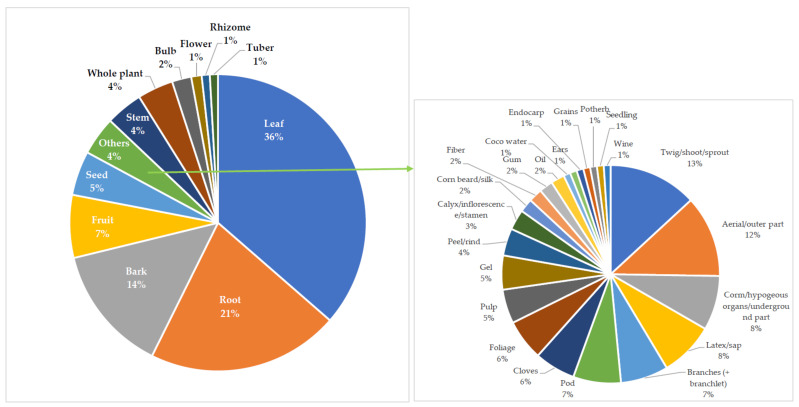
Percentage occurrence of the different plant parts used for the treatment of cardiovascular diseases in sub-Saharan Africa.

Nadembega et al. [54] recorded that in African ethno-medicine, all parts of a plant, and on many occasions, different parts of the same plant, are used for various diseases’ treatment. In the current study, the ethnobotanical survey of MPs used for the treatment of the diseases of focus, as shown in Table 1 and Table 2, identified 44 plants’ parts (major: 11 (whole plant inclusive); minor: 33) with the leaves (36%), roots (21%), barks (14%), fruits (7%), and seeds (5%) being the most frequently used (see Figure 3). This is similar to the research findings of Sabiu et al. [53], Davids et al. [143], and Kpodar et al. [173] as well as Aumeeruddy and Mahomoodally [23] where leaves were the most used plant part. Other researchers, such as Randriamiharisoa et al. [30], Wubetu et al. [80], Yebouk et al. [112], Suleiman [158], Mahomoodally et al. [182], and Rachid et al. [234], also identified MPs’ leaves as the most employed.

The high percentage of usage recorded for leaves, with respect to other plant parts, can be linked to their fast regeneration level [176], potency [143,176], traditional belief to be the strongest part of plant [143], abundance, availability [23,53,173,235], ease of collection in large quantities, and presence as the key photosynthetic organ in plants, which are thought to have accumulated a higher concentration of active ingredients/secondary metabolites [29,38,116,182,236]. These factors contributing to leaves being the most employed plant part in TM, according to Akerreta et al. [237], as well as Chintamunnee and Mahomoodally [116], can be attributed to them being the most exposed to environmental damages/threat. This environmental threat brings about the synthesis/production of active compounds in the leaves—as a means of defence, protection, and survival—with human health benefits. Although other parts of plants also contain compounds of pharmacological importance, their unavailability throughout the year affect their usage [237]. At the same time, excessive use/overcollection of fruits and seeds may negatively affect plant genetic diversity and distribution based on their role in sexual reproduction and dispersal [23,235].

In addition, harvesting of the bark, though easily collected with concentrated bioactive compounds [87], has a negative effect on the protection level of the plant [38]. Generally, the collection of bark, roots, or the whole plant is destructive and may lead to overexploitation/species depletion thereby affecting their sustainability [23,38,87]. This suggests that for plants such as *Azadirachta indica* (neem), *Carica papaya* (pawpaw), and *Persea americana* (avocado), among others, in which more than one plant part is used for treatment (Table 2), their sustainability would be achieved if the harvesting of bark and root is discouraged while that of leaves (being less destructive when taken in small quantities) is encouraged. Notwithstanding, the overharvesting of leaves may also have a negative impact on the overall natural regeneration of the plants and conservation [38,143].

### 2.3. Modes of Preparation

**Figure 4:**
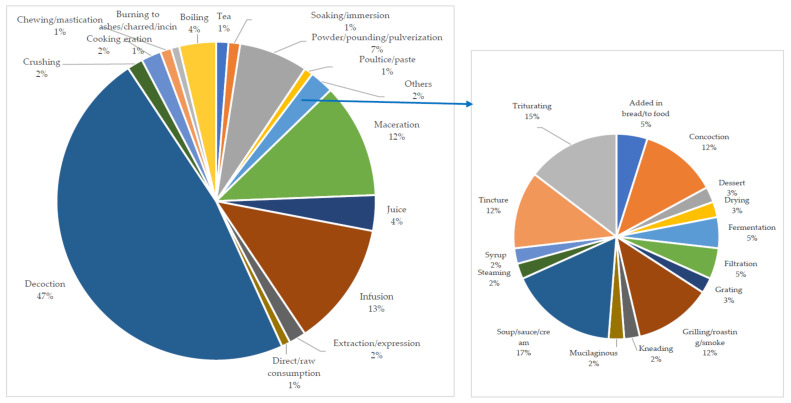
Percentage use of the different modes of preparation of medicinal plants for the treatment of cardiovascular diseases in sub-Saharan Africa.

The review showed that MPs that have found applications in TM are prepared in various forms [237] prior to their use. In this study, decoction was discovered to be the most employed mode of preparation (47%) of MPs used for the treatment of the diseases of focus in SSA (Figure 4). Similar results for the most used method of preparation was reported by Tjeck et al. [87], Gbolade [123], Kpodar et al. [173], as well as Aumeeruddy and Mahomoodally [23]. According to the latter authors, i.e., Aumeeruddy and Mahomoodally [23], the fast nature of decoction, when compared to the application of other methods (e.g., maceration) of which the extraction process takes a longer period of time, may be responsible for it being the most preferred method of preparation. Notwithstanding, Tugume et al. [38] noted that decoctions as well as cold extracts have the disadvantage of a short shelf life, which leads to the continuous harvesting of these MPs and may contribute to over-exploitation. After the preparation of a decoction or maceration, Nadembega et al. [54] indicated that the remaining solid part of the MP is sometimes converted to a powder via grinding or charring. As noted by these authors, the liquid used in extinguishing the flames determines the therapeutic application of the obtained charred material.

**Figure 5:**
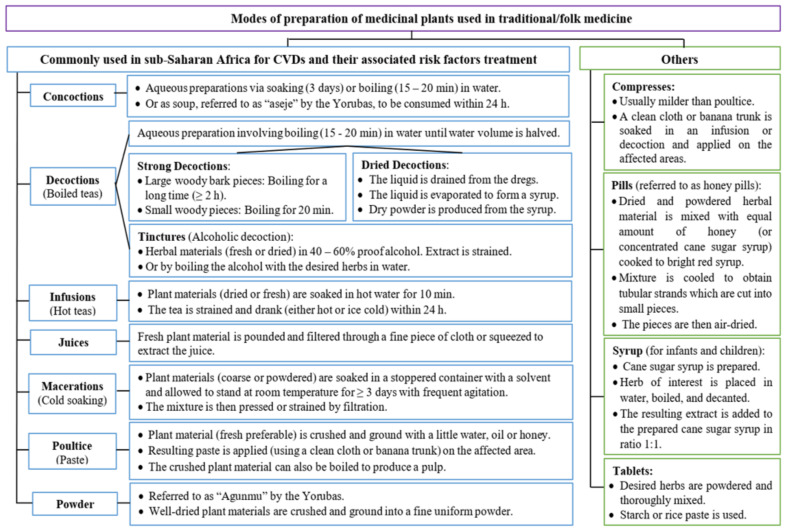
Connection between the different modes of preparing medicinal plants used for the treatment of cardiovascular diseases in sub-Saharan Africa. Adapted from: Nafiu et al. [28] and Azwanida [238].

Furthermore, the ease at which powder is prepared encouraged its use, which is less prone to contamination when compared with juices, infusions, and decoctions [112]. Boiling is reported to be effective for extraction and aids the preservation of the herbal remedy for a longer duration than when cold extraction is used [38]. As pointed out in the work of Ong et al. [235], as well as that of Aumeeruddy and Mahommodally [23], heat application in herbal preparations also promotes the availability of bioactive compounds by accelerating biological reactions. According to Wubetu et al. [80], the use of these simple approaches by traditional medical practitioners without further processing can be linked to their lack of processing equipment and poor literacy level. Figure 5 shows the connection between some of these different modes of preparation used in TM for the treatment of CVDs and their associated risk factors.

### 2.4. Diseases Treated

**Figure 6:**
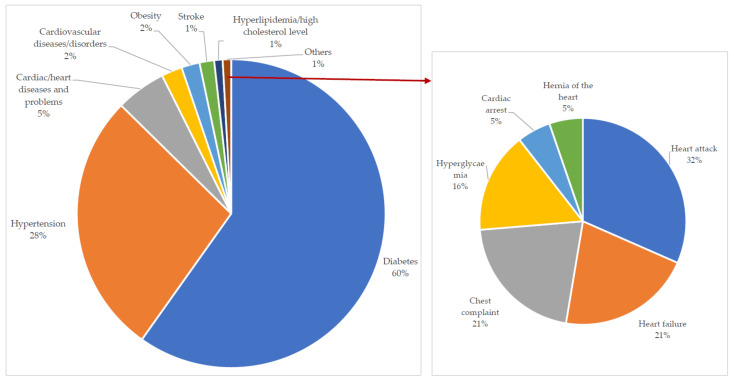
Percentage of cardiovascular diseases and their related risk factors treated with medicinal plants in sub-Saharan Africa.

The investigation revealed that among the identified CVDs and associated risk factors, most applications of MPs in SSA are for diabetes (60%), normally assumed as diabetes mellitus [53], as well as hypertension (28%) treatment (Figure 6). Kpodar et al. [173] noted that type II diabetes constitutes approximately 90% of all diabetes while Davids et al. [143] reported that there is considerable overlap between this disease (type II diabetes mellitus) and hypertension in aetiology as well as disease mechanisms.

The research outcome suggests awareness of these diseases (diabetes and hypertension) in the region, and in support of the literature, e.g., Salihu Shinkafi et al. [128], the reliance on TCAM for their treatment. On the other hand, it indicated a lack of in-depth knowledge of other diseases that make up the group of heart and blood vessel disorders, including other risk factors. This, therefore, pinpoints the need for improved access to screening and detection [12], modern health services/medicine, as well as enlightenment to reduce the growing burden of CVDs in SSA.

### 2.5. Use in Different SSA Countries

**Figure 7:**
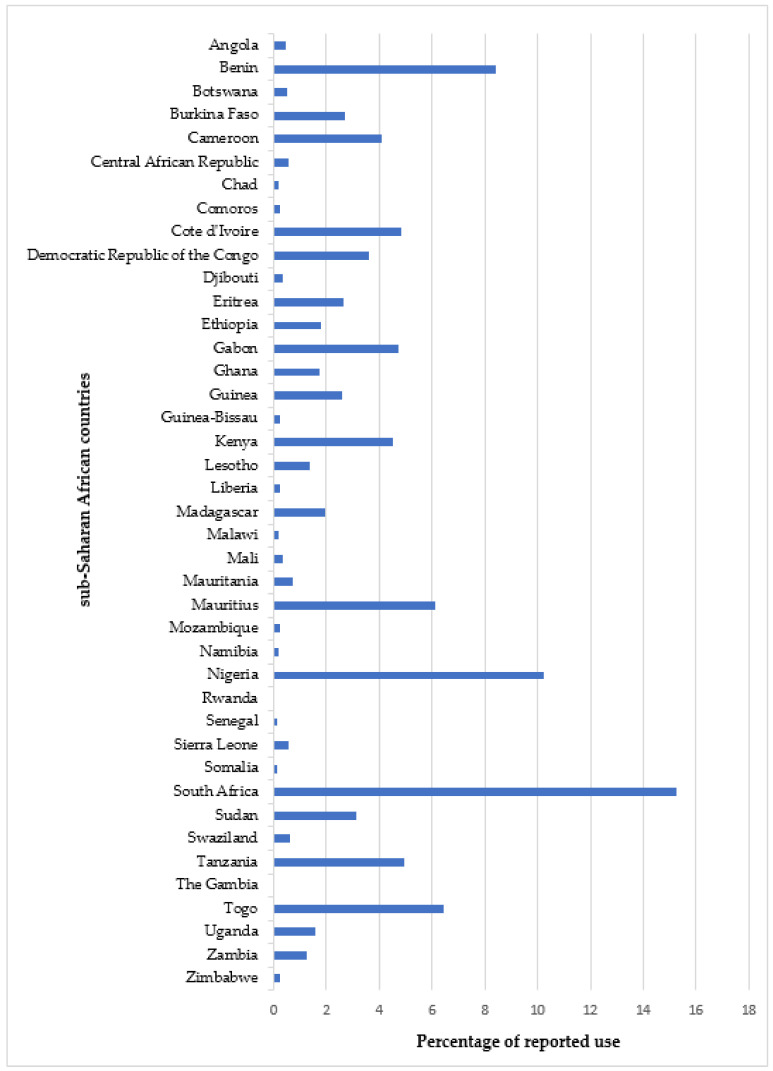
Percentage of reported use of medicinal plants for cardiovascular diseases treatment in each of the sub-Saharan African countries.

Figure 7 reveals the reported use of MPs for the treatment of the diseases of focus in 41 SSA countries. The results of the study indicated that in SSA, the scientifically reported use of MPs for these diseases was mostly in South Africa (15.25%), Nigeria (10.24%), Benin (8.43%), Togo (6.44%), and Mauritius (6.11%). Out of the 41 SSA countries covered in this review, those with the least reported use of MPs for the treatment of these diseases are Chad, Comoros, The Gambia, Guinea-Bissau, Liberia, Malawi, Mozambique, Namibia, Rwanda, Senegal, Somalia, and Zimbabwe (Figure 7). However, there are countries in SSA, e.g., Burundi, Niger, Sao Tome, and Principe, among few others, in which the use of MPs for CVD treatment is yet to gain scientific recognition.

The research findings, in a way, reflect the burden of CVDs and their related risk factors in some SSA countries. For instance, Nigeria (2.7 million people) and South Africa (4.6 million people) are among the top five countries (with the other three being the Democratic Republic of the Congo (1.8 million people), Ethiopia (1.7 million people), and the United Republic of Tanzania (1 million people)) reported by the International Diabetes Federation (IDF) with the highest number of people with diabetes (20–79 years) in Africa as of 2019 [239]. As mentioned earlier (see Section 2.4), the study outcome also indicates a dependence on TCAM for CVD treatment and the need for improved modern healthcare services in these countries.

### 2.6. Phytochemicals Distribution in the MPs

Okwu [240] noted that the medicinal potentials of plants may be assumed to be linked primarily to their phytochemicals, vitamins, and minerals contents. Many of these phytochemicals or secondary metabolites are useful for drug development [236], and in plants, may act individually, additively, or synergistically to improve health [28]. The three key groups of these secondary metabolites vis-à-vis their biosynthetic pathway have been identified as nitrogen-containing compounds (alkaloids, cyanogenic glycosides, and glucosinolates), phenolic compounds (phenolic acids, flavonoids, and phenylpropanoids), and terpenes/terpenoids (isoprenoids) [236,241,242].

Gan et al. [243] reported that phenolic compounds are the key antioxidant ingredients in several MPs while Sharifi-Rad et al. [24] identified polyphenols as one of the chief groups of cardioprotective agents in herbs. Some of these polyphenols, such as flavonols, also help in reducing CVDs risk factors [244]. The level of flavonols in free-standing leaves is, however, most times significantly higher than that in other parts of the same plant, except in *A. cepa* [245].

In general, polyphenols aid cardiovascular health by inhibiting platelet aggregation, modulating inflammation and lipid metabolism, limiting low-density lipoprotein (LDL) oxidation, and improving endothelial function and antioxidant status, among other functions [24,246]. Specifically, Odukoya et al. [4] and Petrovski et al. [247] identified catechin, quercetin, and resveratrol as some of the bioactive compounds that have shown the ability to reduce CVD risk and assist in cardioprotection. Table 4 highlights the key bioactive compounds in the five most preferred MPs, as reported in this study, for the treatment of CVDs and their associated risk factors in SSA. With respect to Nafiu et al. [28], although different pharmacological drugs have been produced, several phytochemicals present in MPs work together to provide a combined effect that is stronger than when individual constituents are used.

### 2.7. MPs with Nutraceutical Advantages

TM includes the use of medicinal herbs and food plants [182], while Belayneh et al. [78] referred to MPs with edible parts as nutraceutical plant species. The same part of some of these plants, e.g., *Carissa spinarum* and *Senna occidentalis*, have been used as food and for medicinal purposes [44]. According to Urso et al. [44], plants with possible pharma-food or nutraceutical properties, considered as “pharma-foods” or “folk nutraceuticals”, have closely related food and medicinal applications. Nevertheless, Göhre et al. [43] pointed out that not all these plants are included in the diet under normal circumstances, as some of them, referred to as famine foods, are only consumed when there is a food shortage. Some of the edible parts of these plants, such as the fruits and tubers (underground organ), contribute to the local diet and livelihood of a number of local communities in SSA [43,44]. Göhre et al. [43] added that, in terms of nutrition, fruits are the most used plant part.

The current study reported the direct consumption of the fruit of Balanites aegyptiaca, Citrus maxima, Phyllanthus emblica, Psidium guajava, Strychnos spinosa and Syzygium cumini; the seed and fruit of Kigelia africana; the seed of Vitis vinifera; the bulb of Allium sativum as well as the tubers of Daucus carota and Dioscorea bulbifera for the treatment of CVDs in certain SSA countries. The leaves of other MPs, e.g., Gnetum africanum and Vernonia amygdalina, have also been used for soup preparation and CVDs treatment in the region. Several other MPs, including Anacardium occidentale, Citrus limon and Tamarindus indica [78,94], have reported food and medicinal plant applications. Some of them are available in local and country markets [78,94]. Notwithstanding, unless properly prepared, the roots or tubers of some of these plants, for instance the tubers of Dioscorea species and cassava, Manihot esculenta, with respect to Urso et al. [44], affect human health negatively.

### 2.8. Reported Adverse Effects/Toxicity

Although the use of TM/MPs/herbs are perceived to be safe or potentially less toxic when compared to conventional treatment [30,34,35,53,128], several authors, including Mounanga et al. [33], Bekoe et al. [35], Subramanian et al. [37], and Brima [268], have indicated that they can also have negative effects on human health. According to Kharchoufa et al. [269], the use of MPs in developing countries threaten public health as they have been linked to carcinogenic, teratogenic, and life-threatening effects, and even death. Mounanga et al. [33] revealed that most of the approximately 1.5 million investigated plants contain toxic substances. These toxic substances, as noted by Yumnamcha et al. [270] and Chahardehi et al. [271], are synthesized/produced by plants as a means of defence against diseases/infections, insects, and other organisms. Examples of these are cytotoxic and genotoxic substances [272]. MPs can also be a source of exposure to the risk of toxic elements [268].

Generally, the toxicity of plants/MPs, among other factors, depends on the nature/strength of their secondary metabolites, quantity consumed, plant part, consumer’s body chemistry, climatic/soil conditions, the genetic differences within the species, as well as their reaction with other herbs, drugs, contaminants, and adulterants [33,234,268,273]. This necessitates the use of MPs with caution [33], particularly in situations where long-term application in humans is practised.

Kharchoufa et al. [269] identified some of the compounds considered toxic or that presented a level of toxicity to majorly belong to five groups of compounds, namely, alkaloids, glucosides, terpenoids, protides, and phenolics. Meanwhile, Yumnamcha et al. [270] pinpointed alkaloids, cardiac glycosides, phorbol esters, lectins, and cynogenic glycosides as some of the major bioactive compounds responsible for the toxic effects of plants. For instance, phorbol esters, occurring naturally in many plants of the family Euphorbiaceae and Thymelaeaceae, are the tetracyclic diterpenoids widely known for their tumour-promoting ability [274]. With respect to the current review, aristolochic acid, one of the active constituents in Aristolochia species, has been linked to carcinogenic, genotoxic, and kidney-damaging effects [37], while, among other MPs, the toxicity of *Citrullus colocynthis*, *Datura stramonium*, and *Ricinus communis* has been indicated by traditional herbal healers [191].

## 3. Methodology

### 3.1. Source of Data and Collection Methods

An extensive literature retrieval, principally from published scientific journals, was used to obtain the required ethnopharmacological information. This was based on studies from 1982 to 2021 with the application of single or combinations of keywords such as medicinal plants, cardiovascular diseases and treatment, diabetes, hypertension, obesity, stroke, and the different names of SSA countries. Major scientific electronic databases [PubMed (National Library of Medicine), Science Direct, Web of Science, Tailor & Francis Online, Wiley Online Library, Google Scholar, and Google], as well as other internet sources, were consulted. To verify the scientific names and families of the identified MPs, other databases such as The Plant List and The World Flora Online [http://www.worldfloraonline.org/ (accessed on 2 February 2022)] were used.

Based on the goal/theme of the review, information gathered from the literature survey, which include the name of the MPs (botanical/scientific, English/common, and local names), family, plant part used, modes of usage/preparation, therapeutic use, and the SSA countries involved, were analysed, as well as grouped accordingly.

### 3.2. Data Analysis

Descriptive statistical analysis of the ethnopharmacological data obtained was achieved using Microsoft Excel software (Microsoft corporation, Redmond, Washington, USA) while a readily available bioinformatics web tool [275] was used to generate a Venn diagram where appropriate.

## 4. Conclusions and Future Perspectives

To aid the research on CVDs and production of novel drugs, this comprehensive ethnopharmacological review provides the necessary details of MPs used for the treatment of CVDs and their associated risk factors in SSA. The study revealed that SSA has a rich history in the use of MPs for the treatment of the diseases of focus and a wealth of knowledge in TM, which some individuals in the region rely on for their health needs. The results showed that there are five dominant botanical families of MPs that have been used in SSA for CVD treatment, with some of the MPs having nutraceutical applications because of their edible parts. *A. sativum* is the most employed MP, which indicates its obtainability in many parts of the region and suggests the presence of potent bioactive compounds in the plant against the development of these diseases.

The ease of collection of MPs’ leaves contributing to their high percentage use shows that the availability of effective and affordable drugs for the treatment of these diseases in the region would assist in encouraging the use of modern medicine. This will also safeguard consumers against the unexpected risk of certain toxic compounds in these MPs and the possible negative effects of overdoses arising from unregulated use. The reliance on decoction as a mode of preparation of these MPs (despite its inherent short shelf-life disadvantage), as well as the major focus on diabetes and hypertension, call for enlightenment, scientific engagements, and urgent government intervention to reduce the number of people suffering from CVDs in the region.

Most importantly, the investigation points out the need for governments of SSA countries such as Nigeria and South Africa, with the high percentage use of MPs reported for these diseases, to provide affordable/accessible high-quality modern healthcare services for the populace. Validation of the claimed medicinal/pharmacological potentials and safety assessment of MPs used in the region for CVDs treatment are also encouraged.

## Figures and Tables

**Table 1:** Medicinal plants used for the treatment of cardiovascular diseases and their associated risk factors in one sub-Saharan African country.

Botanical Name	Family	English/ Common Name	Local Name	Plants’ Parts Used	Mode of Usage/ Preparation	Diseases Treated	Country	References
*Brillantaisia owariensis* P.Beauv.	Acanthaceae			Leaf	Decoction	Cardiac disease, Hypertension	Angola	[43]
*Neoboutonia melleri* (Müll.Arg.) Prain	Euphorbiaceae			Root	Decoction	Diabetes		
*Aloe littoralis* Baker	Xanthorrhoeaceae		Endombo, Otchindombo	Hypogeous organs		Diabetes		[44]
*Dicoma* sp.	Asteraceae		Kaundu			Heart troubles		
*Vernonia hymenolepis* Vatke	Compositae					Hypertension		[45]
*Hemizygia bracteosa* (Benth.) Briq.	Lamiaceae					Cardiovascular diseases, Diabetes	Benin	[46]
*Acmella uliginosa* (Sw.) Cass.	Asteraceae		Awerekpe, Welekpekpe	Whole plant		Diabetes		[47]
*Allium ascalonicum* L.	Amaryllidaceae		Mansa elewe, Ayoman winiwini	Bulb	Maceration, Decoction, Powder			
*Borassus aethiopum* Mart.	Arecaceae		Egui agban, Agontin	Seedling	Powder			
*Burkea africana* Hook.	Fabaceae		Atakpa, Ajasi kake	Root, Bark	Decoction, Maceration			
*Combretum collinum* Fresen.	Combretaceae		Irinkoya, Yahoui	Leaf	Decoction			
*Commelina erecta* L.	Commelinaceae		Olirekou, Hanwin hanwin	Root				
*Crateva adansonii* DC.	Capparaceae		Agni-wewe, Onton zunzin	Leaf	Decoction			
*Crinum zeylanicum* (L.)	Amaryllidaceae		Adodje, Soulou	Whole plant, Bulb				
*Crotalaria retusa* L.	Fabaceae		Okounkrounmanro, Aza	Root	Maceration			
*Croton gratissimus* Bureh.	Euphorbiaceae		Adjekofole ile, Jelele	Leaf/Root	Decoction			
*Cussonia arborea*	Araliaceae		Edigo, Toflo-gotoun	Root	Maceration			
*Cymbopogon giganteus* (Hochst.)	Poaceae		Igbakpo, Gbezin	Leaf	Decoction			
*Dioscorea cayenensis* Lam.	Dioscoreaceae		Kokoro, Kokolo	Tuber				
*Eriosema glomeratum* (Guill. & Perr.) Hook.f.	Fabaceae		Kolo-koriko	Leaf	Maceration			
*Erythrina senegalensis* DC.			Ochiiche, Kpaklessi	Root, Bark of root	Decoction, Maceration/Powder			
*Eugenia aromatica* (L.) Baill.	Myrtaceae		Atikin gbadota	Fruit	Decoction			
*Euphorbia hyssopifolia* L.	Euphorbiaceae		Nonsinwe	Leaf				
*Ficus sycomorus* L.	Moraceae		Ofo, Votin	Sap	Decoction			
*Ficus umbellate* Vahl			Ore, Vounvountin	Leaf, Root				
*Flacourtia indica*	Flacourtiaceae		Jogboro, Gbohounkaje	Branch				
*Gardenia erubescens*	Rubiaceae		Kankanrambo, Dakpla	Root	Infusion			
*Gladiolus dalenii* van Geel	Iridaceae		Osheko, Baka	Bulb	Decoction, Powder			
*Gossypium arboretum* L.	Malvaceae		Owou-akeche, Avokanfoun cheke	Leaf	Decoction			
*Hyptis pectinate* (L.) Poit.	Lamiaceae		Kobojoujou, Houeflou	Root	Maceration			
*Lannea barteri* (Oliv.)	Anacardiaceae		Akou, Zonzon	Bark	Decoction			
*Merremia quinquefolia* (L.)	Convolvulaceae		Alovi-aton	Leaf	Powder			
*Mitracarpus villosus* (Sw.) DC.	Rubiceae		Alekou	Leaf	Powder			
*Monodora myristica* (Gaertn.)	Annonaceae		Ariwo, Sassalikoun	Seed, Bulb	Decoction, Maceration, Powder, Maceration			
*Morelia senegalensis*	Rubiaceae		Agnidja, Aviwin	Root	Maceration			
*Olax subscorpioidea* Oliv.	Olacaceae		Egui miitin, Miitin	Root	Maceration			
*Omphalogonus calophyllus* Baill.	Asclepiadaceae		Ogbo doundoun					
*Parinari curatellifolia* Planch.	Chrysobalanaceae		Idifoun, Awetoun	Root/Bark	Decoction, Powder			
*Paullinia pinnata* L.	Sapindaceae		Akpa, Adakloman	Leaf				
*Pavetta crassipes*	Rubiaceae		Etira, Dakplassou		Decoction			
*Pennisetum americanum* (L.)	Poaceae		Iwasse, Likoun	Root/Seed	Decoction, Powder			
*Phaseolus lunatus* L.	Fabaceae		Ibe, Akpakoun	Seed	Powder, Maceration			
*Raphionacme brownii*	Asclepiadaceae		-	Tuber	Maceration			
*Rhynchosia pycnostachya* (DC.)	Fabaceae		-	Root	Decoction			
*Rourea coccinea*	Connaraceae		Ameje, Ganganlise		Maceration			
*Saba comorensis* (Boj.)	Apocynaceae		-		Decoction			
*Sansevieria liberia* hort.	Dracaenaceae		Akpognan	Leaf/Fruit/Root				
*Schrankia leptocarpa* DC.	Fabaceae		Arobokou, Vehoun	Leaf	Decoction			
*Secamone afzelii* (Schult.)	Asclepiadaceae		Abere-rewan, Anonsiman					
*Sesamum radiatum*	Pedaliaceae		Agbo	Leaf	Triturating			
*Vangueriella spinose*	Rubiaceae			Root	Decoction			
*Zanthoxylum zanthoxyloides* (Lam.)	Rutaceae		Ata, Hetin	Root/Bark	Maceration			
*Vernonia cinerea* (L.) Less.	Asteraceae		Ewe jedi jedi, Houssikousse	Leaf, Stem	Powder, Decoction	Diabetes		[47,48]
*Polygonum senegalensis*	Polygonaceae					Diabetes, Hypertension		[49]
*Cassia siamea*	Fabaceae			Root	Decoction	Hypertension		[50]
*Synsepalum dulcificum* (Schumach. & Thonn.) Daniell	Sapotaceae	Miracle plant		Leaf		Diabetes		[51]
*Aloe zebrina* Baker	Xanthorrhoeaceae	Variegated aloe	Kgophane	Leaf	Juice	Diabetes	Botswana	[52]
*Erythrococca trichogyne* (Mull. Arg.)	Euphorbiaceae	Twin redberry	Mononyane	Root	Powder	Diabetes		
*Pterodiscus ngamicus*	Pedaliaceae		Pelo-ya-khutsana	Bulb	Boiling	Heart problems		
*Myrothamnus flabellifolius* (Sond.)	Myrothamnaceae	Resurrection plant	Moswaarula, Rufandichimuka	Shoot, Leaf, Twig	Powder, Tea, Boiling	Diabetes, Stroke		[52,53]
*Acacia dudgeoni*	Mimosaceae		Gompagnalega	Branch	Decoction	Heart disorders	Burkina Faso	[54]
*Acacia pennata* Willd.	Mimosaceae		Kanre		Charred	Heart disorders, Stroke		
*Cadaba farinosa* Fosrk.	Capparaceae		Kinsga	Leaf	Decoction	Diabetes		
*Combretum adenogonium* Stend ex. A. Rich.	Combretaceae		Kuilinga	Leaf	Powder	Hypertension		
*Cymbopogon proximus* Staff.	Poaceae		Soom-piiga	Root	Maceration	Hypertension, Heart disorders		
*Entada Africana* Guill. & Perr.	Mimosaceae		Saparga	Leaf	Decoction	Heart disorders		
*Gardenia aqualla* Stapf & Hutch	Rubiaceae		Namzuuding palaaga	Stem bark				
*Gardenia sokotensis* Hutch.			Tangrambrezugga		Powder	Heart disorders, Hypertension		
*Gossypium* sp.	Malvaceae		Lamtiiga	Fruit	Charred	Heart disorders		
*Hibiscus cannabinus* L.	Malvaceae		Beerga	Ears	Maceration			
*Hygrophila auricolata* Heine	Acanthaceae		Kiaga	Whole plant		Heart disorder		
*Lantana rhodesiensis* Moldenke	Verbenaceae		Niuli sibi	Root	Smoke	Hypertension		
*Ocimum canum* Sims.	Lamiaceae		Yusinyuudu	Whole plant	Decoction	Heart diseases		
*Sesbania pachycarpa* DC.	Fabaceae		More	Fruit	Decoction	Hypertension		
*Sorghum guineense* Stapt.	Poaceae		Ki	Seed	Paste	Obesity		
*Vigna subterranean* (L.) Verdc	Fabaceae		Summinga	Fruit	Maceration	Heart disorders		
*Indigofera tinctoria* (L.)	Fabaceae		Garga	Whole plant	Decoction	Diabetes		[54,55]
*Ambligonocarpus andongensis*	Mimosaceae		Kassi (Mboum), Yake (Fd)	Seeds	Boiling	Hypertension	Cameroon	[56]
*Cleome ciliata*	Capparaceae		Mbango (Douala)	Leafy stem	Decoction	Heart ache		
*Cynodon dactylon*	Poaceae	Bahamas grass	Semesm (Bakossi)	Leaf, Bark, Roots		Hypertension		
*Drynaria cordata*	Polypodiaceae	Chick weed	Echimekede (Bakossi)	Leaf, Root		Diabetes		
*Rauvolfia macrophylla*	Apocynaceae		Kanja (Bakweri)	Bark, Roots	Decoction	Heart ache		
*Bridelia ndellensis* Beille	Euphorbiaceae					Diabetes		[57]
*Bersama engleriana* Gurke	Melianthaceae			Leaf, Stem, Bark, Roots				
*Vitex cienkowskii*	Verbenaceae			Stem bark		Cardiovascular disease		[32]
*Terminalia superba*	Combretaceae			Bark, Stem bark		Diabetes, Hypertension		[32,58]
*Oryza sativa* L.	Poaceae					Hyperglycaemia		[59]
*Citrus grandis* (L.)	Rutaceae		Limi gnamba	Fruit	Juice	Hypertension		[60]
*Milletia sanagana*	Fabaceae		Bolete wanjo	Roots	Maceration			
*Palisota hirsuta*	Commenlinaceae		Ekok	Leaf	Decoction			
*Playcerium stemaria*	Polypodiaceae		Kefafarna, Agbeuth	Whole plant	Incineration	Hypertension, Cardiac palpitations		
*Pterygota* sp.	Sterculiaceae		Wuoho	Bark	Maceration	Hypertension		
*Sloetiopsis usambarensis* Engl.	Moraceae		Otomo landjana	Bark	Decoction			
*Aframomum pruinosum* Gagn.	Zingiberaceae		Keshunedieme	Seeds	Maceration	Cardiac palpitation		[61]
*Crassocephalum crepidiodes* (Benth.)	Asteraceae		Ajujuaphe	Leaf	Infusion	Hypertension		
*Galium asparine* Linn.	Rubiaceae		Njekuba	Whole plant		Obesity		
*Laportea ovalifolia*	Urticaceae		Nantuateneleune			Diabetes		
*Millettia barteri*	Fabaceae			Bark root		Cardiac pain		[62]
*Vepris heterophylla*	Rutaceae			Leaf		Cardiovascular disorder, Hypertension		[63]
*Vepris louisii*	Rutaceae					Hypertension		
*Protea madiannensis*	Proteaceae		Gbogbo (Banda), Zeradope (Gbaya)	Roots	Boiling, Decoction	Hypertension	Central African Republic	[64]
*Capsicum annum* L.	Solonaceae	Pepper (English)	Ndongo (Sango)	Leaf	Boiling, Decoction			
*Tambourissa comorensis* Lorence	Monimiaceae					Diabetes, Hypertension	Comoros	[65]
*Thomandersia hensii*	Acanthaceae		Ikoka, Liowa	Leaf	Decoction	Diabetes	Democratic Republic of Congo (DR Congo)	[66]
*Crinium ornatum*	Amaryllidaceae				Decoction			
*Brassica juncea*	Brassicaceae		Ndunda		Decoction			
*Ipomoea mauritiana*	Convolvulaceae			Tubercule				
*Tetracera poggei*	Dilleniaceae			Leaf				
*Penianthus longifolius*	Menispermaceae			Bark	Maceration			
*Panda oleosa*	Pandaceae		Okali	Bark	Decoction			
*Solanum gilo*	Solanaceae		Nyanya	Root	Decoction			
*Vinca minor*	Apocynaceae		Vinka nyeupe	Leaf and roots	Decoction	Diabetes		[67]
*Artemisia annua*	Asteraceae			Leafy stem				
*Basella alba*	Basellaceae		Nderema, Ndelama	Leaf				
*Albizia grandibracteata*	Fabaceae		Mushebeye, Kahunda, Mushebele	Bark	Decoction	Diabetes		
*Caesalpinia decapetala*			Lurhe	Leaf	Infusion			
*Stachytarpheta indica*	Verbenaceae		“Insulin”	Leafy stem	Decoction			
*Aloe* sp.	Xanthrorrhoeaceae		Kizimia muliro	Aerial part	Expression			
*Annona arenaria*	Annonaceae		Kikolo, Bomengo na esobe	Root	Decoction	Diabetes		[68]
*Rauwolfia obscura*	Apocynaceae		Mudisi	Leaf	Decoction			
*Albizia adianthifolia*	Fabaceae		Kasikeaze, Kapeta nzovu	Leaf, Stem				[69]
*Azanza garckeana*	Malvaceae		Muti ya makamashi	Leaf				
*Gladiolus klattianus*	Iridaceae		Kitala, Kitokatoka	Bulb				
*Vitex madiensis*	Verbenaceae		Mufutu, Mufute Kinka	Leaf, Root				
*Agelanthus dodonefoliusi*	Loranthaceae			Leaf	Decoction	Diabetes	Cote d’Ivoire	[70]
*Albizia lebbeck*	Mimosaceae			Root	Decoction			
*Annona squamosa*	Annonaceae			Leaf	Decoction			
*Argemone mexicana*	Papaveraceae			Root	Decoction			
*Asystasia calycina*	Acanthaceae			Leaf	Decoction			
*Bidens engleri*	Asteraceae			Whole plant	Decoction			
*Chrozophora senegalensis*	Euphorbiaceae			Whole plant	Decoction			
*Chrysophyllum cainito*	Sapotaceae			Stem bark, Leaf	Maceration			
*Clerodendrum inerme*	Verbenaceae			Leaf	Decoction			
*Cnestis ferruginea*	Connaraceae			Leaf, Root	Decoction			
*Drimia glaucescens*	Liliaceae			Whole plant	Decoction			
*Eclipta prostrata* (L) L.	Compositae							
*Ficus glumosa* Delile	Moraceae			Leaf	Decoction			
*Jatroha gossypiifolia*	Euphorbiacea e			Leaf and roots	Trituration			
*Macrosphyra longistyla* (DC.) Hiern	Rubiaceae			Leaf	Decoction			
*Moghania faginea* (Guill. and Perr.) Kuntze	Fabaceae			Leaf	Decoction			
*Ouratea affinis* (Hook. f.) Engl.	Ochnaceae			Leaf	Decoction			
*Sida cordifolia*	Malvaceae			Stems with Leaf and roots	Decoction			
*Stachytarpheta* *jamaicensis* (L.) Vahl.	Verbenaceae			Leaf and axis flowering	Decoction, Infusion			
*Tecoma stans* (L.) Kunth	Bignoniaceae			Leaf	Decoction			
*Terminalia mantaly*	Combretaceae			Stems with Leaf	Decoction			
*Vepris verdoorniana*	Rutaceae			Root bark	Decoction	Hypertension		[63]
*Balanites rotundifolia*	Balanitaceae		Alayto	Leaf	Soaking	Diabetes	Djibouti	[71]
*Buxus hildebrandtii*	Buxaceae		Gaydarto	Leaf	Soaking			
*Lavandula coronopifolia*	Lamiaceae		Dananwada	Leaf	Soaking			
*Melia azedarach*	Meliaceae		Dat caxa	Whole plant	Soaking, Crushing			
*Nepeta azurea*	Lamiaceae		Simitri	Leaf	Soaking, Crushing			
*Anenthum graveolens* Linn	Apiaceae	Dill	Shilan-maedo	Leaf	Tea	Diabetes	Eritrea	[72]
*Cichorium endivia* L.	Asteraceae	Succory	Shikoria	Leaf	Cooking			
*Clutia lanceolate*	Euphorbiaceae	Cerra Cipapau Apple	Tish-belalito	Leaf	Extract			
*Ferula communis* L.	Apiaceae	Giant fennel	Diog	Seed, Leaf	Decoction			
*Psiada panctulata*	Asteraceae		Tsehaiferhet	Leaf, Root	Decoction			
*Trachyspermum ammi*	Apiaceae	Bishop’s Weed	Kamun/Tsakida	Seed	Powder			
*Withania somnifera* (L.)	Solanaceae	Winter Cherry	Agol	Root, Leaf	Root Immersion, Leaf’ juice			
*Zizyphus spina-christi* (L.)	Rhamnaceae	Christ’s Thorn Jujube	Gaba	Leaf	Infusion			
*Aloe camperi* Schweinfurth	Aloaceae	Aloe	Sandai-ere	Leaf, Latex	Extract			[72,73]
*Brassica nigra*	Brassicaceae	Black Mustard	Adri	Seed	Decoction			
*Lepidium sativum* L.	Brassicaceae	Graden Cress	Shinfae	Seed	Extract			
*Meriandra dianthera*	Lamiaceae		Nehiba/Mezeguf/Nehba	Leaf	Extract, Drying, Crushing, Boiling	Diabetes, Hypertension		[72,74]
*Otostegia integrifolia* Benth.	Lamiaceae		Ch’endog/Chendog	Leaf, Bark	Extract, Crushing			
*Acacia senegalensis*	Fabaceae		Tseada-qenteb	Bark	Chewing	Diabetes		[74]
*Aloe elegance*	Aloaceae		Eere	Latex				
*Hypoestes forskaolii*	Acanthaceae		Debe-awald	Leaf	Crushing, Boiling			
*Calpurnia aurea* (Aiton) Benth.	Fabaceae		Cheka, Digita, Digitta	Leaf, Seed	Decoction	Diabetes, Hypertension	Ethiopia	[75,76,77]
*Lens culinaris* Medik.	Fabaceae		Misir	Seed		Diabetes		[76]
*Premna schimperi* Engl.	Lamiaceae		Urgessa	Leaf		Hypertension		
*Indigofera amorphoides* Jaub. & Spach	Fabaceae		Muka Adi, Jeere	All parts	Decoction, Infusion	Heart disease		[78,79]
*Acalypha fruiticosa*	Euphorbiaceae		Dhirii	Leaf	Decoction			[79]
*Phyllanthus maderaspatensis* L.	Euphorbiaceae		Harmel Xixiqaa	All parts	Concoction			
*Rhynchosia erlangeri* Harms	Fabaceae		Harmel	Leaf				
*Lupinus albus* L.	Fabaceae		Gibto	Seed	Juice	Hypertension		[80]
*Ipomoea obscura* (L.)	Convolvulaceae		Lago	Leaf		Heart disease		[81]
*Ocimum laliifolium*	Lamiaceae		Pasi kedo	Leaf				
*Thymus schimperi* Ronniger	Lamiaceae		Tosigne	Leaf	Tea	Hypertension		[82]
*Centaurium pulchellum* (Sw.) Druce	Gentianaceae				Infusion	Diabetes		[83]
*Cleome droserifolia* (Forssk.) Delile	Cleomaceae				Powder			
*Posidonia oceanica* (L.) Del	Posidoniaceae					Obesity		
*Kyllinga monocephala*	Cyperaceae					Diabetes		[84]
*Aloe secundiflora* Engl.	Xanthorrhoeaceae					Hypertension		[85]
*Datura stramonium*	Solanaceae		Asaangra	Leaf, Root	Decoction	Diabetes, Hypertension		[77]
*Mentha piperita*	Lamiaceae		Nana	Leaf	Juice	Hypertension		
*Lagenaria abyssinica*	Cucurbitaceae		Buqe setena	Flower	Powder	Diabetes		
*Indigofera spicata*	Fabaceae			Whole plant		Diabetes		[55]
*Capsium frutescens*	Solanaceae		Nungu	Seed	Maceration	Cardiovascular diseases	Gabon	[86]
Copaifera religiosa	Cesalpiniaceae		Murei	Stem bark	Decoction			
Lygopodium microphyllum	Schizaeaceae		Magol	Leaf				
Senecio gaboneensis	Composeae		Budjambu		Maceration			
Sterculia tragacantha Lindl.	Sterculiacea		Ivostou, Mundundu	Stem bark	Decoction			
Strombosiopsis tetranda Engl.	Olacaeae		Mugamba-malungu					
Cleistopholis glauca	Annonaceae			Stem bark	Decoction	Diabetes		[87]
Copaifera mildbraedii	Caesalpinioideae		Murei	Stem bark	Decoction			
Quassia africana	Simaroubaceae		Mukedji	Stem barks	Maceration			
Annickia chlorantha	Annonaceae		Mfol, Mwamba jaune, Muambebengue	Stem bark	Decoction	Cardiovascular diseases, Diabetes		[86,87,88]
Guibourtia tessmannii (Harms) J.Leonard	Fabaceae		Kevazingo, Kevazigo	Stem bark	Decoction			
*Acacia auriculiformis*	Fabaceae		Akasmani	Leaf	Infusion	Diabetes		[87,88]
*Anonidium mannii* (Oliv.) Eng. & Diels	Annonaceae		Ebom	Stem bark	Decoction			
*Antrocaryon klaineanum*	Anacardiaceae		Onzabili, Osome ele		Infusion, Decoction, Maceration			
*Aucoumea klaineana Pierre*	Burseraceae		Okoume		Maceration			
*Celtis tessmannii*	Cannabaceae		Diania		Decoction			
*Cylicodiscus gabunensis*	Mimosoideae		Okan	Stem bark	Decoction			
*Duboscia macrocarpa*	Malvaceae		Akak	Stem bark	Decoction			
*Eurypetalum tessmannii*	Caesalpinioideae		Anzilim					
*Mammea africana*	Calophyllaceae		Mammea, Oboto	Stem bark	Decoction			
*Microdesmis puberula*	Pandaceae		Inko		Infusion			
*Piptadeniastrum africanum*	Mimosoideae		Dabema, Dabena		Decoction			
*Pseudospondias longifolia*	Anacardiaceae		Ofoss		Decoction, Maceration			
*Santiria trimera*	Burseraceae		Ebo, Nkungu	Root	Decoction			
*Tabernanthe iboga*	Apocynaceae		Iboga, Diboga	Stem bark, Stem root	Maceration, Decoction			
*Alstonia congensis*	Apocynaceae		Mukuka	Root	Decoction, Maceration			[88]
*Buchholzia coriacea*	Capparaceae		Magic cola	Seed	Maceration			
*Bridellia ferruginea*	Euphorbiaceae		Flatsho	Leaf	Decoction	Hypertension	Ghana	[89]
*Luffa cylindrica*	Cucurbiataceae		Kpekplebeshi	Leaf	Decoction	Diabetes, Hypertension		[35]
*Vitex grandifolia*	Lamiaceae		Samanibir	Root	Infusion	Stroke		
*Senna sophera* (L.) Roxb.	Fabaceae		Senna	Leaf	Decoction	High Blood Cholesterol		
*Phyllanthus fraternus* G.L.Webster	Phyllanthaceae		Goyonbaaya	Leaf	Decoction	Diabetes, Hypertension		
*Pileostigma thonningii*	Caesalpinaceae			Leaf		Diabetes	Guinea	[90]
*Ocimum sanctum* L.	Lamiaceae			Leaf, stalk				
*Landolphia heudeloti*	Apocynaceae			Leaf				
*Andansonia digitate*	Bombacaceae			Leaf				
*Cissus aralioide*	Ampelidaceae			Leaf				
*Landolphia dulcis*	Apocynaceae			Leaf				
*Mesonerum benthanmianum*	Caesalpinaceae							
*Ocimum viride* Willd.	Lamiaceae							
*Pterocarpus ericens*	Paplionaceae							
*Nauclea pobeguinii*	Rubiaceae			Leaf, bark				[90,91]
*Citrus medica*	Rutaceae		Katthiou	Leaf, fruits	Infusion	Diabetes, Hypertension		[90,92]
*Anthocleista nobilis*	Gentianaceae		Konibou Kankan, Artaninfiro	Stem bark	Decoction	Diabetes		[93]
*Uapaca togoensis* Pax	Phyllanthaceae		Yalague Pete	Stem bark	Decoction	Hypertension		[92]
*Strophanthus sarmentous* DC	Apocynaceae		Thethe, Teme	Roots		Heart conditions	Guinea-Bissau	[94]
*Bauhinia thonningii* Schum.	Fabaceae		Boa, Mansonca, Mansanca	Roots, Bark				
*Psychotria peduncularis* (Salisb.)	Rubiaceae		Cobodo, Cubedo, Ghupughe	Leaf, Roots				
*Senna didymobotrya*	Fabaceae		Mukengeka	Leaf	Decoction	Diabetes	Kenya	[95,96]
*Warburgia ugandensis* Sprague	Canellaceae							[95]
*Leonotis nepetifolia* (L.) R.Br.	Lamiaceae							
*Toddalia asiatica* (L.) Lam.	Rutaceae							
*Phragmanthera usuiensis*	Loranthaceae		Mondoiwet	Bark	Pounding, Boiling	Stroke		[97]
*Periploca linearifolia* Quart. Dill. & A.Rich.	Apocynaceae		Mwemba-iguru	Stem, Leaf	Decoction	Diabetes		[98]
*Gomphocarpus fruticosus* (L.) W.T.Aiton			Mukangarithi	Seeds, Roots				
*Sonchus luxurians* (R.E.Fr.) C.Jeffrey	Compositae		Muthunga	Leaf	Chewing, Boiling			
*Lactuca inermis* Forssk.								
*Sonchus asper* (L.) Hill								
*Vernonia lasiopus*			Muchata	Leaf	Decoction			
*Spilanthes mauritiana* (A. Rich. ex Pers.) DC.			Gathariaita	Whole plant				
*Dracaena steudneri*	Dracaenaceae		Ithare	Bark, Root				
*Ornithogalum tenuifolium*	Hyacinthaceae		Mugwace	Rhizome				
*Hydnora abyssinica*	Hydnoraceae		Muthigira	Stem				
*Myrsine africana* L.	Myrsinaceae		Mugaita	Fruits	Decoction			
*Olea Africana* Mill.	Oleaceae		Mutero	Leaf, Root				
*Clematis hirsuta* Guill. and Perr.	Ranunculaceae		Mugaya, Ng’undu	Leaf, Roots				
*Prunus africana*	Rosaceae		Muiri	Leaf, Bark				
*Teclea simplicifolia*	Rutaceae		Munderendu	Leaf				
*Grewia similis*	Tiliaceae		Mutheregendu	Leaf	Decoction			
*Typha domingensis* Pers.	Typhaceae		Ndothua	Rhizomes				
*Rotheca myricoides* (Hochst.)	Verbenaceae		Manjugairia	Leaf, Roots, Bark				
*Urtica massaica* Mildbr.	Urticaceae		Thabai/Hatha, Kinyeleelya	Leaf	Decoction	Diabetes		[96,98]
*Euclea divinorum* Hiern.	Ebenaceae		Kikuthi/Mukinyei	Root, Bark	Decoction	Diabetes		[96]
*Aspilia pluriseta* Schweinf	Compositae		Muti/Wuti	Leaf	Decoction			
*Fuerstia africana* T.C.E.Fr.	Lamiaceae		Kalaku	Aerial parts	Decoction			
*Cactus* spp.	Cactaceae		Matomoko	Leaf	Juice			
*Passiflora* spp.	Passifloraceae		Makundi	Leaf	Decoction			
*Eucalyptus* spp.	Myrtaceae		Musanduku	Stem Bark	Decoction			
*Aloe* spp.	Aloeaceae		Kiluma	Leaf				
*Croton megalocarpus* Hutch.	Euphorbiaceae		Muthulu/Kithulu	Leaf	Decoction			
*Ormocarpum kirkii* S. Moore	Fabaceae		Muthii	Leaf	Decoction			
*Passiflora subpeltata*	Passifloraceae		Makundi	Leaf	Decoction	Diabetes		
*Solanum renschii* Vatke	Solanaceae		Mukonda Kondu	Root, Leaf	Decoction	Diabetes		
*Momordica* spp.	Cucurbitaceae		Iphunzu	Leaf	Decoction	Diabetes		
*Euclea racemosa* Murr.	Ebenaceae		Mukinyei	Leaf, Stem bark, Root bark	Decoction	Diabetes		
*Schrebera alata* (Hochst.) Welw.	Oleaceae		Mutoma	Stem bark, Root, Leaf	Infusion	Diabetes		
*Oxygonum sinuatum* (Hochst. & Steud ex Meisn.) Dammer	Polygonaceae		Song’e	Leaf, Whole plant	Infusion, Maceration	Diabetes, Hypertension		[96,99]
*Polyscias fulva*	Araliaceae		Soiyet	Bark	Decoction	Obesity		[100]
*Conyza subscaposa*	Asteraceae		Chepng’ ombet	Leaf, Root	Decoction	Obesity		
*Eriocephalus* sp.	Asteraceae		Sehala-hala sa matlaka	Whole plant		Diabetes, Hypertension	Lesotho	[42]
*Scabiosa columbaria*	Dipsacaceae		Selomi	Leaf, Root		Hypertension		
*Napoleonaea heudelotii* A. Juss.	Lecythidaceae			Leaf, bark, root		Heart failure	Liberia	[101]
*Geophila obvallata* Didr.	Rubiaceae			Leaf		Heart pain		
*Voacanga thouarsii* Roem. & Schult.	Apocynaceae		Kaboky	Leaf, Latex, Roots, Bark, Seeds		Hypertension	Madagascar	[102]
*Lycopodiella cernua* (L.) Pic. Serm	Lycopodiaceae		Tongotsokina	Entire plant		Hypertension		
*Vaccinum* sp.	Vaccinaceae		Voakaramy	Leaf		Diabetes		
*Cyathula uncinulata* (Schrad.) Schinz	Amaranthaceae		Tangogo	Leaf		Diabetes, Cardiac problems		[30]
*Mystroxylon aethiopicum* (Thunb.) Loes.	Celastraceae		Fanazava	Leaf		Hypertension		
*Diospyros* sp.	Ebenaceae		Bois de rose	Bark		Diabetes		
*Psorospermum ferrovestitum* Baker	Hypericaceae		Andriambolamena	Leaf		Hypertension		
*Lycopodium* sp.	Lycopodiaceae		Karakaratoloha	Leaf				
*Pauridiantha paucinervis* (Hiern) Bremek.	Rubiaceae		Tamirova	Leaf		Hypertension, Diabetes		
*Azolla* sp.	Salviniaceae		Ramilamina			Cardiac arrest		
*Lygodium lanceolatum* Desv.	Lygodiaceae		Famatotrakanga	Leaf		Hypertension		[103]
*Securinega seyrigii*	Euphorbiaceae			Bark	Decoction	Hypertension		[104]
*Cedrelopsis grevei*	Ptaeroxylaceae			Bark	Decoction	Diabetes		
*Mimosa pigra* L.	Fabaceae					Cardiovascular disorders		[105]
*Psiadia salviifolia*	Asteraceae			Aerial part		Hypertension		[106]
*Xylopia buxifolia*	Annonaceae			Leaf	Tea	Obesity		[107]
*Gymnosporia divaricata*	Celastraceae		Voasarikely	Leaf		Diabetes		[108]
*Macaranga perrieri*	Euphorbiaceae		Makarangana	Leaf	Decoction	Diabetes		
*Clidemia hirta*	Melastomataceae		Sopatra-Mazambody	Leaf		Hypertension		
*Passiflora foetida*	Passifloraceae		Garana	Leaf	Decoction	Hypertension		
*Fadogia ancylantha* Schweinf	Rubiaceae		Masamba gha Muthondo		Tea	Diabetes, Hypertension	Malawi	[109]
*Fadogia ancylantha*	Rubiaceae		Masamba gha Muthondo	Leaf	Tea	Diabetes		[53]
*Ximenia americana* L.	Olacaceae			Leaf, Stem	Decoction, Powder	Diabetes, Hypertension	Mali	[110]
*Cleome viscosa* Linn.	Capparidaceae	Wild mustard		Root		Cardiac stimulant, Diabetes		[111]
*Boscia senegalensis* Lam.	Capparaceae		Eyzen	Fruits	Maceration, Powder	Diabetes	Mauritania	[112]
*Maerua crassifolia* Forssk.			Atil	Leaf	Powder			
*Combretum glutinosum* Perr.	Combretaceae		Tykefyt		Powder			
*Vachellia tortilis* (Forssk.)	Fabaceae		Talh	Bark	Mashed/Macered	Hypertension		
*Mentha spicata* L.	Lamiaceae		Naana	Leaf	Decoction			
*Ziziphus lotus* (L.) Lam.	Rhamnaceae		Sdar-hreytek		Infusion, Powder, Maceration	Diabetes, Hypertension		
*Camellia sinensis* L.	Theaceae		The vert	Leaf	Infusion	Diabetes, Hypertension, High level of cholesterol	Mauritius	[113,114]
*Celosia cristata*	Amaranthaceae	Coquelicot		Flower	Decoction	Cardiovascular diseases, Hypertension		[115]
*Lycium barbarum*	Solanaceae		Goji	Fruit	Soup	Diabetes		
*Actinida deliciosa*	Actinidiaceae		Kiwi		Juice	Hypertension		[113]
Alisma plantago-aquatica subsp. Orientale (Sam) Sam.	Alismataceae				Infusion	High level of cholesterol		
*Aloysia citriodora* Palau	Verbenaceae		Verveine	Whole plant	Infusion	Cardiovascular disease		
*Anans comosus* (L.) Merr.	Bromeliaceae		Anana	Fruit	Juice			
*Aphloia theiformis*	Aphloiaceae		Fandamane	Leaf	Infusion	Diabetes		
*Apium graveolens* L.	Apiaceae		Celeri		Decoction	Diabetes, Hypertension		
*Artocarpus heterophyllus*	Moraceae		Zack	Fruit		Diabetes		
*Asplenium nidus* L.	Aspleniaceae		Langue de boeuf	Leaf	Infusion	Hypertension		
*Avena sativa* L.	Poaceae		Oatmeal	Grains	Soaking	Diabetes, High level of cholesterol		
*Cardiospermum halicacabum* L.	Sapindaceae		Pocpoc	Leaf	Decoction	Diabetes		
*Cynara cardunculus* L.	Asteraceae		Artichaut	Leaf	Infusion	Diabetes, High level of cholesterol		
*Coriandrum sativum* L.	Apiaceae		Cotomili	Leaf	Infusion	Diabetes		
*Crataegus laevigata* Poir. DC.	Rosaceae		Aubepine			High level of cholesterol		
				Flower		Hypertension		
*Curcuma longa* L.	Zingiberaceae		Safran	Root		Cardiovascular disease		
*Euphorbia heterophylla* L.	Euphorbiaceae		Cacapoule	Flower		High level of cholesterol		
*Cucumis sativus* L.	Cucurbitaceae		Concombre	Fruit	Juice	Diabetes		
*Cucurbita maxima* Duchesne			Giromon		Decoction			
*Eugenia uniflora* L.	Myrtaceae		Rousaille	Leaf	Decoction			
*Faujasiopsis flexuosa* (Lam.)	Asteraceae		Bois cassant					
*Glechoma hederacea* L.	Lamiaceae		Lierre					
*Lactuca sativa* L.	Asteraceae		Laitue	Leaf	Decoction	Diabetes		
*Lagenaria siceraria*	Cucurbitaceae		Calebasse	Fruit				
				Leaf		High level of cholesterol, Hypertension		
*Linum usitatissimum* Linnaeus	Linaceae		Grain de lin	Seeds	Soaking	Diabetes, High level of cholesterol		
*Luffa acutangula* (L.) Roxb.	Cucurbitaceae		Patole	Leaf	Juice	Hypertension, Cardiovascular disease		
*Malus domestica* Borkh.	Rosaceae		Pomme	Fruits	Juice	High level of cholesterol		
*Murraya koenigii* (L.)	Rutaceae		Carripoulet	Leaf	Infusion	Hypertension		
*Ocimum tenuiflorum* L.	Lamiaceae		Tulsi	Leaf	Juice	Diabetes, Hypertension, High level of cholesterol		
*Ophiopogon japonicas* (Thunb.)	Asparagaceae				Infusion with the tea	Diabetes		
*Orthosiphon aristatus* (Blume) Miq.	Lamiaceae		Orthosiphon	Leaf	Decoction, Infusion	Diabetes, Hypertension		
*Phyllanthus emblica* L.	Phyllanthaceae		Amla	Fruit	Consume raw fruit, Juice	Diabetes, High level of cholesterol		
*Piper betle* L.	Piperaceae		Betel	Leaf	Infusion	Diabetes, High level of cholesterol		
*Plantago afra* L.	Plantaginaceae				Decoction	Cardiovascular disease		
*Plantago major* L.			Plantain	Flowers	Juice	Diabetes		
*Prunella vulgaris* L.	Lamiaceae				Infusion	Hypertension		
*Psiloxylon mauritianum*	Myrtaceae		Bigaignon	Leaf	Decoction, Infusion	Diabetes		
*Ravenala madagascariensis* Sonn.	Strelitziaceae		Ravenale	Leaf	Decoction	Diabetes		
*Rubus alceifolius* Poir.	Rosaceae		Piquant loulou	Leaf	Decoction	Diabetes		
*Sigesbeckia orientalis* L.	Asteraceae		Herbe de flacq	Leaf	Decoction	Diabetes		
*Solanum melongena*	Solanaceae		Anguive	Fruit	Cooking	Diabetes		
*Stevia rebaudiana*	Asteraceae		Stevia	Leaf	Infusion	Diabetes		
*Taraxacum officinale* (L.) Weber ex F.H.Wigg.	Compositae		Pissenlit	Roots, Leaf	Decoction, Infusion	Diabetes, High level of cholesterol		
*Vitis vinifera* L.	Vitaceae		Raisin	Seeds	Consume raw seeds	Diabetes		
*Aegle marmelos* L.	Rutaceae		Bael	Leaf	Paste	Diabetes		[116]
*Aloe barbadensis* Mill.	Xanthorrhoeaceae		Aloe vera					
*Triticum aestivum*	Poaceae		Duble, Wheat		Juice	Cardiovascular diseases		
*Orthosiphon stamineus*	Lamiaceae		Orthosiphon			Hypertension		[114]
*Citrus sinensis* (L.) Osbeck	Rutaceae		Orange					
*Metroxylon sagu* Rottb.	Arecaceae		Sagoo					
*Petroselinum crispum* subsp. giganteum (Pau) Dobignard	Apiaceae		Persil			Lower Cholesterol		
*Senna Alexandria* Mill.	Fabaceae		Senne					
*Cassine orientalis*	Celastraceae		Boid d’olive	Leaf		Hypertension		[117]
*Justicia gendarussa*	Acanthaceae		Nitchouli	Leaf		Hypertension		[118]
*Colocasia esculenta* (L) Schott	Araceae		Brede songe	Leaf		Hypertension		
*Euphorbia thymifolia* L.	Euphorbiaceae		Rougette	Whole plant		Hypertension		
*Bruguiera gymnorhiza* Lam.	Rhizophoraceae		Manglier	Root	Infusion, decoction	Diabetes, Hypertension		
*Feronia Limonia* (L) Swingle	Rutaceae		Wood apple	Fruit	Decoction	Diabetes		
*Morinda citrifolia* L.	Rubiaceae		Noni	Fruit, Leaf	Infusion, Juice	Diabetes, High level of cholesterol, Hypertension		[113,114]
*Phoenix dactylifera* L.	Arecaceae		Tam	Leaf	Decoction	Diabetes		[113,119]
*Rhizophora mucronata* Lam.	Rhizophoraceae		Manglier	Root	Decoction, Infusion	Diabetes, Hypertension		[113,118]
*Diplorhynchus condylocarpon*	Apocynaceae			Roots		Diabetes	Mozambique	[120]
*Terminalia stenostachya* Engl. & Diels	Combretaceae			Rosette leaf		Heart disorders, Hypertension, Diabetes		
*Helinus intergrifolius*	Rhamnaceae		Murora			Stroke	Namibia	[121]
*Helinu spartoides*			Omutiwoheva					
*Thevetia peruviana*	Apocynaceae			Bark		Cardiac diseases	Nigeria	[122]
*Vinca alba*	Apocynaceae			Leaf		Hypertension		
*Acalypha godseffiana*	Euphorbiaceae	Copper leaf	Uke	Leaf, seed, fruits	Decoction	Hypertension		[123]
*Brillantaisia patula*	Acanthaceae	Brillantaisia	Idi-ghoko	Leaf, root				
*Bryophyllum pinnatum*	Crassulaceae	Resurrection plant	Ize		Infusion			
*Chasmanthera dependens*	Menispermaceae	Chasmantera	Ukpirialolo	Leaf				
*Combretum racemosun*	Combretaceae	Bush willow	Ajeibolose	Leaf				
*Dichapetalum guineense*	Dichapetalaceae		Uduoifotowo	Root				
*Erythrophleum suaveolens* (Guill. & Perr.) Brenan	Fabaceae	Ordeal tree	Aghoko	Seed				
*Fagara zanthoxyloides*	Rutaceae	Fagara	Ukwe-eghe	Root bark				
*Ficus asperifolia*	Moraceae	Sandpaper	Ebe-amenuwen	Leaf				
*Hibiscus rosa-sinensis*	Malvaceae	Chinese hibiscus	Ireagu	Flower				
*Hibiscus surattensis*		Bush sorrel	Okikhan	Flower, Fruits, Leaf	Decoction, Infusion			
*Phyllantus amarus*	Euphorbiaceae	Carry me seed	Istikini iju ode	Leaf	Infusion			
*Senecio biafrae*	Compositae	English spinach		Root				
*Sorghum caudatum*	Poaceae	Sorghum	Okanibaba					
*Tapinanthus bangwensis*	Loranthaceae	Mistletoe	Ebe ose	Leaf	Decoction			
*Tridax procumbens*	Asteraceae	Coat bottons	Eekule	Aerial plant	Powder			
*Uraria picta*	Fabaceae			Leaf				
*Crataeva adansonii* Oliv.	Capparaceae		Eegun-orun	Leaf		Hypertension		[124]
*Soghum bicolor*	Poaceae		Ese oka	Leaf		Hypertension		[34]
*Beaucamea recurvate*	Ruscaceae		Wowo	Roots				
*Psoropermum febrifugum*	Clusiaceae		Legunoko	Leaf		Stroke		
*Harungana madagascariensis* Lam. ex Poir.	Hyperricaceae		Amuje	Leaf		Hypertension		
*Citropis articulate*	Rutaceae		Atapari obuko	Leaf/roots		Stroke		
*Lablab purpureus* L.	Papilionaceae		Labelabe	Seeds		Heart attack		
*Microdesmis keayana*	Euphorbiaceae		Idi apata	Roots		Stroke		
*Chlorophora excelsa*	Moraceae		Iroko iju	Leaf		Heart attack		
*Thevetia neriifolia*	Apocynaceae		Olomi ojo	Leaf, Bark		Stroke		
*Sarsevieria liberica*	Agavaceae		Oja ikoko	Leaf, Roots		Stroke/Hypertension		
*Loranthus spectobulus*	Loranthaceae	Mistletoe		Leaf	Infusion	Hypertension		[125]
*Entandrophragma utile*	Meliaceae	African cedar	Opepe	Stem bark	Infusion	Diabetes		[126]
*Euphorbia lateriflora*	Euphorbiaceae	Milk cultivars	Enu opire	Stems				
*Khaya ivorensis*	Meliaceae	Lagos Mahogany	Oganwo	Stem bark	Powder			
*Lannea welwitchii*	Anacardiaceae	False Marula	Ekudan					
*Lagenaria breviflora*	Cucurbitaceae	Wild Colocynth	Tagiri	Fruits	Decoction			
*Lawsonia inermis* L.	Lythraceae	Henna tree	Laali	Leaf				
*Sphenocentrum jollyanum*	Menispemaceae	Locus bean	Akerejupon	Roots				
*Tetracera alniflora*	Dilleniaceae	Ware vine	Opon	Fruits				
*Loranthus micranthus* Linn.	Loranthaceae	Eastern Nigeria mistletoe				Diabetes, Hypertension		[127]
*Anthocleista nobilis*	Gentianaceae		Uko nkirisi	Bark, Root	Decoction	Diabetes		[93]
*Commiphora kerstingii* Engl	Burseraceae	Sand paper tree	Ararrabii	Leaf	Maceration	Diabetes		[128]
*Terminalia macroptera* Guill & Perr	Combretaceae	Black afara	Baushe	Root	Decoction	Diabetes		
*Parkia filicoidea* Oliv.	Fabaceae	African locust bean tree	Dorawa	Root	Decoction	Diabetes		
*Odina barteri* Oliv	Anacardiaceae	Olive	Faru	Leaf, Bark	Decoction, Maceration	Diabetes		
*Acacia albida* Delile	Fabaceae		Gardaye	Root	Decoction	Diabetes		
*Acacia macrostachya* DC.	Fabaceae	Winter thorn	Gawo	Root	Decoction	Diabetes		
*Combretum altum* Guill & Perr	Combretaceae		Geza	Root	Decoction	Diabetes		
*Borassus flabellifer* L.	Arecaceae	African fan palm	Giginya	Bark	Decoction	Diabetes		
*Boswellia dalzielii* Hutch	Burseraceae	Frankincense tree	Hano	Root	Decoction	Diabetes		
*Cassia obovata* Collad.	Fabaceae	Neutral henna	Hilisko	Leaf	Maceration	Diabetes		
*Nymphaea odorata* Aiton	Nymphaeaceae	American waterlily	Kainuwa	Leaf	Maceration	Diabetes		
*Piliostigma reticulatum* (DC.)	Caesalpineacea	Camel’s foot	Kalgo	Bark, Root	Decoction	Diabetes		
*Pleurotus tuber-regium*	Pleurotaceae	King tuber mushroom	Katala	Root	Decoction	Diabetes		
*Heeria insignis* Delile Kuntze	Anacardiaceae		Kasheshe	Root	Decoction	Diabetes		
*Crescentia cujete* L.	Bignoniaceae	Calabash	Kwarya	Bark	Infusion	Diabetes		
*Pennisetum pedicellatum* Trin.	Gramineae	Nigeria grass	Kyasuwa	Leaf	Maceration	Diabetes		
*Ziziphus jujube* Linn	Rhamnaceae	Jujube fruit	Magarya	Root	Decoction	Diabetes		
*Sarcocephalus russeggeri* K. ex Sch	Rubiaceae	African peach	Tafashiya	Root	Decoction	Diabetes		
*Detarium senegalense* J.F.Gmel.	Fabaceae	Boire	Taura	Bark, Root	Decoction	Diabetes		
*Combretum sericeum* G. Don.	Combretaceae		Taro	Bark	Decoction	Diabetes		
*Asclepias tuberosa* L.	Asclepiadaceae	Butterfly weed	Yadiya	Leaf, Root	Infusion	Diabetes		
*Clerodendrum volubile*	Lamiaceae	White butterfly	Marugbo, Eweta, Dagba, Obenetete			Diabetes		[129]
*Phragmanthera incana*	Loranthaceae			Leaf		Diabetes, Hypertension		[130]
*Acalypha capitata*	Euphorbiaceae					Hypertension, Hypercholesterolemia		[131]
*Acalypha torta*	Euphorbiaceae					Hypertension		
*Heinsia crinita*	Rubiaceae		Atama	Leaf, Root	Boiling, Crushing, Decoction	Hypertension		[132]
*Indigofera hirsuta* L.	Fabaceae	Hairy indigo	Kai-kai mashekiya	Leaf	Maceration	Diabetes		[55,128]
*Aristolochia ringens* Vahl	Aristolochiaceae	Pelican flower	Akoogun, Akogun	Stem, Root	Decoction	Diabetes, Heart attack		[34,126]
*Aristolochia repens*	Aristolochiaceae	Snake work		Leaf, stem, bark, root		Diabetes, Hypertension		[123,133]
*Citrullus lanatus*	Cucurbitaceae	Water melon	Owori, Kankana	Fruit	Maceration			[123,128]
*Dioscorea bulbifera*	Dioscoreaceae	Aerial potato Air potato	Kamumuwa	Tuber, Root	Decoction			
*Enantia chlorantha*	Annonaceae	African yellow wood	Awopa	Leaf, Stem bark		Hypertension, Stroke		[34,123]
*Gongronema latifolium* Benth.	Apocynaceae	Amaranth globe	Utasi, Madunmaro	Leaf, Stem bark	Boiling, Maceration, Decoction, Infusion, Cooking as soup	Diabetes, Hypertension		[123,126,132,134]
*Icacina trichantha* Oliv.	Icacinaceae		Kamala, Okpokpo, Efikison	Leaf, Seed, Tuber	Crushing, Maceration, Powder	Hypertension		[123,132]
*Talinum triangulare* (Jacq.) Willd.	Talinaceae	Water leaf	Ebe-dondo, Gbure, Gaudi	Leaf, root, bark	Decoction	Diabetes, Hypertension, Stroke		[34,123,128]
*Uvaria afzelii*	Annonaceae	Monkey finger, Scott Elliot	Gbogbonise	Root	Decoction	Diabetes, Hypertension		[123,126]
*Viscum album* L.	Santalaceae	European mistletoe	Ose, Afomo	Leaf, Bark	Decoction, Infusion	Diabetes, Stroke		[34,123]
*Hunteria umbellate* (K. Schum)	Apocynaceae	Husk tomato plant	Osu	Seed, Stem, Bark	Soaking	Diabetes, Hypertension		[123,135]
*Syzygium aromaticum* (L.) Merr. & L.M.Perry	Myrtaceae	Clove bud/Clove	Kanafuru	Seed, Flower	Decoction	Diabetes, Hypertension		[123,126]
*Lantana trifolia* L.	Verbenaceae		Umuhengeri			Heart failure	Rwanda	[136]
*Microglossa vulubilis* DC.	Compositae		Grimbo yufii	Leaf	Infusion	Heart palpitation	Sierra Leone	[137]
*Cymbopogon flexuosus* (Nees ex Steud.) W.Watson	Poaceae	Lemon grass	Lemon grass			Hypertension, Diabetes		[138]
*Garcinia afzelii* Engl	Clusiaceae	Bitter-kola	Yanny			Hypertension		
*Mimosa pudica* L.	Fabaceae	Sensitive plant	Sensitive mimosa			Stroke		
*Microglossa pyrifolia* (Lam.) Kuntze	Compositae			Leaf		Heart disease		[139]
*Agathosma apiculata*	Rutaceae		Ibuchu/Buchu	Roots	Powder	Obesity	South Africa	[140]
*Asparagus africana*	Asaparagaceae	Climbing asparagus	Umthunzi	Leaf	Crushing and Soaking			
*Bulbine alooides* (L.) Willd.	Xanthorrhoeaceae		Irooi water	Roots	Boiling, Infusion			
*Cucumis africanus*	Curcubitaceae	Scaret guord	Ithangazana	Whole plant	Infusion			
Curtisia dentata	Cornaceae	Capelance wood	Umlahleniselefile	Bark	Powder, Boiling			
*Kedrostis africana*	Cucurbitaceae	Baboons cucumber	Uthuvishe	Bulb	Decoction			
*Mimosops obovata*	Sapotaceae	Red milk wood	Umntunzi	Bark	Crushing, Soaking, Infusion			
*Phytolacca dioca* L.	Phytolaccaceae	Phytolacca	Idolo lenkonyane	Leaf	Boiling			
*Rubia petiolaris*	Rubiaceae	Madder	Impedulo	Roots	Infusion			
*Schotia latifolia*	Fabaceae	Forest boer-bean	Umaphipa	Bark	Crushing, Infusion			
*Vernonia mesphilifolia*	Asteraceae	Iron weed	Uhlunguhlungu	Whole plant	Decoction			
*Gethyllis namaquensis*	Amaryllidaceae		Naka tsa tholo	Bulb	Cooking	Diabetes		[141]
*Plumeria obtusa* L.	Apocynaceae		Mohlare wa maswi wa sukiri	Leaf				
*Cussinia spicata*	Araliaceae			Root				
*Helichrysum caespititium*	Asteraceae		Bokgatha, Mabjana, Mmeetse	Whole plant				
*Callilepis laureola*			Phela	Root				
*Lessertia microphylla*	Fabaceae		Mosapelo	Root				
*Hypoxis iridifolia*	Hypoxidaceae		Monna maledu	Tuber				
*Kirkia wilmsii*	Kirkiaceae		Legaba, Modumela		Juice			
*Ficus carica* L.	Moraceae		Mofeiye	Root	Cooking			
*Mimusops zeyheri*	Sapotaceae		Mmupudu	Leaf				
*Englerophytum magalismontanum*			Mohlastwa	Bark				
*Hermannia quartiniana*	Sterculiaceae			Root				
*Triumffeta* sp.	Tilliaceae							
*Dodonaea angustifolia*	Sapindaceae		Ysterhouttoppe	Leaf	Decoction	Chest complaints		[7]
*Hypoxis camerooniana* Baker	Hypoxidaceae		Ikhubalo	Bulb	Chewing	Hypertension		
*Osteospermum imbricatum*	Asteraceae		inkhupuhlana	Bulb, Leaf	Boiling	Chest complaints		
*Phylsalis periviana* L.	Scophulariaeae		Igquzu	Leaf, bulb				
*Vinca major* L.	Apocynaceae					Diabetes		[142]
*Adenopodia spinata*	Fabaceae		Ubobo	Leaf, Root	Maceration	Hypertension		[99]
*Agapanthus africanus*	Amaryllidaceae		Ubani	Leaf, Root	Infusion, Maceration	Hypertension		
*Agave americana*	Asparagaceae			Leaf	Decoction	Hypertension		
*Amaranthus hybridus*	Amaranthaceae			Leaf	Maceration	Hypertension		
*Asystasia gangetica*	Acanthaceae		Isihobo	Leaf	Maceration	Hypertension		
*Canabis sativa*	Cannabaceae		Nsangu	Leaf	Infusion, Decoction	Hypertension		
*Commelina benghalensis*	Commelinaceae		Idangabane	Whole plant	Poultice	Hypertension		
*Crinum macowanii*	Amaryllidaceae		Umdube	Bulb, Leaf, Whole plant		Hypertension		
*Dietes iridioides*	Iridaceae		Isishuphe somfula	Leaf, Root, Rhizomes	Maceration, Infusion	Hypertension		
*Dipcadi brevifolium*	Hyacinthaceae		Ikhakahkha	Bulb	Decoction	Hypertension		
*Dombeya rotundifolia*	Malvaceae		iNhliziyonkhulu	Leaf, Root	Maceration	Hypertension		
*Drimia elata*	Asparageceae		Undongana-zibomvana	Bulb		Hypertension		
*Ekebergia capensis*	Meliaceae		Essenhout	Leaf, Bark		Hypertension		
*Eriobotrya japonica*	Rosaceae			Leaf	Infusion	Hypertension		
*Eriocephalus africanus*	Asteraceae		Kapokbos	Leaf	Infusion	Hypertension		
*Gethyllis* spp.	Amaryllidaceae		Koekoemakranka	Seed, Pod	Maceration	Hypertension		
*Justicia flava*	Acanthaceae		Impela	Leaf	Maceration	Hypertension		
*Leucosidea sericea*	Rosaceae		Umtshitshi			Hypertension		
*Medicago sativa*	Fabaceae			Whole plant	Decoction	Hypertension		
*Mesembryanthemum* spp.	Aizoaceae			Leaf, Stem	Decoction, Maceration	Hypertension		
*Oldenlandia affinis*	Rubiaceae		Umampeshane	Root	Decoction	Hypertension		
*Peucedanum galbanum*	Apiaceae		Droedas	Leaf	Infusion	Hypertension		
*Physalis viscosa*	Solanaceae			Leaf	Maceration	Hypertension		
*Protorhus longifolia*	Anacardiaceae		Uzintlwa	Leaf, Bark	Maceration	Hypertension		
*Rauvolfia caffra*	Apocynaceae		umHlambamanzi	Stem, Bark, Whole plant		Hypertension		
*Rhus chirindensis*	Anacardiaceae		Umhlabamvudu	Leaf, Fruit, Bark, Twig, Root	Maceration	Hypertension		
*Scolopia mundii*	Salicaceae		iHlambahlale	Bark		Hypertension		
*Senecio bupleuroides*	Asteraceae		Isiqandamatshana			Hypertension		
*Senecio inornatus*	Asteraceae		Uhlabo	Root	Decoction	Hypertension		
*Spermacoce natalensis*	Rubiaceae		Umabophe	Leaf, Bark, Root		Hypertension		
*Stangeria eriopus*	Zamiaceae		Umfigwani	Leaf, Root		Hypertension		
*Trifolium africanum*	Fabaceae		Wildeklawer	Whole plant	Infusion	Hypertension		
*Turraea floribunda*	Meliaceae		Umadlozane	Bark, Leaf, Root	Infusion	Hypertension		
*Valeriana capensis*	Valerianaceae		Wildebalderjan	Rhizome, Root		Hypertension		
*Pelargonium antidysentericum*	Geraniaceae		Rooistorm	Root		Diabetes		[143]
*Pteronia divaricata*	Asteraceae				Tea	Diabetes		[144]
*Elaeodendron transvaalense*	Celastraceae			Stem bark	Tea	Diabetes		
*Warburgia salutaris* (G. Bertol.) Chiov	Canellaceae	Pepperbark tree	Peperbasboom, isibhaha			Diabetes		[145]
*Lannea edulis Sond.*	Anacardiaceae	Wild grape	Pheho, muporotso	bark	Decoction	Diabetes		
*Kedrostis nana*	Cucurbitaceae		Kalmoes, Serekola	Root, Tuber	Decoction, Infusion	Diabetes, Hypertension		[146,147]
*Herichrysum odoratissimum*	Asteraceae		Imphepho	Whole plant	Crushing, Boiling, Infusion	Diabetes		[53]
*Herichrysum nudifolium*	Asteraceae		Ichocholo	Leaf, Root	Boiling	Diabetes		
*Herichrysum petiolare*	Asteraceae		Imphepho	Whole plant	Crushing, Boiling	Diabetes		
*Bulbine frutescens*	Apocynaceae		Ibhucu	Root	Boiling, Infusion	Diabetes		
*Heteromorphica arborescens*	Apiaceae		Umbangandlala	Leaf, Root	Boiling	Diabetes		
*Chilianthus olearaceus*	Buddlejaceae		Umgeba	Leaf, Twig	Infusion	Diabetes		
*Athrixia phylicoides*	Asteraceae	Bush tea			Infusion	Diabetes, Hypertension, Heart problems		[148]
*Aloe microstigma* Salm-Dyck	Xanthorrhoeaceae		Bitteraalwyn, aalwyn	Leaf		Diabetes		[149]
*Asclepias crispa*	Apocynaceae		witvergeet, witstorm	Root	Decocotion	Diabetes		
*Aspalathus linearis*	Fabaceae				Infusion	Hypertension		
*Dittrichia graveolens*	Asteraceae		kakiebos	Leaf	Infusion	Diabetes, Hypertension		
*Pteronia cinerea*	Asteraceae		boegoe	Leaf	infusion	Diabetes		
*Salvia dentata* Aiton	Lamiaceae		bloublomsalie	Leaf	infusion	Diabetes		
*Viscum capense*	Viscaceae		groen voëlent	Stem	infusion	Diabetes		
*Acokanthera oblongifolia* (Hochst.) Codd	Apocynaceae		Inhlungunyemba			Diabetes		[147]
*Acorus calamus* L.	Acoraceae		Kalmoes		Infusion			
*Adenia digitata* (Harv.) Engl	Passifloraceae		Uthangazane					
*Aloe greatheadii* (Schonland)	Xanthorrhoeaceae	Spotted aloe	Transvaalaalwyn, grasaalwyn	Leaf	Decoction			
*Aloe maculata* All.	Xanthorrhoeaceae	Soap aloe	bontaalwyn	Leaf				
*Arctopus echinatus* L.	Apiaceae		Platdoring	Root				
*Asclepias crispa* P.J. Bergius	Apocynaceae		Witvergif, witvergeet					
*Asclepias fruticosa* L.	Apocynaceae	African milkweed	ulusinga, lwesalukazi	Dried roots	Infusion			
*Brachylaena elliptica* (Thunb.) DC.	Asteraceae	Bitter-leaf, bitterleafed silver oak, fire sticks	Uhlunguhlungu, isiduli, isagqeba	Leaf	Decoction			
*Brachylaena ilicifolia* (Lam.) Phill. and Schweick.	Asteraceae	Small bitter-leaf	Fynbitterblaar, igqeba	Leaf	Decoction			
*Bridelia micrantha* (Hochst.) Baill	Phyllanthaceae	Coastal goldenleaf	mitserie, bruin stinhhout	Bark	Decoction			
*Buddleja salviifolia* (L.) Lam.	Scrophulariaceae	Sagewood, butterfly bush	igwangi, iloshane, ilothane, mupambati					
*Bulbine latifolia* (L.f.) Spreng.	Xanthorrhoeaceae	Red carrot	rooiwortel	root				
*Cnicus benedictus* L.	Asteraceae	Holy thistle			Infusion			
*Dittrichia graveolens* (L.) Greuter	Asteraceae		Kakiebos	Leaf, twigs	infusion			
*Dodonaea viscosa* (L.) Jacq.	Sapindaceae	Sand olive	sandolien, ysterbos	leaf	Boiling			
*Empodium plicatum* (Thunb.) Garside	Hypoxidaceae	Golden star						
*Eriocephalus ericoides* (L.f.) Druce	Asteraceae		Kapokbos, wilderoosmaryn					
*Eriocephalus punctulatus* DC.	Asteraceae	Wild rosemary	kapokbos	Leaf	Decoction			
*Eriocephalus tenuifolius* DC.	Asteraceae		Sehalahala-sa-matlaka					
*Eucalyptus citriodora* Hook.	Myrtaceae	Lemon-scented gum			Extract			
*Euclea crispa* (Thunb.) Gürke	Ebenaceae	Guarri bush	idungamuzi, umgwali	Root	Powder			
*Euphorbia prostrata* Aiton	Euphorbiaceae		Harige kruipmelkkruid					
*Galium tomentosum* Thunb	Rubiaceae		Rooihoutjie	Root				
*Gazania krebsiana* Less	Asteraceae		Gousblom, botterblom	leaf	Decoction			
*Grewia flavescens* Juss.	Malvaceae	Sandpaper raisin	Mopharantshone	Roots				
*Grewia villosa* Willd.	Malvaceae	Mallow raisin	Mopharantshone	Roots				
*Gomphrena celosioides* Mart.	Amaranthaceae	Soft khaki weed	Lebolomo la naga	Roots				
*Haplocarpha scaposa* Harv.	Asteraceae	False gerbera	melktou	Leaf, roots	Extract			
*Helichrysum caespititium* (DC.) Sond. ex Harv.	Asteraceae		Mokgata	Whole plant	Infusion			
*Hermannia cuneifolia* Jacq.	Malvaceae		Wilde heuning, geneesbossie	Leaf	Infusion			
*Hermannia pinnata* L.	Malvaceae	Doll’s rose	kwaasblaar, kruip poprosie					
*Hoodia currori* (Hook.) Decne.	Apocynaceae	Hoodia cactus, bitter ghap	bergghaap, bokhorings	Stem				
*Inula graveolens* (L.) Desf.	Asteraceae		Khakibos	Foliage				
*Jacobaea maritima* (L.) Pelser & Meijden	Asteraceae		Vaalbos					
*Kedrostis africana* (L.) Cogn.	Cucurbitaceae		Bojaankamoo					
*Limeum aethiopicum* Burm. f.	Limeaceae		Koggelmandervoet, boesmandagga		Infusion			
*Merwilla plumbea* (Lindl.) Speta.	Asparagaceae		Setsusha	Leaf				
*Mimulus gracilis* R.Br.	Phrymaceae				Decoction			
*Morella serrata* (Lam.) Killick	Myricaceae	Lance-Leafd strawberry, waxberry	Iyethi, ulethi, umakhuthula	Root	Decoction			
*Notobubon galbanum* (L.) Magee	Apiaceae	Berg celery, blister bush	bergseldery	Foliage				
*Nymphaea caerulea* Savigny	Nymphaeaceae	Blue water lily	blouwaterlelie	Seeds				
*Opuntia vulgaris* Mill.	Cactaceae	Prickly pear		Leaf	Decocotion			
*Pegolettia baccharidifolia* Less.	Asteraceae		Ghwarrieson, gwarrieson, heuningdou	Foliage				
*Pentzia incana* (Thunb.) Kuntze	Asteraceae		ankerkaroo, kleinskaapkaroobos					
*Pittosporum viridiflorum* Sims	Pittosporaceae		Umkhwenkhwe	Bark				
*Portulacaria afra* Jacq	Didiereaceae		Spekbosb, spekboomblareb,		Eaten			
*Pteronia cinerea* L.f.	Asteraceae		Boegoe, silverboegoe	Leaf	Infusion			
*Salix mucronata* Thunb.	Salicaceae		Rivierwilger, rivierwiller	Foliage				
*Salvia africana-lutea* L.	Lamiaceae	Red sage		Foliage				
*Salvia dentata* Aiton	Lamiaceae	Toothed sage	bergsalie, blousalie		Syrup			
*Salvia microphylla* Kunth	Lamiaceae		Rooiblomsalie, pienblomsalie					
*Stachys hyssopoides* Burch. ex Benth	Lamiaceae	Hyssop-Leafd hedge nettle						
*Thesium lineatum* L.f. LEP	Santalaceae	Black storm	swartstrom	Root				
*Tinospora fragosa* Verdoorn & Troupin	Menispermaceaea	Aaron’s rod	Makgonatsohle	Leaf and stem	Boiling			
*Viscum capense* L. f.	Santalaceae	Cape mistletoe	groen voelent, taaibos	Stem	Infusion			
*Viscum continuum* E. Mey. ex Sprague	Santalaceae		Voëlent, litjiestee		Infusion			
*Alepidea amatymbica*	Apiaceae	Larger tinsel flower	Igwili/Umvuthuza, Ikhathazo	Rhizome, Roots	Powder	Hypertension, Obesity		[99,140]
*Cissampelos capensis*	Menispermaceae	David root	Umayisake, Idabulitye, Fynblaarklimop, Dawidtjiewortel	Leaf, Roots	Crushing, Infusion	Diabetes, Heart problem, Hypertension, Obesity		[7,99,140,142,147,150]
*Exomis microphylla* (Thunb.) Aellen	Amaranthaceae	Sugar beet	Umvawenyathi, Hondebossie	Leaf	Decoction	Diabetes, Obesity		[140,147]
*Leonotis ocymifolia*	Lamiaceae		Umuncwane, Wildedagga	Whole plant	Crushing, Boiling, Infusion			
*Leonotis leonurus*	Lamiaceae		Umunyamunya Umfincafincane Wilde dagga	Bulb, Leaf, Root, Flower, Stem, Whole plant	Crushing, Boiling, Infusion, Decoction	Obesity, Diabetes, Hypertension, Other Cardiovascular ailments		[2,7,99,140,143,149]
*Carpobrotus edulis* (L.)	Aizoaceae	Sour fig, Cape fig, Hottentot’s fig	Lepolomo la go naba, Suurvye, Hotnotsvye, Vyerank, Kaapvy	Leaf, Fruit, Sap	Juice, Decoction	Diabetes		[141,143,147,149]
*Opuntia ficus-indica* (L.) Mill.	Cactaceae	Indian pear, Indian fig, Sweet prickly pear	Motloro, Umthelekisi,	Leaf, Root	Cooking, Crushing, Decoction	Diabetes, Hypertension		[99,141,147]
*Agathosma betulina*	Rutaceae		iBuchu, Regteboegoe	Leaf, Stem	Infusion	Hypertension		[7,99]
*Rhoicissus digitata*	Vitaceae		Uchithithibuna, UmNangwazi	Bulb, Tuber	Infusion			
*Geranium incanum* Burm.f.	Geraniaceae		Tlako, Vrouetee	Leaf, Stem	Boiling, Decoction	Heart problem, Hypertension		
*Gunnera perpensa*	Gunneracea	River pumpkin, Wild rhubarb	iPhuzi lomlambo, Qobo, Igobho	Bulb, Leaf, Root, Rhizome	Infusion, Decoction	Diabetes, High cholesterol		[7,147]
*Lichtensteinia lacera* Cham. & Schltdl.	Apiaceae		iQwili, Kaalmoes, Kalmiswortel	Leaf, bulb, Stem	Boiling, Infusion	Chest complaints, Diabetes, Hypertension		[7,99,143]
*Ruta graveolens* L.	Rutaceae	Rue, Common rue, Herb-of-grace	Gwabeni or iVendrit, Wynruit, Wynruik	Leaf	Infusion	Diabetes, Heart disease, Hypertension		[7,99,147,149]
*Sutherlandia frutescens* (L.) R.Br.	Fabaceae	Cancer bush	UmNwele, Petola, Lerumo-lamadi, Koorsbos, Kalkoentjiebos	Leaf	Boiling, Infusion, Decoction	Diabetes, Hypertension		[7,149,151,152]
*Tulbaghia violacea* Harv.	Amaryllidaceae	Wild garlic	Itswele lomlambo, Wilde knoffel	Bulbs, Leaf, Root, Rhizome	Boiling, Infusion, Decoction	Diabetes, Hypertension, Heart problems, High cholesterol		[7,99,143,147]
*Catha edulis* (Vahl) Forrsk.	Celastraceae		Umhlwazi	Leaf	Maceration	Diabetes, Hypertension		[99,142]
*Chironia baccifera* L.	Gentianaceae	Christmas berry	Bitterbos, Aambeibossie	Leaf, Whole plant	Decoction, Infusion	Diabetes		[142,147,149]
*Acokanthera oppositifolia*	Apocynaceae		Inhlungunyembe	Leaf, Stem, Root	Maceration	Diabetes, Hypertension		[99,147]
*Commelina africana*	Commelinaceae	Yellow Commelina	Idangabane, Geeleendagsblom	Whole plant	Decoction			
*Helichrysum crispum*	Asteraceae		Hotnotskooigoed, Kooigoedbos	Leaf	Infusion			
*Rapanea melanophloeos* (L.) Mez	Primulaceae	Cape beech	iKhubalwane, Boekenhout	Bark				
*Teedia lucida*	Scrophulariaceae		Hlwenya, Stinkbos					
*Urtica urens*	Urticaceae			Leaf	Infusion, Decoction			
*Albertisia delagoensis*	Menispermaceae		Umgandaganda, Ohumane	Leaf, Stem, Root, Rhizome	Decoction, Boiling	Hypertension		[99,153]
*Carpobrotus dimidiatus*	Mesembryanthemaceae		Ikhambi lamabulawo	Leaf, Stem, Fruit	Decoction			
*Citrullus lanatus*	Curcurbitaceae		Bitterwaatlemoen, Ibhece	Fruit, Seed, Leaf	Decoction. Tea			
*Cladostemon kirkii*	Capparaceae		umThekwini, Usdumbo	Stem, Bark, Root	Maceration, Decoction			
*Hyphaene coriacea*	Arecaceae		iLala	Root				
*Hypoxis argentea*	Hypoxidaceae		Inongwe, Labateka	Corm, Tuber	Decoction			
*Lippia javanica* (Burm.f.) Spreng.	Verbanaceae		Umsuzwane	Leaf	Decoction			
*Ozoroa engleri*	Anacardiaceae		Isifico, Isfico	Leaf, Bark, Root	Decoction			
*Ptaeroxylon obliquum*	Rutaceae		umThathi, Umsango	Root	Maceration			
*Sarcophyte sanguinea*	Balanophoraceae		Umvumbuka, Umavumbuka	Stem, Root, Whole plant	Decoction, Infusion			
*Sarcostemma viminale*	Apocynaceae		Umbelebele, Umbhelebhele	Stem, Aerial parts, Twigs	Infusion			
*Senecio serratuloides*	Asteraceae		Ichazampukane, Unsukumbili	Leaf, Stem	Decoction			
*Strychnos madagascariensis*	Strychnaceae		umkwakwa	Seed, Fruit, Bark, Root	Powder			
*Tetradenia riparia*	Lamiaceae		Ibozane	Leaf, Seed	Decoction			
*Ballota africana*	Lamiaceae		Kattekruide, Kattekruid, Salie	Leaf	Infusion	Diabetes, Hypertension		[99,143,147]
*Chrysocoma ciliata*	Asteraceae		Kaalsiektebos, Bitterbos	Leaf, Root	Decoction			
*Lessertia frutescens*	Fabaceae	Cancer bush, Balloon pea	Umnwele, Kankerbos, Bitterbos, Kiertjies, Blaasiebos	Leaf, Shoot	Decoction, infusion			
*Cadaba aphylla*	Capparaceae		Bobbejaanarm, Swartstorm, Stormwortela, Swartstorm	Leaf, Stem, Root	Infusion			[99,143,147]
*Convolvulus capensis*	Convolvulaceae		Skaapklimop, Bitterpatat	Bulb	Decoction	Diabetes, Hypertension		[99,143]
*Crassula muscosa*	Crassulaceae		Skoenvetebos, Akkedisbos	Leaf, Stem, Root, Flower	Decoction			
*Dicerothamnus rhinocerotis* (L.f.) Koek.	Compositae		Ranosterbos	Leaf, Stem				
*Euryops abrotanifolius*	Asteraceae		Bergharpuisbos, Harpuisbos	Leaf, Stem				
*Hoodia gordonii*	Apocynaceae		Bobbejaanghaap, Bitterghaap	Leaf, Stem				
*Diosma oppositifolia*	Rutaceae		Bitterboegoe, Skaapbos	Leaf, Stem, Flower		Hypertension		
*Salvia africana-caerulea*	Lamiaceae		Wildesalie, Blousalie, Blou blomsalie	Twig, Leaf	Infusion			
*Sceletium tortuosum*	Aizoaceae		Tandtrekbos, Kougoed	Leaf, Root				
*Conyza scabrida*	Asteraceae		Umanzimnyama, Vleiwilger, Fonteinbos, Medisynebos	Leaf, Foliage	Decoction, Infusion	Diabetes Hypertension		[99,147,149]
*Elytropappus rhinocerotis*	Asteraceae	Rhinosaurous bush	Renosterbos, Anosterbos, Bergrenoster, Vaalrenoster	Leaf	Decoction, Infusion			
*Dicoma capensis*	Asteraceae		Koorsbossie, Hosabie, Baarbos, Kaarmadik, Sandsalie	Leaf, Root, Twig	Decoction	Diabetes, Hypertension		[99,149]
*Euclea undulata*	Ebenaceae		Inkunzane	Bark, Root, Whole plant	Infusion, Tea	Diabetes, Hypertension		[99,144]
*Warburgia salutaris*	Canellaceae	Pepperbark tree	Isibhaha	Stem Bark, Root				
*Hypoxis colchicifolia* Baker	Hypoxidaceae		IIabatheka, Inongwe	Bulb, Corm	Boiling, Crushing	Diabetes, Hypertension		[53,99]
*Searsia burchellii*	Anacardiaceae		Karookoeniebos, Taaibos	Leaf, Stem, Root	Infusion	Diabetes, Hypertension		[99,143]
*Mentha longifolia*	Lamiaceae	Wild mint	Ufuthanalomhlanaga, Ballerja	Leaf, Stem	Decoction, Infusion	Diabetes, Hypertension		[99,143,147,149]
*Schkuhria pinnata*	Asteraceae	Dwarf Mexican Marigold	Ruhwahwa, Kleinkakiebos	Whole plant, Leaf, Root	Boiling, Decoction, Infusion	Diabetes, Hypertension		[99,144,147]
*Gnidia deserticola*	Thymelaeaceae		Koorsbos	Leaf, Stem, Root		Diabetes		[143]
*Tagetes minuta*	Asteraceae		Koebiebos	Leaf				
*Tylecodon paniculatus*	Crassulaceae		Bees se bal	Leaf, Stem				
*Bulbine natalensis* Baker	Xanthorrhoeaceae		Ibhucu, Rooiwortel	Leaf, Stem, Root	Boiling, Infusion	Diabetes		[53,154]
*Lupinus termis* L.	Papilionaceae		Tormos, Tumus	Seeds, Fruit		Diabetes	Sudan	[155,156]
*Combretum* sp.	Combretaceae		Habeil					[157]
*Solenostemma argel* (Del.)	Asclepiadaceae		Hargel	Leaf		Diabetes		
*Geigeria alata* Benth. & Hook.f. ex Oliv. & Hiern.	Compositae		Gud-gat, Gadad, Al Gadad	Whole plant, Aerial part, Root	Decoction, Infusion	Diabetes, Hypertension		[156,158,159]
*Bauhinia reticulata* DC.	Fabaceae		Khroob	Fruit	Maceration	Hypertension		[159]
*Blepharis linanifolia*	Acanthaceae		Bagail	Aerial part	Maceration, infusion	Diabetes, Hypertension		
*Boswellia papynifera*	Burseraceae		Tarag tarag	Bark	Maceration	Diabetes		
*Cymbopogan schoenanthus* (L.)	Poaceae		Mahraib	Aerial part	Maceration, infusion			
*Maerua pseudopetalosa*	Capparaceae		Kurdala	Root	Mastication	Diabetes, Hypertension		
Sonchus cornutus	Compositae		Moleata	Leaf	Infusion	Diabetes		
*Bauhinia rufescens*	Fabaceae		Kulkul	Leaf		Diabetes		[156]
*Catunaregam nilotica*	Rubiaceae		Kir Kir	Fruit				
*Cicer arietinum* L.	Fabaceae		Kabkabe	Seed				
*Cinnamomum verum*	Lauraceae		Gerfa	Stem bark				
*Cyperus rotundus* L.	Cyperaceae		Sieda	Rhizome				
*Faidherbia albida*	Fabaceae		Haraz	Root bark				
*Foeniculum vulgare* Mill.	Apiaceae		Shamar	Fruit				
*Rhynchosia minima*	Fabaceae		Irg el Dam	Root				
*Senna obtusifolia*	Caesalpiniaceae		Kawal	Leaf				
*Zygophyllum coccineum* L.	Zygophyllaceae		Tartir	Whole plant				
*Hibiscus rabdariffa* L.	Malvaceae		Karkade	Sepals	Maceration, Decoction	Hypertension		[160]
*Aloe sinkatana* Reynolds	Xanthorrhoeaceae		Sabar, Al-Sabar	Leaf	Mucilaginous, Paste	Diabetes		[156,157,160]
*Salvadora persica* L.	Salvadoraceae		Arak, Al-Miswak	Fruits, Stem, Leaf	Maceration	Hypertension		[157,160]
*Tinospora bakis*	Menispermaceae		Bun balash/irg alhagar, Irg El Haggar	Root, Seed	Maceration, infusion	Diabetes		[156,159]
*Aloe saponaria*	Asphodelaceae		Lihala	Leaf	Decoction	Cardiac problems	Swaziland	[161]
*Vernonia glabra*	Asteraceae		Linyatselo	Root	Decoction	Diabetes		[53,161]
*Ozoroa reticulate* (Bak.f.)	Anacardiaceae		Nago	Root, Stem bark	Boiling	Hypertension	Tanzania	[162]
*Cissus rotundifolia*	Vitaceae		Chazi	Leaf	Juice	Heart troubles		
*Aerangis flabellifolia*	Orchidaceae		Kinmba	Roots	Decoction	Heart diseases		[163]
*Sterculia appendiculata* K. Schum	Sterculiaceae		Mgude	Stem bark	Decoction	Diabetes		[164]
				Leaf		Cardiac pains		
*Cymbopogon citrullus*	Poaceae	Lemongrass	Mchaichai	Leaf, Stem, Oil	Extract	Diabetes		[165]
*Hagenia abyssinica* (Bruce ex Steud.) J.F.Gmel.	Rosaceae	African redwood, East African rosewood	Enjani engashe	Flower and Leaf	Extract			
*Afzelia quanzensis* Pers.	Caesalpiniodeae		Mkola	Roots, bark		Stroke		[166]
*Albizia harveyi* Fourn.	Mimosoideae		Mupogolo	Roots, Leaf		Hypertension		
*Boscia salicifolia* Oliv.	Capparidaceae		Muguluka	Roots, bark		Stroke		
*Dalbergia nitidula* Bak.	Papilionoideae		Kafinulambasa			Diabetes		
*Ekebergia benguelensis* DC.	Meliaceae		Mutuzya	Roots, Leaf, bark		Hypertension		
*Pericopsis angolensis* (Bak.)	Papilionoideae		Mubanga, Muvunga	Roots, Leaf, bark		Stroke		
*Vitex mombassae* Vatke	Lamiaceae		Mutalali, Musungwi	Roots, Leaf		Diabetes		
*Xylopia odoratissima* Oliv.	Annonaceae		Mushenene					
*Elaeodendron schlechteranum* (Loes.)	Celastraceae		Chihusilo, Ngakama	Stem bark, Root, Decoction	Boiling	Cardiovascular problems, Hypertension		[162,167]
*Deinbollia borbonica* Scheff.	Sapindaceae		Mpwakapwaka, Mwenda kuzimu, Mmoyomoyo, Mkuyu, Muhunge	Fresh Leaf, Root	Soup	Diabetes, Increased heart beat		[168,169]
*Vepris glomerata* (F. Hoffm.)	Rutaceae		Mulungusigiti	Roots, Leaf		Diabetes, Stroke		[63,166]
*Aspilia mossambiscensis* (Oliv.) Wild	Compositae			Leaf	Boiling, Decoction	Diabetes		[169]
*Bridelia* *duvigneaudii* J. Leonard	Euphorbiaceae		Msalasi	Roots	Boiling			
*Cyphomandra crassifolia* Kuntze	Solanaceae		Mtomatoma	Seeds	Powder			
*Cyphostemma junceum* (Bak.) Descoings	Vitaceae		Mhogo mwitu	Roots				
*Dioscorea* praehensilis Benth.	Dioscoriaceae		Amasoma	Tuber	Cooking			
*Ficus fischeri* Mildbr. & Burr.	Moraceae		Mvila	Stem bark	Boiling			
*Maprounea* *africana* Müll. Arg.	Euphorbiaceae		Mtunu	Roots	Decoction			
*Pseudolachnostylis* *maprouneifolia* Pax	Phyllanthaceae		Mwana	Roots	Decoction			
*Sorindeia madagascariensis* DC.	Anacardiaceae		Mpilipili	Roots	Boiling			
*Uvariopsis bisexualis* Verdc.	Annonaceae		Ntaa	Roots, Leaf	Decoction			
*Chassalia umbraticola* Vatke	Rubiaceae		Mkangaa					
*Ochna confusa* Burtt Davy and Greenway	Ochnaceae		Mnungamo	Root, bark				
*Warburgia stuhlmannii* Engl.	Canellaceae		Sokonoi	Root and bark				
*Terminalia mollis* M. Laws	Combretaceae		Mkelenge	Stem bark				
*Zanthoxylum* *acanthopodium* DC.	Rutaceae		Mjafari, oloisuki	Root and bark				
*Acacia kirkii* Oliv.	Mimosaceae		Mgunga					
*Acacia tortilis* (Forsk) Hayne	Mimosaceae		Kikwesa, chikwesa	Roots				
*Albizzia anthelmintica* Brogn.	Mimosaceae		Mkutani	Roots				
*Bridelia micrantha* Bertol	Euphorbiaceae		Kitovutovu	Roots				
*Caesalpinia* *bonduc* (L.) Roxb	Caesalpiniaceae		Msoro, mkomve					
*Cyanoglosum lanceolatum* Forsk	Boraginaceae		Kifasa	Roots				
*Cyphostemma buchananii* (Planch.) Desc. ex Wild & R. B.Drumm.	Vitaceae		Kigugire, kigugile kidogo	Roots and Leaf				
*Diospyros loureiroana* G.Don	Ebenaceae		Mgoto Syn.	Roots				
*Lannea schimperi* (A.Rich.)	Anacardiaceae		Mbumbu nyika	Stem bark				
*Markhamia obtusifolia* Bak Spragne	Bignoniaceae		Myuyu					
*Piliostigma* *tortuosum* (Collett & Hemsl.) Thothathri	Fabaceae		Milneedh					
*Raphia hookeri*	Arecaceae		Bambou			Hypertension	Togo	[170]
*Combretum molle*	Combretaceae		Kizizikou			Hypertension		
*Monotes kerstingii*	Dipterocarpaceae		Sere			Diabetes		
*Lonchocarpus cyanescens*	Fabaceae		Tchele			Diabetes		
*Piliostigma thonningii*			Bakou			Diabetes, Hypertension		
*Xeroderris stuhlmannii*			Tchalawari			Diabetes, Hypertension		
*Hyptis suaveolens*	Lamiaceae		Botifadini			Hypertension		
*Securinega virosa*	Phyllanthaceae		Tchakatchaka			Diabetes, Hypertension		
*Ficus sur* Forssk.	Moraceae		Kilimaou	Root	Powder	Hypertension		[171]
*Pergularia daemia* (Forssk.)	Asclepiadaceae		Kponkeke, Kpankeke			Diabetes		[172]
*Lactuca taraxacifolia* (Willd.)	Asteraceae		Anonto	Root	Decoction	Diabetes, Hypertension		
*Alternanthera penguns*	Amaranthaceae		Agbaklin	Whole plant	Decoction	Diabetes		[173]
*Dracaena arborea*	Agavaceae		Agnanti	Leaf	Decoction	Diabetes		
*Anchomanes welwitschii*	Araceae		Haduku	Rhizome	Decoction	Diabetes		
*Mondia whitei*	Asclepiadaceae		Kanabo	Root	Decoction	Diabetes		
*Conyza aegyptiaca*	Asteraceae		Dagnigbe Ahlonme	Whole plant	Decoction	Diabetes		
*Launaea taraxacifolia*	Asteraceae		Anonto		Decoction	Diabetes		
*Sida linifolia*	Malvaceae		Odoe-Ogbougbo	Whole plant	Decoction	Diabetes		
*Tiliacora warneckei*	Menispermaceae		Katokpan	Root	Decoction	Diabetes		
*Cocoloba uvifera*	Polygonaceae		Raisin de mer	Leaf	Decoction	Diabetes		
*Paulinia pinnata*	Sapindaceae		Agbatsalika	Leaf	Decoction	Diabetes		
*Solanum aethiopicum*	Solanaceae		Agbissan	Leaf + Fruit	Decoction	Diabetes		
*Tectona grandis*	Verbenaceae		Tecti	Leaf	Decoction	Diabetes		
*Terminalia glaucescens*	Combretaceae		Anagossitou, Souwadaou	Leaf	Decoction	Diabetes		[170,173]
*Millettia thonningii* (Schum. & Thonn.) Baker	Fabaceae		kodoliya	Root	Powder, Decoction	Diabetes, Hypertension		[170,171]
*Canarium schweinfurthii* Engl.	Burseraceae		Muwafu, Empafu, Mbafu	Seed, Bark	Decoction, Pasting, Chewing	Cardiovascular condition, Hypertension, Diabetes	Uganda	[38,174]
*Garcinia buchananii* Baker	Clusiaceae		Musali, Ensali, Nsaala	Bark, Leaf, Roots	Decoction, Pounding	Cardiovacular condition, Diabetes, Hypertension		
*Tragia benthamii* Baker	Euphobiaceae		Kamyu	Root	Pounding	Hypertension		[38]
*Acacia constricta* Benth.	Fabaceae		Muwelamanyo		Decoction	Diabetes		
*Sesbania sesban* (L.) Merr.			Muzimbandeya			Hypertension, Diabetes		
*Ficus cyathistipula* Warb.	Moraceae		Mubembe	Leaf	Decoction	Hypertension		
*Morella kandtiana* (Engl.)	Myricaceae		Mukikimbo	Roots	Chewing	Hernia of the heart		
*Oxalis corniculata* L.	Oxalidaceae		Kajjampuni	Leaf		Hypertension, Diabetes		
*Aframomum anguistifolium*	Zingiberaceae		Mantugulu	Roots	Pounding	Obesity		
*Pancovia* sp.	Sapindaceae			Stem, Roots	Pounding	Heart-related problems		[175]
*Senencio nandensis*	Asteraceae		Kisenda	Leaf	Decoction	Hypertension		[31]
*Hallea rubrostipulata*	Rubiaceae		Muziku	Roots		Diabetes		
*Brachiaria decumbens* Stapf.	Poaceae		Ejubwa	Fresh leaf	Chewing, Decoction	Heart disease		[176]
*Musa* sp.	Musaceae		Bitooke	Fresh fruit, Fresh leaf	Decoction	Hypertension		
*Zanthoxylum gilletii* (De wild.)	Rutaceae		Mutatembwa	Fresh stem bark	Infusion			
*Solanum anguivi*	Solanaceae		Katunkuma, Obujabara	Fruit	Eating, Boiling, Pounding, Tea	Hypertension		[38,176]
*Morus nigra*	Moraceae			Whole	Eat as fresh fruit	Diabetes	Zambia	[177]
*Cucurbita pepo*	Cucurbitaceae			Seeds	Roasting, Pounding			
*Ziziphus abyssinica*	Rhamnaceae			Roots	Maceration			
*Azaza garckeana*	Malvaceae			Roots and fruit	Eating, Maceration			
*Solanum aculeastrum*	Solanaceae			Root	Maceration			
*Eleusine coracana*	Poaceae			Whole				

**Table 2:** Medicinal plants used for the treatment of cardiovascular diseases and their associated risk factors in two or more sub-Saharan African countries.

**Botanical Name**	**Family**	**English/** **Common Name**	**Local Name**	**Plants’ Parts Used**	**Mode of Usage/** **Preparation**	**Diseases** **Treated**	**Countries**	**References**
*Abelmoschus esculentus* (L.) Moench	Malvaceae		Nefu	Fruit, Seed	Decoction, Maceration, Powder	Diabetes	Gabon	[88]
			Okra pods, Bamia	Fruits		Diabetes	Tanzania	[169]
				Whole plant	Maceration	Diabetes	Zambia	[177]
*Abrus precatorius* L.	Fabaceae	Rosary pea	Ojou ega, Azetonounkoun	Leaf	Trituration	Diabetes	Benin	[47]
			Mudepu	Leaf	Decoction	Cardiovascular diseases	Gabon	[86]
				Leaf		Diabetes	Ghana	[178]
			Kyuma Kyamditi	Leaf	Decoction	Diabetes	Kenya	[96]
			Idon zakara	Leaf, Seed	Maceration	Diabetes, Hypertension	Nigeria	[123,128,178]
*Acacia senegal* (L.) Willd	Fabaceae		Gopealega	Stem bark	Decoction	Heart disorders	Burkina Faso	[54]
			Al-Hashab	Fruit, Stem	Powder	Diabetes	Sudan	[156,160]
*Acacia nilotica* (L.) Delile	Fabaceae	Gum Arabica, Egyptian mimosa	Ghered	Seed, Stem bark	Decoction	Diabetes	Eritrea	[72]
				Fruit		Diabetes	Ethiopia	[83]
			Ngiruriti	Bark, Root	Decoction	Diabetes	Kenya	[98]
			Bagaruwa	Root	Decoction	Diabetes	Nigeria	[128]
			Garad	Pod, Bark	Crushing	Diabetes, Hypertension	Sudan	[156,158]
			Olkiroliti	Bark		Diabetes	Tanzania	[166]
*Acalypha wilkesiana*	Euphorbiaceae			Leaf		Diabetes	Mauritius	[131]
			Aworoso	Leaf	Decoction, Eat, Juice,	Diabetes, Hypertension	Nigeria	[124,179]
*Acanthospermum hispidum* DC	Asteraceae		Ichakoro-oro, Godoko	Leaf	Decoction	Diabetes	Benin	[47]
			Ahlangovi	Whole plant	Decoction	Diabetes	Togo	[173]
*Achyranthes aspera* L.	Amaranthaceae			Whole plant	Decoction	Diabetes	Cote d’Ivoire	[70]
			Isinama	Root		Hypertension	South Africa	[99]
*Adansonia digitata* L.	Malvaceae	Baobab	Tweega	Flower	Decoction	Heart disorders	Burkina Faso	[54]
						Diabetes	Cameroon	[59]
			Teydoum	Fruit	Powder	Hypertension	Mauritania	[112]
			Kuka	Bark, Root	Decoction	Diabetes	Nigeria	[128]
*Aframomum melegueta* (Roscoe) K. Schum.	Zingiberaceae	Alligator pepper	Ata, Atakoun	Fruit, Seed	Maceration, Decoction, Powder	Diabetes	Benin	[47]
			Ossame	Seed, Leaf		Hypertension	Nigeria	[123]
*Afzelia africana* Pers.	Fabaceae			Bark, Root		Diabetes	Guinea	[90]
			Welou			Hypertension	Togo	[170]
*Ageratum conyzoides* L.	Asteraceae			Whole plant		Diabetes	Cameroon	[180]
				Whole plant	Decoction	Diabetes	Cote d’Ivoire	[70]
			Kumba djuma	Leaf, Whole plant	Infusion, Juice	Diabetes	Gabon	[88]
			Apaasa	Root		Heart attack	Nigeria	[34]
			Ganagbe	Whole plant	Decoction	Diabetes	Togo	[173]
*Ajuga remota* Benth.	Lamiaceae		Armagusa	Leaf	Decoction, Juice	Hypertension	Ethiopia	[77]
			Wanjiru waRurii	Whole plant, Leaf	Decoction	Diabetes	Kenya	[96]
*Alchornea cordifolia* (Schumach. & Thonn.) Mull.Arg.	Euphorbiaceae	Christmas bush	Sadiodio, Aboe, Kip-togui	Leaf	Decoction	Hypertension	Cameroon	[60]
				Leaf	Decoction	Diabetes, hypertension	Cote d’Ivoire	[181]
			Dumbundzen	Leaf	Decoction	Diabetes	Gabon	[88]
			Agyamaa	Stem	Decoction	Diabetes	Ghana	[35]
			Ebahue, Mbom, Ipa, Eesin, Osokpo, Akowo, Uwanwe, Upia	Leaf, Stem Bark, Seed, Root	Decoction	Diabetes, Hypertension	Nigeria	[123,181]
						Heart disease	Sierra Leone	[139]
			Avovlo	Leaf	Decoction	Diabetes	Togo	[173]
*Allium cepa* L.	Amaryllidaceae	Onion	Mansa, Ayoman		Decoction	Diabetes, Hypertension	Benin	[23,47]
			Sasinsala	Bulb	Powder	Hypertension	Burkina Faso	[54]
			Tigneri, Ayan, Djanga, Agnosi	Bulb	Decoction	Cardiovascular disease, Diabetes, Hypertension	Cameroon	[59,60]
			Intunguru, Matungu, Lintuguru	Bulb	Decoction, Maceration	Diabetes, Hypertension	DR Congo	[23,66,67]
			Shiguerti-keyih	Bulb		Diabetes	Eritrea	[72]
						Hypertension	Ethiopia	[23]
			Nyonda	Bulb		Diabetes	Gabon	[88]
			Zoiyon / Oignon	Bulb	Decoction	Diabetes, High Cholesterol level	Mauritius	[113,182]
			Alubarha, Alubosa obe	Bulb	Juice	Hypertension	Nigeria	[23,34,123,125]
				Green top of sprout, root, bulb	Juice	Diabetes	South Africa	[147]
			Basal	Bulb		Diabetes	Sudan	[156]
			Albassa			Hypertension	Togo	[23,170]
*Allium sativum* L.	Amaryllidaceae	Garlic	Ayo		Direct consumption, Decoction, Maceration, Powder	Diabetes, Hypertension	Benin	[23,47]
			Laye	Bulb	Powder	Hypertension	Burkina Faso	[54]
			Ayang ntangan	Bulb, Rhizome	Decoction, Maceration	Cholesterol problem, Diabetes, Hypertension	Cameroon	[56,59,60]
			Itunguru sumu, Lintugunru sumo	Bulb	Decoction	Diabetes, Hypertension	DR Congo	[23,67]
				Bulb	Tincture, Maceration	Diabetes	Cote d’Ivoire	[70]
			Shiguerti-tsaeda	Bulb	Consumed raw or added in sauce	Diabetes, Hypertension	Eritrea	[23,72]
			Kulubi adi	Bulb	Powder	Diabetes, Hypertension	Ethiopia	[23,77]
			Ail	Bulb, Cloves	Decoction, Maceration	Cardiovascular diseases, Diabetes	Gabon	[86,87,88]
				Leaf, Bulb		Diabetes	Guinea	[90]
			Caumu, Kitunguua kinene	Cloves, Bulb	Decoction, Infusion	Diabetes	Kenya	[96,98]
						Hypertension	Madagascar	[23]
			L’ail	Bulb	Decoction	Diabetes, Hypertension	Mauritius	[23,113,182]
			Ayur, Tafarnuwa, Etebe owoinu	Bulb	Eat Fruit	Diabetes, Hypertension	Nigeria	[23,123,125,128,132]
						Diabetes, Hyperlipidemia, Hypertension, Stroke, Weight reduction	Sierra Leone	[23,138]
				Flower bud		Hypertension	South Africa	[99]
			Toom	Bulb		Diabetes, Hypertension	Sudan	[156,157]
						Hypertension	Tanzania	[23]
			Ayo			Diabetes, Hypertension	Togo	[23,170]
			Katunguluccumu	Bark	Chewing	Reduce heart beat	Uganda	[38]
				Whole	Maceration	Diabetes	Zambia	[177]
*Aloe arborescens* Mill.	Xanthorrhoeaceae	Krantz aloe	Kransaalwyn, Ikalane, Inkalane, Umhlabana	Leaf	Decoction	Diabetes	South Africa	[147]
			Inhlaba lencane	Leaf	Decoction	Diabetes	Swaziland	[161]
*Aloe buettneri* A. Berger	Xanthorrhoeaceae		Chanmanchanman, Aloes	Leaf	Trituration, Maceration, Powder	Diabetes	Benin	[47]
				Whole plant	Decoction, Maceration, Infusion		Cote d’Ivoire	[70]
			Mimi	Leaf	Infusion	Diabetes	Togo	[173]
*Aloe ferox* Mill.	Xanthorrhoeaceae	Bitter aloe, Cape aloe	Lekhala la Quthing	Leaf, Root		Diabetes, Hypertension	Lesotho	[42]
			Ikhala-lasekoloni, Bitter alwyn	Leaf, Sap, Roots	Boiling, Decoction	Obesity, Diabetes, Hypertension	South Africa	[140,143,145,147,149,152]
*Aloe marlothii* A. Berger	Xanthorrhoeaceae	Mountain aloe	Kgopha ya go eema, inhlaba umhlaba, Inhlaba, Bindamutshe, Mhangani	Leaf, Root	Cooking, Decoction	Diabetes, Hypertension	South Africa	[99,141,147,153,183]
			Inhlaba lenkhulu	Leaf		Cardiac problems	Swaziland	[184]
*Aloe striatula* Haw.	Xanthorrhoeaceae		Mohalakane/Seholobe	Leaf		Hypertension	Lesotho	[42]
						Hypertension	South Africa	[99]
*Aloe vera* (L.) Burm.f.	Xanthorrhoeaceae	Aloe, True aloe, Aloe vera Burn aloe, Indian aloe, Barbados aloe				Diabetes	Cameroon	[59]
				Leaf	Maceration	Hypertension	Central African Republic	[64]
			Subiri	Leaf	Maceration	Diabetes	DR Congo	[66]
			Aloe vera	Leaf	Decoction	Diabetes	Ghana	[35]
			Aloe vera	Leaf		Diabetes, High Cholesterol Level	Mauritius	[113]
				Leaf		Hypertension	Nigeria	[123]
				Leaf, Gel	Decoction	Diabetes	South Africa	[147]
			Aloe, Alovera	Gel, Leaf, Rind, Stem	Extract	Diabetes	Tanzania	[165,169]
			Fradjo, Faradjo	Root	Decoction	Diabetes	Togo	[170,171]
				Whole	Maceration	Diabetes	Zambia	[177]
*Alstonia boonei*	Apocynaceae	Stool wood	Emien	Stem bark	Decoction, Maceration	Diabetes	Gabon	[87,88]
			Awun	Root	Decoction	Diabetes	Nigeria	[126]
			Gnamidua	Leaf	Decoction	Diabetes	Togo	[173]
*Amaranthus dubius*	Amaranthaceae		Brede Malbar	Leaf	Cooking, Eating	Hypertension	Mauritius	[116]
				Leaf	Maceration	Hypertension	South Africa	[99]
*Ambrosia maritima* L.	Asteraceae				Decoction	Diabetes	Ethiopia	[83]
			Damsisa, Damesisa	Leaf		Diabetes, Hypertension	Sudan	[156,157]
*Ammi visnaga* (L.) Lam	Apiaceae	Khella, Picktoth	E’bna	Leaf	Extract	Diabetes	Eritrea	[72]
			Bizrat al khalla	Fruit		Diabetes	Sudan	[156]
*Anacardium occidentale* L.	Anacardiaceae	Cashew tree, Cashew, Cashew nut, Cashew nut tree, Cashew apple	Egu acajou, Kajoutin	Bark, Root, Leaf	Decoction, Powder, Maceration	Diabetes	Benin	[47]
				Leaf		Diabetes, Hypertension	Cameroon	[57,59]
				Leaf, Stem bark	Maceration, Infusion, Decoction	Diabetes	Cote d’Ivoire	[70]
				Leaf, Fruit, Bark		Diabetes	Guinea	[90]
			Mahabibo	Leaf, Fruit	Tea	Diabetes	Madagascar	[30,84,107]
			Kasjoeneut	Bark	Tincture	Diabetes	South Africa	[147]
			Atchan, Yovotsan	Leaf	Decoction	Diabetes, Hypertension	Togo	[170,173]
*Ananas comosus* (L.) Merr.	Bromeliaceae		Abrebre, Agonde	Fruit, Root	Decoction	Diabetes	Benin	[47]
						Obesity	Cameroon	[59]
*Anchomanes difformis* (Blume) Engl.	Araceae			Rhizome	Maceration	Diabetes	Cote d’Ivoire	[70]
			Nkwe-ndojgu	Rhizome	Maceration	Diabetes	Gabon	[87]
						Heart attack	Sierra Leone	[139]
*Annona muricata* L.	Annonaceae					Hypertension	Benin	[23]
			Saoussawa awaki, Saba saba, Bwembe, Ebom	Leaf	Boiling, Decoction	Heart pain, Hypertension	Cameroon	[59,60]
				Leaf	Infusion	Diabetes	Cote d’Ivoire	[70]
			Corossolier, Soursop	Leaf, Stem bark	Maceration, Decoction	Cardiovascular diseases, Diabetes	Gabon	[86,87,88]
				Leaf	Tea	Heart palpitations	Madagascar	[107]
			Coronsol	Leaf	Infusion	Hypertension	Mauritius	[23,113,118]
			Soursop	Leaf	Juice	Diabetes	Tanzania	[169]
			Sabissab, Agnigli	Leaf	Infusion	Diabetes	Togo	[170,173]
*Annona senegalensis* Pers.	Annonaceae	African custard-apple	Otrin-bobo, Gnigiwe	Leaf, Root	Decoction	Diabetes	Benin	[47]
			Gwadda-daji	Leaf	Maceration	Diabetes	Nigeria	[128]
			Tchoutchoude			Diabetes, Hypertension	Togo	[170]
*Anogeissus leiocarpa* (DC.) Guill. & Perr.	Combretaceae	Axlewood tree	Agni, Hihon	Bark	Powder	Diabetes	Benin	[47]
				Leaf	Decoction	Diabetes	Cote d’Ivoire	[70]
			Marke	Leaf, Bark	Maceration, Decoction	Diabetes	Nigeria	[128]
			Suhab	Inflorescence	Maceration	Diabetes	Sudan	[158]
			Heheti	Leaf	Decoction	Diabetes	Togo	[173]
*Anthocleista djalonensis* A.Chev.	Gentianaceae		Bheino modyo	Root	Decoction	Diabetes	Guinea	[93]
			Ezenukpogan, Sapo	Leaf, Bark, Root	Decoction, Maceration	Diabetes, Hypertension	Nigeria	[34,93,123,126]
			Assoubo-bissaou, Gboloba	Leaf, Stem bark, Root	Powder, Decoction	Diabetes, Hypertension	Togo	[93,170,171,173]
*Anthocleista vogelii* Planch.	Gentianaceae		Gotun, Guswe	Root	Decoction	Diabetes	Benin	[47]
			Ekoka ngowa	Stem bark, Leaf	Decoction	Diabetes	Cameroon	[56,93]
			Teng mavassa, Ayinebe, Givindu	Leaf, Stem bark, Root	Decoction, Maceration	Cardiovascular diseases, Diabetes	Gabon	[86,88]
			Sapo, Kwari, Awudifo-Akete	Root,	Decoction	Diabetes	Ghana	[93]
			Benin rope, Sapo, Kwari, Awudifo-Akete, Mpoto	Stem, Bark, Root	Decoction, Maceration	Diabetes, Hypertension, Obesity	Nigeria	[93,123]
*Arachis hypogaea* L.	Fabaceae		Sigkaam/Naguri	Leaf	Charred	Heart disorders	Burkina Faso	[54]
			Gertah	Seed	Infusion	Diabetes	Mauritania	[112]
			Amakinati	Leaf, Seed	Decoction, Cooking	Hypertension	South Africa	[99,153]
*Aristolochia albida* Duch.	Aristolochiaceae		Waregou, Fondo	Root	Maceration, Decoction, Infusion	Diabetes	Benin	[47]
			Agadayo [170]			Diabetes	Togo	[170]
*Artemisia absinthium*	Asteraceae	Wormwood, Grand wormwood, Absinthe	Kanyambuba kalume	Leafy stem	Decoction	Diabetes	DR Congo	[67]
			Groenamara	Leaf	Infusion	Diabetes, Hypertension	South Africa	[147,149]
*Artemisia afra*	Asteraceae	African wormwood, Wild wormwood	Mhlonyane, Wilde als, Umhlonyane, Alsbos, Lengana, Zengana	Leaf, Root	Boiling, Decoction, Infusion	Diabetes, Hypertension	South Africa	[53,99,143,147,149]
			Fivi, Majani mapana artemisia	Leaf, Stem, Root		Diabetes	Tanzania	[165,169]
*Artocarpus altilis*	Moraceae		Weeteyangului	Leaf, Root	Boiling, Decoction	Diabetes	Liberia	[185]
			Fruit a pain	Leaf	Decoction	Diabetes	Mauritius	[113]
*Azadirachta indica* A. Juss	Meliaceae	Neem, Indian lilac	Jediya, Kininitin	Bark, Leaf	Decoction, Trituration	Diabetes	Benin	[47]
			Dogoyaro	Seed, Leaf, Bark	Decoction	Diabetes	Cameroon	[56]
				Leaf, Stem bark	Decoction	Diabetes	Cote d’Ivoire	[70]
			Neem	Leaf, Stem bark	Decoction	Diabetes	Eritrea	[72]
			Mwarubaine	Stem bark, Leaf	Decoction	Diabetes	Kenya	[96]
			Nimo	Leaf		Diabetes, High cholesterol	Madagascar	[30]
			Neem, Lila perche	Leaf	Decoction, Juice	Diabetes	Mauritius	[113,116]
			Dogonyaro, Dongoyaro	Leaf, Bark, Root	Decoction, Infusion	Cardiac activities, Diabetes	Nigeria	[126,128,186,187]
			Kiniti	Bark, Root	Decoction	Diabetes	Togo	[173]
*Balanites aegyptiaca* (L.) Delile	Zygophyllaceae	Desert date, Soapberry tree	Kiagelga	Stem Bark	Charred	Heart disorders	Burkina Faso	[54]
			Mekie	Leaf, Fruit	Extract	Diabetes	Eritrea	[72]
			Higleeg, Laloub, Al-Laloub	Fruit, Seed, Leaf	Fruit pulp eaten, Infusion, Maceration	Diabetes, Hypertension	Sudan	[156,157,158,159,160]
			Aduwa	Leaf, Bark, Root	Decoction, Maceration	Diabetes	Nigeria	[128]
*Bambusa vulgaris* Schrad.	Poaceae	Bamboo, Chinabamboo		Leaf	Decoction	Hypertension	Central African Republic	[64]
				Leaf	Decoction	Diabetes	Cote d’Ivoire	[70]
			Pampuro ahaban	Leaf	Decoction	Hypertension	Ghana	[89]
			Opaarun	Leaf	Decoction	Diabetes	Nigeria	[126]
*Bersama abyssinica* Fresen.	Melianthaceae		Azamr	Leaf, Root	Boiling, Crushing	Hypertension	Ethiopia	[188]
			Mukilyulu	Root Bark	Decoction	Diabetes	Kenya	[96]
				Stem bark, Root	Decoction	Diabetes	Mozambique	[120]
*Bidens pilosa* L.	Compositae		Tchiogwenoh, Lelete netseke	Whole plant	Decoction, Infusion	Hypertension	Cameroon	[60]
			Koko l limo, Koko ya limo, Kashisha, Nyassa, Igishokoro	Leaf, Leafy Stem	Decoction	Diabetes	DR Congo	[66,67]
				Whole plant	Decoction	Diabetes	Cote d’Ivoire	[70]
					Decoction	Diabetes	Kenya	[95]
			Tsipolotra	Leaf	Soup	Hypertension	Madagascar	[107,108]
			Lavilbag	Leaf	Decoction	Diabetes, Hypertension	Mauritius	[113]
				Leaf	Maceration	Diabetes	Zambia	[177]
*Blighia sapida* K. D.Koenig	Sapindaceae	Ackee	Egui ishin, Lisetin	Bark, Branches, Leaf	Decoction	Diabetes	Benin	[47]
				Seed, Fruit	Decoction	Diabetes	Cote d’Ivoire	[70]
			Gwanja Kusa, Isin, Okpu	Leaf, Stem bark, Root bark		Diabetes, Hypertension	Nigeria	[189,190]
*Boerhavia diffusa* L.	Nyctaginaceae			Whole plant	Maceration	Diabetes	Cote d’Ivoire	[70]
			Katson agni	Whole plant	Decoction	Diabetes	Togo	[173]
*Brachylaena discolor* DC.	Compositae	Silver oak, Coastal/Natal silver leaf	Umphahla, Bosvaalbos, Vaalbos	Leaf	Boiling, Infusion	Diabetes	South Africa	[53,142,147]
			Mupasa	Leaf	Chewing, Juice	Diabetes	Zimbabwe	[53]
*Brassica oleracea* L.	Brassicaceae			Leaf	Consumed in form of sauce	Diabetes	Benin	[47]
			Shu	Leaf	Decoction	Diabetes	DR Congo	[66]
			Li chou	Leaf	Juice	Cardiovascular diseases, Diabetes	Mauritius	[113]
*Bridelia ferruginea* Benth.	Phyllanthaceae		Ira, Honsoukokoe	Leaf, Bark	Decoction, Powder	Diabetes	Benin	[47]
			Nonro	Root	Boiling, Decoction	Hypertension	Central African Republic	[64]
				Leaf, Root	Decoction	Diabetes	Cote d’Ivoire	[70]
			Kizni	Bark	Decoction	Diabetes	Nigeria	[128]
			Kolou, Akamati	Leaf + Root	Decoction	Diabetes	Togo	[170,173]
*Bulbine narcissifolia*	Xanthorrhoeaceae		Khomo ea balisa	Bulb, Root		Diabetes	Lesotho	[42]
					Decoction	Diabetes	South Africa	[147]
*Caesalpinia bonduc* (L.) Roxb.	Fabaceae	Gray Nicker Bean	Egui adji, Djikouin	Leaf, Root	Infusion, Decoction, Maceration	Diabetes	Benin	[47]
			Okwen	Leaf	Infusion	Hypertension	Nigeria	[123]
			Adiku	Leaf	Decoction	Diabetes	Togo	[173]
*Caesalpinia pulcherrima* (L.) Sw.	Fabaceae		Chrochro, Fleur	Leaf	Decoction	Diabetes	Benin	[47]
				Leaf, Stem	Decoction	Diabetes	Cote d’Ivoire	[70]
*Cajanus cajan* (L.) Millsp.	Fabaceae	Pigeon peas	Olene, Otiili	Leaf	Powder	Diabetes, Hypertension	Nigeria	[123,126]
			Mbainisiri, Majani ya mbaazi	Leaf, Seed	Decoction	Diabetes, Heart diseases	Tanzania	[165,168,169]
*Calotropis procera*	Asclepiadaceae	Sodom apple	Bambamba, Amonman	Leaf, Root	Trituration, Decoction	Diabetes	Benin	[47]
			Putrupuugu	Root	Powder	Heart disorders	Burkina Faso	[54]
			Ghinde’a	Stem bark, Latex	Crushing	Diabetes	Eritrea	[72]
*Cannabis sativa* L.	Cannabaceae	Marijuana, Indian hemp	Igbo, Gueman	Leaf	Infusion	Diabetes	Benin	[47]
			Isangu, Um Ya, Intsangu, Dagga, Nsanga, Insangu	Leaf	Crushing, Soaking, Infusion, Decoction	Diabetes, Hypertension, Obesity, Stroke	South Africa	[7,140,142,147,153]
*Capparis decidua* (Forssk.) Edgew.	Capparaceae	Caper berry	Sorob	Leaf, Stem Bark	Infusion	Diabetes	Eritrea	[72]
			Tundub	Stem		Diabetes	Sudan	[156]
*Capsicum frutescens* L.	Solanaceae	Pepper	Tambo-olobre, Takin winiwini	Fruit	Decoction, Powder	Diabetes	Benin	[47]
			Kambi, Kipiarga	Fruit	Decoction	Heart disorders	Burkina Faso	[54]
				Fruit		Hypertension	Cameroon	[191]
			Esin	Fruit		Hypertension	Nigeria	[123]
*Carica papaya* L.	Caricaceae	Pawpaw	Aguidi ako, Kpintin	Leaf, Root, Fruit	Decoction	Diabetes, Hypertension	Benin	[23,47]
			Pawpe	Whole plant	Decoction	Cardiovasular diseases, Diabetes, Hypertension	Cameroon	[56,59]
			Ipapayi, Papayi	Leaf, Root	Decoction	Diabetes	DR Congo	[67]
			Papayo, Papayio	Fruit, Leaf, Seed	Boiling, Decoction	Diabetes, Hypertension	Eritrea	[23,72,74]
			Papaya, Mulolu	Leaf, Fruit, Pulp, Seed, Root	Decoction, Infusion, Juice	Diabetes	Gabon	[87,88]
			Ahaban	Leaf	Decoction	Hypertension	Ghana	[23,89]
				Leaf, Root		Diabetes	Guinea	[90]
			Papaya	Fruit	Direct Consumption, Juice	Cardiovascular disease, High Cholesterol level, Hypertension	Mauritius	[23,113,182]
			Okodu, Ako-ibepe, Gwadda, Udia edi, popo, ukpod	Seed, Leaf, Fruit, Root	Decoction, Maceration, Tea	Diabetes, Heart attack, Hypertension, Obesity	Nigeria	[23,34,123,125,128,132,187,192]
			Mophopho	Root	Cooking	Diabetes	South Africa	[141,183]
				Root	Decoction	Diabetes	Zambia	[177]
*Carissa edulis*	Apocynaceae	Num-num	Agam	Stem bark	Extract	Diabetes	Eritrea	[72]
			Mukawa	Leaf, Bark, Root	Decoction	Diabetes	Kenya	[98]
*Carissa spinarum* L.	Apocynaceae		Ahanzo	Root	Decoction, Maceration, Powder	Diabetes	Benin	[47]
					Decoction	Diabetes	Kenya	[95]
*Cassia abbreviata* Oliv.	Fabaceae	Long-tail cassia	Monepenepe	Root	Powder	Diabetes	Botswana	[52]
			Malandesi	Leaf, Pod	Decoction	Diabetes	Kenya	[96]
			Molomanama	Stem bark	Extract	Diabetes	South Africa	[147]
			Mkundekunde	Root		Diabetes	Tanzania	[169]
				Root	Maceration	Diabetes	Zambia	[177]
*Cassia alata* L.	Fabaceae	Ringworm bush	Mapalata, Nkaya loto	Leaf	Decoction	Diabetes	DR Congo	[66]
			Kattreping			Hypertension	Mauritius	[114]
			Asuwon	Stem bark	Powder	Diabetes	Nigeria	[126]
*Cassia occidentalis* L.	Fabaceae	Violet tree, Coffee Senna	Nom sas	Seed	Maceration	Hypertension	Cameroon	[60]
						Diabetes	Comoros	[65]
			Mwenga jinni, Ma nsanmzi usambi, Mushegamanjoka, Kashegema, Lukunda bajanyi, Mbaw-mbaw	Leaf, Root, Whole plant	Decoction	Diabetes	DR Congo	[66,67,69]
				Leaf	Decoction	Diabetes	Cote d’Ivoire	[70]
			Itanna-akorere, Sanga-sanga	Seed, Leaf, Root	Decoction, Infusion	Diabetes, Stroke	Nigeria	[34,128]
			Stinky Leaf			Diabetes, Hypertension	Sierra Leone	[138,139]
			Soreib	Seed	Decoction	Hypertension	Sudan	[158]
*Cassia sieberiana* DC.	Fabaceae	Africa Laburnum, West African laburnum	Mugunga, Mununga nunsi	Leaf		Diabetes	DR Congo	[69]
			Sindja	Leaf, Root	Decoction	Diabetes, Hypertension	Guinea	[90,92]
			Aridantoro, Malga	Leaf, Root	Decoction, Maceration	Diabetes	Nigeria	[126,128]
			Gat-Gati	Root	Decoction	Diabetes	Togo	[173]
*Cassia tora* L.	Fabaceae	Foetid senna	Sigdre	Leaf	Charred	Hypertension	Burkina Faso	[54]
				Leaf	Decoction, Juice	Cardiac problems, Hypertension	Nigeria	[50,123]
*Catharanthus roseus* (L.) G.Don	Apocynaceae	Madagascar periwinkle	Bonjour bonsoir, Fleur	Leaf, Root	Decoction	Diabetes, Hypertension	Benin	[23,47]
				Root	Decoction	Hypertension	Cameroon	[60]
			Mauwa, Vinka	Leaf	Decoction	Diabetes	DR Congo	[66,67]
				Leaf	Decoction	Diabetes	Cote d’Ivoire	[70]
				Leaf		Diabetes	Guinea	[90]
			Saponnaire	Leaf	Infusion	Diabetes	Mauritius	[113]
				Leaf, Whole plant	Decoction	Diabetes	Mozambique	[53,84]
						Hypertension	Nigeria	[23]
				Seed, Leaf, Flower, Root	Decoction, Infusion	Diabetes, Hypertension	South Africa	[23,99,142,147,153]
						Hypertension	Tanzania	[23]
			Kilakou-sindazi, Coucouvigbe	Leaf, Root	Decoction	Diabetes, Hypertension	Togo	[23,170,173]
*Ceiba pentandra* (L.) Gaertn.	Malvaceae	Boma stick	Bouma, Riwoun, Doum, Heum, Awoueng	Bark, Leave, Root	Decoction, Maceration	Diabetes, Heart palpitations, Hypertension	Cameroon	[56,57,60]
				Leaf	Decoction	Diabetes	Cote d’Ivoire	[70]
			Fromage	Leaf, Stem bark, Bark, Root	Decoction	Diabetes	Gabon	[87,88]
			Araba, Apabida	Bark, Leaf		Hypertension	Nigeria	[34]
*Centella asiatica* (L.)	Apiaceae			Root	Cooking	Diabetes	South Africa	[141]
				Leaf		Diabetes	Tanzania	[169]
*Chromolaena odorata* L.	Asteraceae		Agatou	Root	Decoction	Diabetes	Benin	[47]
				Leaf, Root	Decoction	Diabetes	Cote d’Ivoire	[70]
*Cinnamomum camphora* (L.) J.Presl	Lauraceae		Ravitsara	Leaf		Hypertension	Madagascar	[30]
			Uroselina	Gum		Hypertension	South Africa	[99]
*Cissampelos mucronata* A. Rich.	Menispermaceae		Enyati, Onyati	Hypogeous organs		Hypertension	Angola	[44]
			Tingwet	Root	Boiling	Hypertension	Kenya	[97,175]
			Leginiko/Jenkolo	Root		Stroke	Nigeria	[34]
*Cissampelos pareira* L.	Menispermaceae			Leaf, Root		Diabetes	Kenya	[150]
			Godogbo	Stem, bark, roots		Hypertension	Nigeria	[34]
*Citrullus colocynthis* (L.) Schrad.	Cucurbitaceae		Kaka chaimkpe, Goussi	Endocarp	Maceration	Diabetes	Benin	[47]
			Hdajlehmar	Seed of fruit		Diabetes	Mauritania	[112]
			Handal, Hundal, Al-Handal	Seed, Fruit, Stem, Root	Poultice	Diabetes	Sudan	[155,156,157,160]
*Citrus aurantiifolia* (Christm.) Swingle	Rutaceae	Lime	Jogorotan ogodo, Kletin	Leaf, Fruit, Jus, Root	Decoction, Powder, Maceration, Juice	Diabetes, Hypertension	Benin	[23,47]
				Leaf		Diabetes	Chad	[193]
						Hypertension	Congo	[23]
				Leaf	Decoction	Diabetes	Cote d’Ivoire	[70]
			Lomi	Leaf		Hypertension	Ethiopia	[23,80]
			Limon	Fruit	Juice	Diabetes, Hypertension, Cardiovascular disease	Mauritius	[23,113]
			Agbopi	Fruit		Hypertension	Nigeria	[23,123]
			Akanka			Hypertension	Togo	[23,170]
*Citrus limon* (L.) Osbeck	Rutaceae	Lemon				Hypertension	Cameroon	[59]
				Leaf		Diabetes	Chad	[193]
			Mti wa ndimu kali, Ndimu, Chunghwa kali	Fruit	Juice, Extraction	Diabetes	DR Congo	[66,67]
			Limon			Hypertension	Mauritius	[114]
			Osanganyin	Fruit		Diabetes	Nigeria	[126]
			Ulamula	Peel of Fruit, Pulp, Root	Decoction	Hypertension	South Africa	[99,153]
			Nimawa	Fruit	Juice	Hypertension	Uganda	[38]
				Root	Decoction	Diabetes	Zambia	[177]
*Citrus maxima* (Burm.) Merr.	Rutaceae		Pamplemousse	Fruit Peel	Decoction	Diabetes, High Cholesterol Level	Mauritius	[113]
			Upapamuzi	Fruit	Eaten Raw	Hypertension	South Africa	[99,153]
*Clausena anisata* (Willd) Hook.f. ex Benth.	Rutaceae	Horsewood, Maggot killer			Decoction	Diabetes	Ghana	[194]
			Chesamishiet	Leaf, Root	Boiling	Stroke	Kenya	[97]
			Umnukambhiba	Leaf, Root	Maceration, Decoction	Hypertension	South Africa	[99]
			Umnukelambiba	Stem bark	Concoction	Cardiac problems	Swaziland	[184]
			Mjafari	Leaf, Stem, Root	Extract	Diabetes	Tanzania	[165,169]
			Eyra	Leaf	Decoction	Diabetes	Togo	[173]
			Muvengahonye	Leaf		Diabetes	Zimbabwe	[195]
*Cleistopholis patens* (Benth.) Engl. & Diels	Annonaceae			Leaf	Trituration, Kneading	Diabetes	Cote d’Ivoire	[70]
			Doundzou, Ovoc	Stem bark	Decoction	Cardiovascular Diseases	Gabon	[86]
*Clerodendrum myricoides* (Hochst.) R.Br. ex Vatke	Lamiaceae	Ugandense	Sur-betri	Stem bark, Leaf	Extract	Diabetes	Eritrea	[72]
			Muvweia, Munguya	Leaf	Decoction	Diabetes	Kenya	[96]
*Clutia natalensis* Bernh.	Peraceae		Mosali-mofubelu	Root		Diabetes	Lesotho	[42]
				Root	Decoction	Diabetes	South Africa	[147]
*Cocos nucifera* L.	Arecaceae	Coconut	Egui agonkin, Agonkintin	Leaf, Root, Bark, Coco water	Decoction, Maceration	Diabetes	Benin	[47]
			Coconut, Coco	Fiber	Decoction	Diabetes	Gabon	[87,88]
			Coco, Cocotier	Fruit, Oil	Decoction	Diabetes	Mauritius	[113,118]
			Iri-oibo, Agbon	Stem, Stem bark	Infusion	Diabetes, Hypertension	Nigeria	[123,126]
			Kpakpadire			Hypertension	Togo	[170]
*Coffea mauritiana* Lam.	Rubiaceae			Leaf	Decoction, Infusion	Diabetes	Cote d’Ivoire	[70]
			Café marron			Diabetes	Mauritius	[117]
*Cola acuminata*	Sterculiaceae		Obi abata,	Fruit	Decoction, Maceration	Diabetes	Benin	[47]
			Colatier	Fruit	Maceration	Cardiovascular diseases	Gabon	[86]
*Cola nitida* (Vent.)	Sterculiaceae	Kola nut	Goro, Golo	Fruit	Decoction, Maceration	Diabetes	Benin	[47]
						Diabetes	Nigeria	[196]
			Goro			Diabetes	Togo	[170]
*Combretum micranthum*	Combretaceae		Kinkeliba	Leaf	Infusion	Diabetes	Gabon	[87,88]
			Kankaliba	Leaf	Decoction	Diabetes, Hypertension	Guinea	[90,92]
*Commiphora africana* (A. Rich.)	Burseraceae	Hairy corkwood, Namibian corkwood	Harige Kanniedood	Bark	Decoction	Diabetes	South Africa	[147]
			Muntonto, Esilalei	Roots, bark		Diabetes	Tanzania	[166]
*Corchorus olitorius* L.	Malvaceae		Yoyo, Ninnounwi	Seed	Powder	Diabetes	Benin	[47,197]
			Ewedu, Ahuhara	Leaf twigs, Leaf, Seed	Powder	Diabetes, Heart troubles	Nigeria	[124,197]
			Ademe	Leaf	Powder	Diabetes	Togo	[173]
*Crossopteryx febrifuga* (Afzel. ex G.Don) Benth.	Rubiaceae		Kum-waga	Fruit	Maceration	Obesity	Burkina Faso	[54]
			Musanzambeke, Nakasabuni	Root, Bark		Diabetes, Hypertension	Tanzania	[166,169]
*Croton macrostachyus* Del.	Euphorbiaceae		Mutundu, Kitundu	Root bark		Diabetes	Kenya	[96]
			Mhulugu, mbiha			Diabetes	Tanzania	[169]
*Croton zambesicus*	Euphorbiaceae		Animonegiee	Fruit		Hypertension	Nigeria	[123]
			Um gleigla	Seed, Fruit		Hypertension	Sudan	[198]
*Curculigo pilosa*	Hypoxidaceae		Ikoule-gouchou, Ayote	Tuber	Decoction	Diabetes	Benin	[47]
			Epaikun	Root		Hypertension	Nigeria	[34]
*Cymbopogon citratus* (DC.) Stapf	Poaceae		Ofrin, Cha/Timan	Leaf	Decoction	Diabetes, Hypertension	Benin	[23,47]
			Djenji, Ossanga, Fiba glass	Root	Decoction	Hypertension	Cameroon	[60]
			Majani tshai	Leaf	Decoction	Diabetes, Hypertension	DR Congo	[23,66]
				Rhizome	Decoction	Diabetes	Cote d’Ivoire	[70]
			Citronelle, Lemongrass, Ditsotsu	Leaf	Decoction, Infusion	Cardiovascular diseases, Diabetes	Gabon	[86,87,88]
						Hypertension	Nigeria	[23]
			Mchaichai	Leaf, Stem		Diabetes	Tanzania	[169]
			Tigbe			Diabetes	Togo	[170]
*Dalbergia melanoxylon* Guill. & Perr.	Fabaceae		Abanous, Al-Babanous	Leaf	Infusion	Heart pain	Sudan	[157,160]
			Mpingo	Leaf	Juice	Cardiac trouble	Tanzania	[168]
*Daniellia oliveri* (Rolfe) Hutch. & Dalziel	Fabaceae	African copaiba balsam	Iya, Zantin, Wuya, Yanburu	Leaf, Bark, Root	Decoction, Maceration	Diabetes, Obesity, Heart attacks in children	Benin	[47,199]
						Hypertension	Mali	[200]
			Majee	Bark	Decoction	Diabetes	Nigeria	[128]
*Daucus carota* L.	Apiaceae	Carrot		Root	Consumed in form of sauce	Diabetes	Benin	[47]
				Tuber	Expression, Grating, Juice	Diabetes	Cote d’Ivoire	[70]
			Caroti	Tuber	Eaten raw or with salad	Diabetes	Eritrea	[72]
*Detarium microcarpum* Guill. & Perr.	Fabaceae	Tallow tree	Ajekofole-oko, Dakpa	Bark, Root	Decoction	Diabetes	Benin	[47]
			Arira	Stem bark	Infusion	Diabetes	Nigeria	[126]
*Dialium guineense* Willd.	Fabaceae		Meeko	Leaf	Decoction	Hypertension	Guinea	[92]
			Uge	Fruit		Hypertension	Nigeria	[123]
*Dichrostachys cinerea* (L.) Wight & Arn.	Fabaceae		Susutri	Leaf	Juice	Heart disorders	Burkina Faso	[54]
			Mkalala	Root		Diabetes	Tanzania	[169]
			Bouvoum			Diabetes	Togo	[170]
*Dicoma anomala*	Asteraceae	Fever bush, Stomach bush	Hloenya	Leaf, Root		Diabetes, Hypertension	Lesotho	[42]
			Umuna, Hloenya,	Leaf, Root	Decoction	Diabetes, Hypertension	South Africa	[99,147]
*Diospyros mespiliformis* Hochst.	Ebenaceae		Gaaka	Fruit	Charred	Heart insufficiency	Burkina Faso	[54]
				Leaf, Stem	Decoction	Diabetes	Cote d’Ivoire	[70]
			Tigbado			Hypertension	Togo	[170]
*Elaeis guineensis Jacq.*	Arecaceae	Palm oil	Egui ekpe, Detin	Branch, Root	Decoction, Maceration	Diabetes	Benin	[47]
			Mbourou	Leaf	Boiling, Decoction	Hypertension	Central African Republic	[64]
				Fruit (Palm oil)		Palpitation, Heart trouble	Liberia	[201]
*Elephantorrhiza elephantina* (Burch.) Skeels	Fabaceae	Eland’s bean, Aland’s wattle, Elephant’s root	Mositsane	Rhizome		Heart conditions, Hypertension	Lesotho	[42]
			Intolwane, Igwejobmvu, Mupangara, Mositsane	Leaf, Rhizome	Decoction, Infusion	Diabetes, Hypertension	South Africa	[7,99,147]
*Entada abyssinica* A. Rich.	Fabaceae	Tree entanda	Halke	Leaf, Stem bark	Decoction	Diabetes	Eritrea	[72]
			Mufutwambula, Mfutwamvula, Ngemwambula	Leaf, Bark, Root	Soaking or boiling in water	Diabetes, Hypertension	Tanzania	[166,169]
*Entada gigas* (L.) Fawc. & Rendle	Fabaceae			Leaf, Stem	Decoction	Diabetes	Cote d’Ivoire	[70]
			Coeur de mer	Stem bark	Decoction	Diabetes	Gabon	[87,88]
*Erythrina abyssinica* DC.	Fabaceae		Kinsungu, Isungwa, Katshiyitshiyi	Bark, Root	Decoction	Diabetes	DR Congo	[69]
			Mkalwanhuba	Root	Powder	Diabetes	Tanzania	[169]
				Bark	Decoction	Diabetes	Zambia	[177]
*Eucalyptus globulus* Labill.	Myrtaceae	Eucalyptus	Tsaeda-kelamintos, Ts-Kelamitoes	Leaf, Bark	Extract, Crushing, Boiling	Diabetes	Eritrea	[72,74]
			Eucalyptus	Leaf	Infusion	Diabetes	Mauritius	[113]
			Ronde bloekomblaar		Infusion	Diabetes	South Africa	[147]
			El kafour	Leaf		Diabetes	Sudan	[156]
*Euclea natalensis A.* DC	Ebenaceae	Large-leaved guarri	Mukinyei	Root	Decoction	Diabetes	Kenya	[96,202]
			Mohlakola, Natal guarri	Bark, Root	Boiling, Decoction	Diabetes	South Africa	[145,147,202]
			Mdala	Root Bark		Diabetes	Tanzania	[169]
*Euphorbia clavarioides* Boiss.	Euphorbiaceae	Lions spoor	Sehlooko	Whole plant		Diabetes, Hypertension	Lesotho	[42]
			Sehlehle, Sehloko	Whole plant	Decoction	Diabetes	South Africa	[147]
*Euphorbia hirta* L.	Euphorbiaceae	Asthma weed	Ignankoun ayira, Nonsinwe	Whole plant	Powder	Diabetes	Benin	[47]
			Ewuda manyongo	Whole plant	Decoction	Diabetes	Cameroon	[56]
						Hypertension	Comoros	[65]
			Ambeningo	Leaf, Whole plant	Decoction, Maceration	Diabetes	Gabon	[88]
				Leaf		Diabetes	Guinea	[90]
			Jean Robert	Leaf	Decoction	Diabetes	Mauritius	[113]
			Asin uloko	Leaf	Concoction	Hypertension	Nigeria	[123,125]
			Lonzwe, Vakikulu, Mziwaziwa	Leaf, Bark, Root	Decoction	Diabetes, Hypertension	Tanzania	[166,169]
			Notsigbe, Anosigbe, Anossika	Whole plant	Decoction	Diabetes, Hypertension	Togo	[172,173]
*Ficus capensis* Thunb.	Moraceae	Sycamore tree		Leaf, Bark, Root		Diabetes	Guinea	[90]
						Hypertension	Mali	[200]
			Opoto	Leaf	Decoction	Diabetes	Nigeria	[126]
*Ficus exasperata* Vahl	Moraceae	Sand paper tree	Lukenga	Leaf	Decoction	Diabetes	DR Congo	[66]
			Borai	Leaf, bark, Root	Decoction, Maceration	Cardiovascular dysfunctions, Diabetes, Heart diseases, Hypertension	Nigeria	[128,203]
*Ficus natalensis* Hochst.	Moraceae		Muuomo/Kiumo	Fruit	Infusion	Diabetes	Kenya	[96]
			Omutoma	Leaf	Decoction	Heart disease	Uganda	[176]
*Ficus platyphylla* Delile	Moraceae	Red robber tree	Agbede, Kapite	Bark	Powder	Diabetes	Benin	[47]
				Stem bark	Decoction	Diabetes	Cote d’Ivoire	[70]
			Gamji	Bark	Decoction	Diabetes	Nigeria	[128]
*Flueggea virosa*	Euphorbiaceae		Wadjedje, Chake-chake	Root, Leaf	Decoction, Maceration	Diabetes	Benin	[47]
				Root	Decoction	Diabetes	Cote d’Ivoire	[70]
			Tchacatchaca	Stem bark	Decoction	Diabetes	Togo	[171]
*Galinsoga parviflora* Cav.	Compositae		Mung’ei	Leaf, Root	Decoction	Diabetes	Kenya	[98]
				Leaf	Maceration	Hypertension	South Africa	[99]
*Garcinia buchananii* Baker	Clusiaceae		Mukanga	Stem bark	Decoction	Diabetes	Kenya	[96]
			Ensali, Nsaala, Musali	Leaf, Bark, Root	Decoction, Pounding	Diabetes, Hypertension, Cardiovascular condition	Uganda	[38,174]
*Garcinia kola* Heckel	Clusiaceae	Bitter kola	Iwo, Ahowe	Fruit	Direct Consumption, Decoction, Maceration, Powder	Diabetes	Benin	[47]
				Fruit		Diabetes	Guinea	[90]
			Edun	Fruit		Hypertension	Nigeria	[123]
*Gardenia ternifolia* Schum.	Rubiaceae			Fruits, Bark, Seed	Decoction, Crushing	Diabetes	Angola	[43]
			Iheung	Bark	Maceration	Hypertension	Cameroon	[60]
				Leaf	Decoction	Diabetes	Cote d’Ivoire	[70]
			Kilindilamugunda	Root		Hypertension	Tanzania	[166]
			Kawou, Flife	Leaf	Decoction	Diabetes, Hypertension	Togo	[170,173]
*Glycine max* (L.) Merr.	Fabaceae	Soybean	Soja	Seed	Powder	Diabetes	Benin	[47]
			Mobe olo	Leaf		Hypertension	Nigeria	[123]
*Gmelina arborea*	Verbenaceae	Beechwood	Milina	Leaf, Bark	Decoction	Hypertension	Nigeria	[123]
			Ansara-tantouna			Hypertension	Togo	[170]
*Graptophyllum pictum* (L.)	Acanthaceae			Leaf	Decoction	Diabetes	Cote d’Ivoire	[70]
			Lait de vierge	Leaf	Decoction	Diabetes	Mauritius	[113,118]
*Gnetum africanum* Welw.	Gnetaceae		Fumbwa	Leaf	Decoction	Diabetes	DR Congo	[66]
			Nkumu	Leaf	Cooking	Diabetes	Gabon	[87,88]
			Afang	Leaf	Chewing, Cooking as soup	Hypertension	Nigeria	[132]
*Guiera senegalensis* J.F.Gmel.	Combretaceae	Moshi medicine	Puglum	Leaf	Charred, Decoction	Hypertension, Heart diseases	Burkina Faso	[54,204]
				Root, Bark	Powder	Hypertension	Cameroon	[56]
			Bufunuk, Kankanango	Leaf		Obesity	The Gambia	[205]
			Sabara	Leaf, Root	Decoction, Maceration	Diabetes	Nigeria	[128,204]
			Gubeish, Gubaish, Ghubeish	Leaf, Root	Decoction, Infusion	Diabetes, Hypertension	Sudan	[155,156,157,158,159,204]
*Gymnema sylvestre* (Retz.) R.Br. ex Sm.	Apocynaceae	Gymnema, Australian cow plant, Periploka of the woods	Shankuk	Leaf	Extract	Diabetes	Eritrea	[72]
					Decoction	Diabetes	Ghana	[194]
				Leaf	Decoction	Diabetes	South Africa	[147]
*Gymnosporia senegalensis* (Lam.) Loes.	Celastraceae		Frefre-ira, Jadoman	Root	Decoction	Diabetes	Benin	[47]
			Aych	Leaf	Infusion	Diabetes	Mauritania	[112]
*Harpagophytum procumbens* (Burch.)	Pedaliaceae	Devils claw	Sengaparile	Tuber	Soaking	Hypertension	Botswana	[52]
			Devils claw	Tuberous root	Infusion, Decoction, Tincture, Powder, Extract	Diabetes	South Africa	[152,206]
*Harrisonia abyssinica*	Simaroubaceae			Leaf	Decoction	Diabetes	Cote d’Ivoire	[70]
			Hedza	Leaf	Decoction	Diabetes	Togo	[173]
*Harungana madagascariensis* Lam. ex Poir.	Hypericaceae		Aton-dog	Bark	Decoction	Hypertension	Cameroon	[60]
			Kadwamuko, Ndura, Ndimu, Chunghwa	Leaf	Decoction, Extraction	Diabetes	DR Congo	[67]
				Stem bark	Decoction	Diabetes	Cote d’Ivoire	[70]
			Atsui	Leaf	Chewing	Diabetes	Gabon	[87,88]
			Soungala		Infusion	Hypertension	Guinea	[92]
			Harongana	Bud Leaf		Heart disease	Madagascar	[102]
*Heliotropium indicum* L.	Boraginaceae	Indian heliotrope	Ogbole arouko, Koklossou dinpaja	Leaf	Decoction	Diabetes	Benin	[47]
			Atapari Obuko	Leaf	Decoction	Diabetes	Nigeria	[126]
*Hibiscus sabdariffa* L.	Malvaceae	Roselle	Bukulu	Calix	Decoction, Infusion, Maceration	Diabetes	Gabon	[88]
			Bissabe		Decoction	Hypertension	Guinea	[92]
			Roselle	Fruit	Juice	Diabetes	Mauritius	[113]
				Flower		Hypertension	Nigeria	[123]
			Karkadeh, Karkady	Calyx	Decoction, Infusion	Hypertension	Sudan	[158,159]
*Holarrhena floribunda*	Apocynaceae			Leaf, Stem Bark	Decoction	Diabetes	Cote d’Ivoire	[70]
			Sesewou	Bark	Decoction	Diabetes	Togo	[173]
*Hordeum vulgare* L.	Poaceae		Gebs		Fermented drink	Hypertension	Ethiopia	[82]
			Sha-yr	Seed	Powder	Diabetes	Mauritania	[112]
*Hoslundia opposita* Vahl	Lamiaceae			Leaf	Decoction	Diabetes	Cote d’Ivoire	[70]
			Omunyenyete	Leaf	Soaking	Hypertension	Tanzania	[162]
*Hymenocardia acida* Tul.	Phyllanthaceae		Oroukpa, Sotive	Root	Maceration	Diabetes	Benin	[47]
			Pellitoro	Leaf	Decoction	Hypertension	Guinea	[92]
				Root	Maceration	Diabetes	Zambia	[177]
*Hyphaene thebaica* (L.) Mart.	Arecaceae	Doum palm	Goriba	Fruit	Eating	Hypertension	Cameroon	[56]
			Goriba	Root	Decoction	Diabetes	Nigeria	[128]
			Dom, Nabag	Fruit, Epicarp	Infusion	Diabetes	Sudan	[156,159]
*Hypoxis hemerocallidea*	Hypoxidaceae	African potato, Yellow star, Star lily, Star flower				Diabetes, Hypertension	Botswana	[207]
			INonqwe, African potato, Ilabatheka, Inkomfe, Sterretjie, Starflowers, Inongwe	Corm, root	Crushing, Decoction	Diabetes, Heart weakness, Hypertension, Stroke	South Africa	[7,53,99,147,152,153]
*Imperata cylindrical* (L.) Raeusch	Poaceae			Rhizome	Decoction	Diabetes	Angola	[43]
			Iguan, Se	Root, Leaf	Decoction/Maceration	Diabetes	Benin	[47]
			Tenona	Leaf		Hypertension	Madagascar	[103]
*Indigofera arrecta*	Fabaceae		Kasholoza, Kavunanfuka, Abwebwe, Musholotsi, Umwikokori, Umusoro	Roots	Chewing	Diabetes	DR Congo	[67]
						Diabetes	Ghana	[55]
*Ipomoea batatas* (L.) Lam.	Convolvulaceae			Leaf	Decoction	Diabetes	Cote d’Ivoire	[70]
			Mongu	Leaf, Tubers, Whole plant	Juice extract, Infusion, Powder	Diabetes	Gabon	[88]
*Irvingia gabonensis*	Irvingiaceae			Bark, fruits, Leaf, roots		Diabetes	Cameroon	[57]
			Mwiba, Africa mango, ndoc	Seed, Fruit, Leaf, Stem bark, Root	Decoction, Maceration	Diabetes	Gabon	[88]
*Isoberlinia doka* Craib & Stapf	Fabaceae		Kpakpa	Leaf	Decoction	Diabetes	Benin	[47]
				Root		Heart disease	Chad	[193]
*Jatropha curcas* L.	Euphorbiaceae	Barbados nut	Okpokporou founfoun, Kpotin wewe	Leaf, Root	Decoction, Triturating, Decoction	Diabetes	Benin	[47]
			Beadounde, Len	Root	Decoction	Hypertension	Cameroon	[60,208]
			Asangi, Mpuluka, Ndolu	Leaf, Bark, Root bark	Decoction	Diabetes, Cardiac palpitation	DR Congo	[66,209]
				Leaf, Stem	Maceration	Diabetes	Cote d’Ivoire	[70]
			Puluka	Seed, Leaf, Root, Stem Bark, Whole plant	Decoction, Maceration	Diabetes	Gabon	[88]
				Leaf, Stem		Diabetes	Guinea	[90]
			Cini dazugu	Leaf, Fruit, Root, Bark	Decoction, Maceration, Fruit burning to ashes	Diabetes	Nigeria	[128,208]
			Babati, Babatihe	Leaf + Root	Decoction	Diabetes, Hypertension	Togo	[172,173]
*Jatropha gossypiifolia* L.	Euphorbiaceae		Okpokporu kpikpa, Kpotin vovo	Leaf	Decoction, Triturating	Diabetes	Benin	[47]
			Babatidzin	Leaf		Diabetes	Togo	[173]
*Kalanchoe crenata* (Andrews) Haw.	Crassulaceae			Whole plant		Diabetes	Cameroon	[57]
			Aflatogan	Root	Decoction	Diabetes	Togo	[173]
*Khaya senegalensis* (Desv.) A.Juss.	Meliaceae	Mahogany, African mahogany	Esisa, Akao, Aganwo, Kayi, Gbira	Leaf, Bark, Root	Decoction, Maceration, Powder, Decoction	Diabetes, Hypertension	Benin	[47,199]
			Kuka	Leaf	Powder	Heart disorders, Obesity	Burkina Faso	[54]
			Okpe, Madacci	Stem bark, Bark	Decoction	Diabetes, Hypertension	Nigeria	[123,128,210]
			Mahogany	Stem bark	Decoction, Maceration	Diabetes	Sudan	[156,160]
			Frimou	Root, Stem bark	Powder, Decoction	Diabetes, Hypertension	Togo	[39,171,210]
*Kigelia africana* (Lam.) Benth	Bignoniaceae	Sausage tree	Gnankpo, Gnanblikpotin	Root, Fruit	Decoction	Diabetes	Benin	[47]
			Mederba, Zelzale	Fruit	Eaten	Diabetes	Eritrea	[72]
				Bark, Seed, Root	Decoction	Diabetes	Kenya	[100]
			Pandoro	Seed		Hypertension	Nigeria	[34]
				Seed	Roasting, Eaten, Crushing	Diabetes	South Africa	[211]
			Um Shutour, Um-Shitour	Fruit	Paste, Decoction	Diabetes, Hypertension	Sudan	[156,160]
			Mudungwa, Mwicha, Mwegea	Root, Bark, Fruit		Diabetes, Hypertension	Tanzania	[166,169]
			Mussa	Leaf, Bark	Ashes	Hypertension	Uganda	[38,211]
				Fruit	Maceration	Diabetes	Zambia	[177]
*Lannea acida* A.Rich.	Anacardiaceae		Assoguidoka, Zonzon	Bark	Decoction	Diabetes	Benin	[47]
				Leaf		Diabetes	Guinea	[90]
*Lantana camara* L.	Verbenaceae		Hlaciayo	Root	Decoction	Diabetes	Benin	[47]
				Leaf, Root	Decoction	Diabetes	Cote d’Ivoire	[70]
			Lantanier, Filawa	Leaf, Root	Decoction, Infusion	Diabetes	Gabon	[87,88]
			Ponpontiaani	Leaf	Infusion	Hypertension	Guinea	[92]
			Radriaka	Leaf	Infusion	Hypertension	Madagascar	[30,108]
			Ubukhwebezane	Root	Decoction, Infusion	Hypertension	South Africa	[99]
			Fonyvi	Leaf	Decoction	Diabetes	Togo	[173]
*Launaea cornuta* (Hochst. ex Oliv. & Hiern) C.Jeffrey	Compositae		Muthunga	Leaf	Chewing, Boiling	Diabetes	Kenya	[98]
			Vanshaqaar, Qaraariye	Leaf, Root, Whole plant	Decoction	Diabetes	Somalia	[212]
*Leptadenia hastata* Vatke	Apocynaceae		Lelongo	Stem Bark	Decoction	Diabetes	Burkina Faso	[54]
				Root	Decoction	Diabetes	Cameroon	[56]
			Yaadiya	Leaf	Decoction	Diabetes, Hypertension	Ghana	[35]
*Leucosidea sericea*	Rosaceae		Cheche	Leaf, Stem		Hypertension	Lesotho	[42]
			Umtshitshi			Hypertension	South Africa	[99]
*Lippia multiflora*	Verbenaceae				Infusion	Hypertension	Ghana	[194]
			Boufazaou				Togo	[170]
*Lophira lanceolata* Tiegh. ex Keay	Ochnaceae		Ngokole	Leaf	Decoction	Hypertension	Central African Republic	[64]
			Kparakpara	Root	Decoction, Powder	Hypertension	Togo	[170]
*Mangifera indica* L.	Anacardiaceae	Mango	Mango, Mangatin	Leaf, Bark, Root	Decoction	Diabetes	Benin	[47]
						Diabetes	Cameroon	[59]
			Mti wa hembe, Nzete ya manga, Mwembe, Hembe, Mongo, Muitie Mamanganga, Mutshi wa mangaya	Root, Stem Bark	Decoction	Diabetes	DR Congo	[66,67,68]
			Mangus	Leaf, Stem Bark	Decoction	Diabetes	Eritrea	[72]
			Mwiba mutangani	Leaf, Seed, Stem-Bark, Root	Decoction	Diabetes	Gabon	[88]
			Mango dua	Stem Bark	Decoction	Hypertension, Diabetes	Ghana	[89]
			Mango Seny	Leaf	Decoction	Hypertension	Guinea	[92]
			Muembe	Leaf	Infusion	Diabetes	Kenya	[98]
			Mangue, Manguier	Leaf	Infusion	Diabetes	Mauritius	[113,118]
			Ebe mango, Mangoro, Mangwaro	Leaf, Stem bark	Maceration, Decoction, Powder	Diabetes, Hypertension	Nigeria	[123,126,128,132]
			Mangoti	Leaf	Decoction	Diabetes	Togo	[173]
				Leaf	Maceration	Diabetes	Zambia	[177]
			Mumango	Bark	Extraction	Diabetes	Zimbabwe	[53]
*Manihot esculenta* Crantz.	Euphorbiaceae		Adjagoun, Fingnignin	Tuber	Maceration	Diabetes	Benin	[47]
			Zengue, Kediann, Makwamba, Cassinga, Mbon	Leaf	Paste, Maceration	Hypertension	Cameroon	[60]
			Mangahazo	Leaf	Infusion	Hypertension	Madagascar	[108]
			Manioc	Leaf	Infusion	Hypertension	Mauritius	[113,118]
*Maytenus senegalensis* (Lam.) Exell	Celastraceae		Tokvugri	Leaf	Decoction	Hypertension	Burkina Faso	[54]
			Tchindjinya			Diabetes	Togo	[170]
*Melanthera scandens*	Asteraceae		Ayara edemerong	Leaf		Diabetes	Nigeria	[213]
			Byabarwoya, Eshurwa	Leaf	Decoction	Diabetes	Tanzania	[169]
*Mentha aquatica* L.	Lamiaceae			Leaf, Stem, Seed	Infusion	Hypertension	South Africa	[99]
			Ehohwa	Leaf	Tea	Hypertension	Uganda	[176]
*Milicia excelsa* (Welw.)	Moraceae		Iroko, Lokotin	Bark	Powder, Maceration	Diabetes	Benin	[47]
			Obiga	Stem bark	Decoction	Diabetes	Gabon	[87,88]
*Mimosa pudica* L.	Fabaceae	Sensitive plant	Bodji	Leaf	Decoction	Diabetes	Gabon	[87,88]
			Ebe-abhurior	Root		Hypertension	Nigeria	[123]
			Sensitive mimosa			Stroke	Sierra Leone	[138]
*Mitragyna inermis* (Willd.) Kuntze	Rubiaceae		Yilga	Leaf	Decoction	Heart disorders	Burkina Faso	[54]
				Leaf, Stem bark	Decoction	Diabetes	Cote d’Ivoire	[70]
			Um Gatto	Fruit		Diabetes	Sudan	[156]
			Limpkati	Leaf	Decoction	Diabetes	Togo	[173]
*Momordica balsamina* L.	Cucurbitaceae	African pumpkin, African cucumber, Bitter gourd, Balsam pear				Cardiovascular disorders, Diabetes	Botswana	[207]
			Garahuni	Leaf	Maceration	Diabetes	Nigeria	[128]
			Mothwatwa, Intshungu, Umkaka	Leaf, Root, Fruit, Seed	Cooking, Decoction, Infusion, Poultice, Tea	Diabetes, Hypertension	South Africa	[99,141,142,146,147,153]
			Ira-ira, Abu el Efain	Leaf, Seed	Infusion	Diabetes	Sudan	[156,158]
			Inkakha	Aerial part	Eaten as a vegetable	Diabetes, Hypertension	Swaziland	[184]
*Momordica charantia* L.	Cucurbitaceae		Kpalari, Yinsikin	Leaf	Decoction, Trituration	Diabetes	Benin	[47]
				Fruit, Leaf, Stem, Whole plant	Decoction	Diabetes	Cote d’Ivoire	[70]
				Leaf	Decoction	Cardiovascular diseases	Gabon	[86]
			Margose	Leaf, Fruit, Seed	Direct Consumption of Leaf, Juice, Soaking of seeds in water	Diabetes, High Cholesterol Level	Mauritius	[113]
				Leaf, Fruit		Diabetes	Nigeria	[122]
			Monamelala	Leaf, Whole plant, Potherb	Cooking, Maceration	Diabetes, Hypertension	South Africa	[99,141,146]
			Bitter melon	Fruit	Juice, Cooking	Diabetes	Tanzania	[169]
			Katchalayo, Agnagran	Whole plant	Infusion	Diabetes, Hypertension	Togo	[170,173]
*Momordica foetida* Schumach.	Cucurbitaceae		Iphunzu	Leaf	Decoction	Diabetes	Kenya	[96]
			Intshungu	Leaf, Stem, Root, Whole plant	Decoction, Juice, Tea	Diabetes, Hypertension	South Africa	[99,142,146]
*Morinda lucida* Benth.	Rubiaceae	African peach	Owouwo, Houinsi / Wetin	Root, Leaf, Bark	Decoction	Diabetes	Benin	[47]
			Bokakate, Mulambu	Leaf, Bark, Root	Maceration, Decoction	Diabetes	DR Congo	[66]
				Leaf, Bark, Root	Decoction	Diabetes	Cote D’Ivoire	[70]
			Dungatsi, Akeng	Leaf, Bark	Decoction	Diabetes	Gabon	[88]
			Sido	Leaf		Hypertension	Nigeria	[123]
			Zanklan, Zaklam	Leaf	Decoction	Diabetes, Hypertension	Togo	[170,173]
*Morinda morindoides* (Baker) Milne-Redh.	Rubiaceae		Kongobololo, Mesokhama	Leaf	Decoction	Diabetes	DR Congo	[66]
				Root	Decoction	Diabetes	Cote d’Ivoire	[70]
*Moringa oleifera* Lam.	Moringaceae	Horseradish tree, Miracle tree Drumstick tree, Behen tree	Lagalaga, Kpatinman	Leaf, Seed	Powder, Triturating, Sauce/Direct Consumption	Diabetes, Hypertension	Benin	[23,47]
			Arzan tiiya	Leaf	Powder	Diabetes	Burkina Faso	[54]
			Moringa	Leaf	Juice	Diabetes, Hypertension	Eritrea	[23,72]
			Bagaruwa, Moringa	Leaf, Stem	Decoction	Diabetes, Hypertension	Ghana	[23,35,89]
				Leaf, Root		Diabetes	Guinea	[90]
			Moringa	Seed	Chewing	Diabetes	Kenya	[98]
			Brede mourongue	Leaf, Stem, Root	Juice, Decoction	Diabetes, High Cholesterol level, Hypertension	Mauritius	[23,113]
			Igbale, Zogale	Leaf, Bark, Root	Decoction, Infusion	Diabetes, Hypertension, Stroke	Nigeria	[23,34,128,214]
			Moringa			Diabetes, Hyperlipidemia, Hypertension, Obesity	Sierra Leone	[23,138]
			Makgonatsohle	Seed, Leaf	Cooking	Diabetes	South Africa	[141]
			Mlonge, Moringa	Flower, Pod, Seed, Leave, Root	Powder	Diabetes	Tanzania	[165,169]
			Segueleguedi, Yovoviti	Seed	Powder	Diabetes, Hypertension	Togo	[23,170,173]
						Diabetes, Hypertension	Uganda	[215]
				Leaf, Seed, Root	Decoction	Diabetes	Zambia	[177]
*Mucuna pruriens* (L.) DC.	Fabaceae	Velvet bean		Whole plant	Decoction	Diabetes	Cote d’Ivoire	[70]
			Werepe, Agbala	Leaf, Seed	Juice	Diabetes, Hypertension	Nigeria	[123,124]
*Musa acuminata* Colla	Musaceae		Banane	Fruit	Eating	Diabetes	Mauritius	[113]
			Ihliziyo, Kabhanana ebomvu	Flower bracts	Decoction	Hypertension	South Africa	[99,153]
				Whole	Cooking	Diabetes	Zambia	[177]
*Musa paradisiaca* L.	Musaceae	Plantain	Doboroagbamgba, Kokwe sozou	Fruit	Powder	Diabetes	Benin	[47]
						Cardiovascular disease	Cameroon	[59]
			Plantain, Digondi	Skin, Fruit, Root, Leaf, Stem	Burning, Juice, Maceration	Diabetes	Gabon	[87,88]
			Akondro	Leaf, Fruit		Diabetes	Madagascar	[102]
			Oghede	Fruit, Stem	Maceration	Hypertension	Nigeria	[123]
*Musanga cecropioides* R.Br. ex Tedlie	Urticaceae	Umbrella tree	Divala, Parassolier, Aseng	Leaf, Stem bark	Decoction, Maceration	Cardiovascular diseases, Diabetes	Gabon	[86,87,88]
			Ebe iehara	Leaf		Hypertension	Nigeria	[123]
*Nasturtium officinale* R. Br	Brassicaceae		Anandrano	Leaf		Hypertension	Madagascar	[103]
			Cresson	Leaf	Juice	Diabetes	Mauritius	[113]
*Nauclea diderrichii*	Rubiaceae					Diabetes	Benin	[91]
				Bark		Diabetes	Cameroon	[91]
			Bilinga	Stem bark	Decoction	Diabetes	Gabon	[87]
*Nauclea latifolia* Sm.	Rubiaceae	African peach		Root		Diabetes, Hypertension	Benin	[91]
				Root		Diabetes	Congo	[91]
				Stem bark, Root	Decoction	Diabetes	Cote d’Ivoire	[70,91]
			Tafashia	Root	Decoction	Hypertension	Ghana	[89]
				Leaf, Fruit, Bark, Stem bark, Root		Diabetes	Guinea	[90,91]
			Egbesi	Leaf, Stem bark, Root		Hypertension	Nigeria	[34,91,123]
						Heart disease	Sierra Leone	[139]
			Karmadoda	Fruit, Leaf	Infusion	Diabetes, Hypertension	Sudan	[156,159]
			Gnimon	Root	Decoction	Diabetes	Togo	[173]
*Newbouldia laevis* (P. Beauv.) Seem.	Bignoniaceae		Akoko, Kpatin	Leaf, Bark	Decoction	Diabetes	Benin	[47]
			Ossome-dzo	Stem Bark	Decoction	Diabetes	Gabon	[87,88]
			Dufin, Ako	Root		Stroke	Nigeria	[34]
*Nicotiana tabacum* L.	Solanaceae		Taba, Kla	Leaf	Powder, Triturating	Diabetes	Benin	[47]
			Assara			Hypertension	Togo	[170]
*Nigella sativa* L.	Ranunculaceae	Black cumin	Abosoda	Seed	Added in bread, Powder	Diabetes	Eritrea	[72,73]
			Al Haba Alsoda	Seed		Diabetes	Sudan	[156]
*Ocimum americanum* L.	Lamiaceae		Hizihizi, Kessou kessou	Root, Leaf	Decoction, Maceration	Diabetes	Benin	[47]
			Kozossonya			Hypertension	Togo	[170]
*Ocimum basilicum* L.	Lamiaceae		Seseg	Leaf	Crushing, Boiling	Hypertension	Eritrea	[74]
			Mutaa	Whole plant, Leaf	Decoction, Infusion	Diabetes	Kenya	[96]
				Leaf	Poultice	Hypertension	Ghana	[35]
			Lhbaq	Leaf	Decoction	Diabetes	Mauritania	[112]
						Hypertension	Senegal	[216]
			Timie	Leaf, Stem	Infusion	Heart problems, Hypertension	South Africa	[7,99]
*Ocimum gratissimum* L.	Lamiaceae	Scent leaf	Aribra, Chiayo	Leaf	Decoction, Triturating, Powder	Diabetes	Benin	[47]
				Leaf	Decoction	Diabetes	Cote d’Ivoire	[70]
			Nunum	Leaf	Decoction	Hypertension	Ghana	[35,89]
			Ebewadu	Leaf	Decoction	Diabetes, Hypertension	Nigeria	[123,125,134]
			Kounozorou, Esrou	Leaf	Infusion	Diabetes	Togo	[170,173]
*Olea europaea* L.	Oleaceae	Wild olive	Molialundi	Stem bark, Root	Decoction	Diabetes	Kenya	[96]
			Mohloare	Leaf, Stem		Hypertension	Lesotho	[42]
			Zolive	Leaf	Infusion	Cardiovascular disease, Diabetes, Hypertension	Mauritius	[23,113]
			uMquma, Umnquma, Olienhout, Olienhoutboom, Mutlhwari	Leaf, Bark, Root	Boiling, Decoction	Diabetes, Heart problems, Hypertension	South Africa	[7,99,147,149]
*Opilia amentacea* Roxb.	Opiliaceae		Gnandoro, Touahantouman	Leaf	Decoction	Diabetes	Benin	[47]
			Domfadou			Diabetes, Hypertension	Togo	[170]
*Oxytenanthera abyssinica*(A. Rich.) Munro	Poaceae		Dawe	Leaf, Root	Decoction, Maceration	Diabetes	Benin	[47]
				Leaf	Decoction	Diabetes	Cote d’Ivoire	[70]
				Leaf	Decoction	Diabetes	Togo	[173]
*Parkia biglobosa* (Jacq.) G.Don	Fabaceae	African locust bean	Ougba, Dumbu	Leaf, Bark, Root, Fruit		Diabetes, Hypertension	Benin	[17,199]
			Roaga	Root	Charred	Heart disorders, Hypertension	Burkina Faso	[54]
				Leaf	Decoction	Diabetes	Cote d’Ivoire	[70]
			Dawadawa/Dorowa	Leaf, Root	Decoction	Diabetes, Hypertension	Ghana	[35,89]
				Stem bark		Diabetes, Hypertension	Nigeria	[123,217]
			Soulou, Ewati	Leaf	Decoction	Diabetes, Hypertension	Togo	[170,173,217]
*Parquetina nigrescens* Afzel.	Apocynaceae		Ikpiarelokhae	Leaf	Infusion	Hypertension	Nigeria	[123]
			Bovoin, Atobo, Tobo		Maceration	Heart pains	Togo	[172]
*Pennisetum purpureum* Schumach.	Poaceae		Mikuku	Stem	Maceration	Diabetes	Gabon	[87,88]
			Orubingo	Leaf	Roasting	Heart disease	Uganda	[176]
*Pentaclethra macrophylla*	Mimosaceae			Fruit	Maceration	Cardiovascular disease	Cameroon	[56]
			Ukana	Seed	Cooked as soup	Hypertension	Nigeria	[132]
*Pentanisia prunelloides*	Rubiaceae	Wild verbena, Broad-leaved pentanisia	Setima-mollo	Leaf, Root		Diabetes, Heart problems, Hypertension	Lesotho	[42]
			Icimamlilo	Leaf, Rhizome, Corm, Root	Extract	Diabetes, Hypertension	South Africa	[99,147]
*Peperomia pellucida* (L.) Kunth	Piperaceae		Pepper-elder	Leaf	Infusion	Diabetes, Hypertension	Gabon	[87,88,218]
				Leaf		Diabetes, Hypertension	Nigeria	[125,218]
*Pericopsis laxiflora* (Baker) Meeuwen	Fabaceae		Ichedoun, Sedon	Leaf	Decoction	Diabetes	Benin	[47]
			Tchamani			Hypertension	Togo	[170]
*Persea americana* Mill.	Lauraceae	Avocado pear, Avocado	Egui avoka, Avokatin	Leaf	Decoction	Diabetes, Hypertension	Benin	[23,47]
						Diabetes	Cameroon	[59]
			Nzete ya savoka, Ivoka, Avocati	Leaf, fruit	Decoction	Diabetes	DR Congo	[66,67]
				Leaf	Decoction	Diabetes	Cote d’Ivoire	[70]
			Avocado, Muvoka	Leaf, Seed	Maceration, Decoction	Diabetes	Gabon	[87,88]
			Paya ahaban	Root	Decoction	Hypertension	Ghana	[23,89]
			Piya	Leaf, Seed	Decoction	Diabetes, Hypertension	Guinea	[23,90,92]
			Mukurobi	Leaf, Bark	Decoction	Diabetes	Kenya	[98]
			Avocat	Fruit	Juice	High Cholesterol Level, Hypertension	Mauritius	[23,113]
			Olumueb, Ube, Eben mbakara	Leaf, Seed, Fruit	Powder	Hypertension	Nigeria	[23,34,122,123,125,132]
			Moafokhathe	Leaf, Pulp, Fruit, Root	Cooking, Decoction	Diabetes; Hypertension	South Africa	[23,99,141]
				Stem bark	Decoction	Hypertension, Palpitation	Swaziland	[184]
			Mparachichi, Mwembe, Mafuta, Avocado	Leaf, Fruit, Seed, Rind, Bark, Whole plant		Diabetes	Tanzania	[165,169]
			Paya, Peya	Leaf	Decoction	Diabetes, Hypertension	Togo	[23,170,173]
				Leaf, Seed	Decoction, Infusion	Hypertension	Uganda	[176]
*Persea gratissima* Gaertn.	Lauraceae		Pia, Fia	Leaf, Bark	Decoction	Hypertension	Cameroon	[60]
			Avocatier	Leaf	Decoction	Cardiovascular diseases	Gabon	[86]
*Petroselinum crispum* (Mill). Fuss	Apiaceae		Parsley, Persil	Leaf	Chewing	Diabetes	Gabon	[87,88]
			Persil	Leaf	Decoction, Juice	Diabetes, High Cholesterol level, Hypertension	Mauritius	[113]
*Phaseolus vulgaris* var. *aborigineus* (Burkart) Baudet	Fabaceae					Diabetes	Cameroon	[59]
				Pod	Decoction	Diabetes	Cote d’Ivoire	[70]
			Cishimbo, Mukenji, Maharagwe	Green pod	Decoction	Diabetes	DR Congo	[67]
			Bean	Fruit	Decoction	Diabetes	Gabon	[87,88]
			Haricot vert	Pod	Decoction	Diabetes	Mauritius	[113]
			Sona, Ayi	Clove	Decoction	Diabetes	Togo	[170,173]
*Philenoptera cyanescens* (Schum. & Thonn.) Roberty	Fabaceae		Elou, Aho	Root	Decoction	Diabetes	Benin	[47]
			Tchele	Stem bark	Decoction	Hypertension	Togo	[171]
*Phyllanthus amarus* Schumach. & Thonn.	Phyllanthaceae		Aribissohou, Hlinwe	Whole plant, Leaf	Decoction	Diabetes	Benin	[47]
				Leaf	Decoction, Infusion, Maceration, Tincture	Diabetes	Cote d’Ivoire	[70]
				Whole plant	Decoction	Diabetes, Hyperglycaemia	Nigeria	[219]
			Mzalia nyuma	Leaf		Diabetes	Tanzania	[169]
			Seni-seniyo, Ahlinvi	Whole plant	Decoction	Diabetes, Hypertension	Togo	[170,173]
*Phyllanthus niruri subsp. lathyroides* (Kunth) G.L.Webster	Phyllanthaceae	Phyllanthus plant	Keelaneli	Leaf	Decoction	Diabetes	Mauritius	[113]
			Eyinbisowo	Leaf	Decoction	Diabetes	Nigeria	[126]
*Physalis peruviana*	Solanaceae		Mbuma, Mbupuru, Umuhire	Aerial part, Fruit, Leaf	Decoction	Diabetes	DR Congo	[67,220]
					Decoction	Diabetes	Kenya	[95]
*Picralima nitida*	Apocynaceae	Picralima	Abere, Ayokpe	Seed	Decoction, Maceration, Powder	Diabetes	Benin	[47]
			Ebam, Dugundu	Leaf, Fruit, Stem, Bark, Seed	Decoction, Maceration, Infusion	Cardiovascular diseases, Diabetes	Gabon	[86,87,88]
			Abere	Root	Decoction	Diabetes, Hypertension	Ghana	[35]
			Abeere	Seed	Infusion	Diabetes	Nigeria	[126]
			Amberi, Ayokpe	Seed	Decoction	Diabetes, Hypertension	Togo	[170,173]
*Piliostigma thonningii* (Schum.) Milne-Redh.	Fabaceae		Barke	Stem bark	Decoction	Hypertension	Guinea	[92]
			Bakou, Klo	Leaf	Decoction	Diabetes, Hypertension	Togo	[170,173]
*Piper guineense*	Piperaceae		Idjaye, Ninninkoun	Seed	Decoction, Maceration, Powder	Diabetes	Benin	[47]
			Njilulu	Bark	Decoction	Diabetes	DR Congo	[67]
				Fruit	Decoction	Diabetes	Cote d’Ivoire	[70]
			Abo-Me-Nzang-Ndic		Decoction	Cardiovascular diseases	Gabon	[86]
						Hypertension	Nigeria	[125]
*Piper umbellatum* L.	Piperaceae		Fouboren, Abomedzana, Bepoie, Meuboue	Leaf	Paste, Maceration	Hypertension	Cameroon	[60,221]
			Abo-Me-Nzang		Decoction	Cardiovascular Diseases	Gabon	[86]
*Plumbago zeylanica* L.	Plumbaginaceae	Leadwort	Anan, Dangblan	Leaf	Powder	Diabetes	Benin	[47]
			Aftooh	Root, Stem	Decoction	Diabetes	Eritrea	[72]
			Amira	Leaf		Heart disease	Ethiopia	[76]
*Portulaca oleracea* L.	Portulacaceae		Nkekeih, Deung-Deung	Leafed-stem	Paste, Maceration	Hypertension	Cameroon	[60]
				Stem, Leaf	Decoction	Diabetes	Cote d’Ivoire	[70]
*Prosopis africana* (Guill. & Perr.) Taub.	Fabaceae		Prekese	Seed, Leaf	Decoction	Hypertension	Ghana	[89]
			Kpalou	Stem bark, Root	Powder, Decoction	Diabetes	Togo	[170,171]
*Pseudocedrela kotschyi* (Schweinf.) Harms	Meliaceae	Savanna woodland tree	Chahizi, Atindokpwe, Tchaguidi, Bisisumbu	Leaf, Bark, Root	Decoction	Diabetes, Hypertension	Benin	[47,49,199]
			Ti-tore	Stem Bark	Decoction	Hypertension	Burkina Faso	[54]
			Tuna	Root	Decoction	Hypertension	Ghana	[89]
			Tuna	Root	Decoction	Diabetes	Nigeria	[128]
			Ditotore			Diabetes, Hypertension	Togo	[170]
*Psidium guajava* L.	Myrtaceae	Guava	Kekoun, Kinkountin	Leaf, Root	Decoction	Diabetes	Benin	[47]
						Diabetes	Comoros	[65]
				Leaf	Decoction	Diabetes	Cote d’Ivoire	[70]
			Zeitun	Leaf	Decoction	Diabetes	Eritrea	[72]
			Goyavier, Guava	Leaf, Stem bark	Decoction, Maceration	Cardiovascular diseases, Diabetes	Gabon	[86,87,88]
				Leaf		Diabetes	Guinea	[90]
			Goyave	Leaf, Fruit	Infusion, Consume Ripe Fruit, Juice	Diabetes	Mauritius	[113]
			Gova, Guofa	Leaf		Hypertension, Stroke	Nigeria	[34,123,125]
			Koejawel, Ugwava	Leaf, Root	Decoction, Infusion	Diabetes, Hypertension	South Africa	[99,142,153,222]
			Umgwava	Leaf	Infusion	Hypertension, Palpitation	Swaziland	[184]
			Goyaba			Diabetes	Togo	[170]
*Pteleopsis suberosa* Engl. & Diels	Combretaceae		Okroukrou, Klui-Klui	Leaf, Bark	Decoction, Powder	Diabetes	Benin	[47]
			Sissinon, Sisinon	Stem bark	Powder	Diabetes, Hypertension	Togo	[170,171]
*Pterocarpus erinaceus* Poir	Fabaceae		Nonoigna	Leaf	Charred	Hypertension	Burkina Faso	[54]
			Igiara	Bark		Hypertension	Nigeria	[34]
			Tem			Diabetes	Togo	[170]
*Punica granatum* L.	Lythraceae		Grenade	Fruit	Juice	Cardiovascular disease, High Cholesterol level	Mauritius	[113]
			Mokgarenate, Granaat	Root	Cooking, Infusion	Diabetes	South Africa	[141,147]
*Pyrenacantha kaurabassana* Baill.	Icacinaceae		Njovu yayenda, Chikhazika, Mambo, Vindindi	Underground part	Powder	Heart palpitations, Hypertension	Malawi	[223]
			Insema	Bulb, Tuber	Decoction	Hypertension	South Africa	[99,153]
*Rauvolfia caffra*	Apocynaceae		Mutalala	Leaf, Stem		Diabetes	DR Congo	[69]
			umHlambamanzi	Stem, Bark, Whole plant		Hypertension	South Africa	[99]
*Rauvolfia vomitoria* Afzel.	Apocynaceae	Swizzle stick	Lewe	Root	Decoction	Diabetes	Benin	[47]
			Abude	Leaf	Decoction	Heart ache	Cameroon	[56]
			Isumbubululu	Root bark	Decoction	Diabetes	DR Congo	[66]
				Leaf	Infusion	Diabetes	Cote d’Ivoire	[70]
			Mupitugu	Leaf, Root, Bark	Decoction, Maceration	Diabetes	Gabon	[88]
			Akata, Asofeyeje	Root, Bark, Stem, Leaf	Decoction	Diabetes, Hypertension	Nigeria	[123,125,126]
*Rhamnus prinoides* L. Her	Rhamnaceae	African dogwood, Camdeboo stinkwood, Glossy-leaf	Mukarakinga	Roots, Bark	Decoction	Diabetes	Kenya	[98]
			Mofifi			Diabetes	Lesotho	[42]
			Blinkblaar, Mofifi	Branches	Decoction	Diabetes	South Africa	[147]
*Ricinus communis* L.	Euphorbiaceae			Whole plant	Decoction	Diabetes	Cote d’Ivoire	[70]
			Laara	Seed		Hypertension	Nigeria	[34]
			Umhlakuva, Impono	Leaf	Decoction	Hypertension	South Africa	[99,153]
			Mbarika, Mkale	Leaf, Root		Stroke	Tanzania	[166]
			Dedele			Hypertension	Togo	[170]
*Rosmarinus officinalis* L.	Lamiaceae	Rosemary	Azmarino	Leaf, Stem	Added to food	Diabetes	Eritrea	[72]
			Romarin	Leaf	Decoction	Cardiovascular disease	Mauritius	[113]
			Roosmaryn	Leaf	Decoction, Infusion	Diabetes, Obesity	South Africa	[140,147]
*Rumex abyssinicus* Jacq.	Polygonaceae		Meqmoqo, Mekmeko	Root		Diabetes, Hypertension	Ethiopia	[76,82,224]
						Diabetes, Hypertension	Cameroon	[224]
*Rumex lanceolatus*	Polygonaceae	Small dock, Smooth dock, Common dock	Khamane	Leaf, Root		Diabetes	Lesotho	[42]
			Gladdetongblaar	Leaf, Root	Decoction	Diabetes	South Africa	[147]
*Saccharum officinarum* L.	Poaceae		Okpa-soucre, Leke	Root, Leaf	Decoction	Diabetes	Benin	[47]
						Hypertension	Cameroon	[59]
			Sugar cane	Wine, Leaf	Fermentation	Diabetes	Gabon	[88]
				Outer part	Maceration	Diabetes	Zambia	[177]
*Salvia officinalis* L.	Lamiaceae			Leaf	Infusion	Diabetes	DR Congo	[67]
			Maaramya	Leaf		Diabetes	Sudan	[156]
*Sarcocephalus latifolius* (Sm.)	Rubiaceae		Igbessin, Ko	Root	Decoction, Maceration, Powder	Diabetes	Benin	[47]
				Stem bark	Decoction	Diabetes	Cote d’Ivoire	[70]
			Um dimy	Root, Fruit	Infusion	Diabetes	Sudan	[159]
			Kidjitchilou			Diabetes, Hypertension	Togo	[170]
*Schwenckia americana* L.	Solanaceae		Dandana	Leaf, Bark, Root	Decoction, Maceration	Diabetes	Nigeria	[128]
			Kotoka			Hypertension	Togo	[170]
*Sclerocarya birrea* (A. Rich.) Hochst	Anacardiaceae		Edi	Leaf	Decoction	Diabetes	Cameroon	[56]
				Leaf	Powder	Diabetes	Cote d’Ivoire	[70]
						Diabetes	Nigeria	[192]
			Maroela	Bark, Leaf, Stem, Stem-Bark	Decoction	Diabetes, Hypertension	South Africa	[99,142]
			Hommaid	Bark, Stem Bark	Maceration	Diabetes	Sudan	[156,158]
			Mupfura	Root	Steaming	Diabetes	Zimbabwe	[53]
*Scoparia dulcis* L.	Plantaginaceae		Jomboa	Leaf, Branchlet	Maceration	Stroke	Cameroon	[56]
				Whole plant	Decoction	Diabetes	Cote d’Ivoire	[70]
						Diabetes	Guinea	[90]
			Noumayi	Leaf	Decoction	Diabetes	Togo	[173]
*Searsia lancea*	Anacardiaceae	Karee	Ts’ilabele	Leaf, Fruit		Diabetes, Heart problems, Hypertension	Lesotho	[42]
			Karee, Rooikaree	Leaf, Fruit	Decoction, Infusion	Diabetes	South Africa	[147]
*Securidaca longepedunculata* Fresen.	Polygalaceae	Coffee senna	Kpatale, Kpata	Root	Decoction, Powder	Diabetes	Benin	[47]
				Whole plant	Decoction	Diabetes	Cote d’Ivoire	[70]
				Root	Poultice	Stroke	Ghana	[35]
			Omudhiku			Stroke	Namibia	[121]
			Sanya	Leaf	Maceration	Diabetes	Nigeria	[128]
			Mbazo	Root	Infusion	Heart pains (palpitations)	Tanzania	[168]
			Fozi, Tritou	Root, Leaf + Root	Powder, Decoction	Diabetes, Hypertension	Togo	[170,171,173]
*Sesamum indicum* L.	Pedaliaceae	Sesame	Gingeli, Sesame			Hypertension	Mauritius	[115,118]
			Simsim	Seed		Diabetes	Sudan	[156]
*Senna alata* (L.) Roxb.	Fabaceae		Gitsamuna	Leaf, Seed, Root	Decoction	Diabetes	Gabon	[88]
			Osempe	Leaf	Decoction	Diabetes	Ghana	[35]
			Quatre epingles	Leaf		Hypertension	Madagascar	[102]
			Quatre epingles	Leaf	Decoction	Hypertension	Mauritius	[113]
				Leaf	Maceration	Diabetes, Hypertension	Nigeria	[225,226]
*Senna occidentalis* (L.) Link	Fabaceae		Muwiwisi	Leaf, Root	Decoction	Diabetes	Gabon	[88]
			Tsotsorinangatra	Stem		Hypertension	Madagascar	[30]
			Cassepiante	Seed	Grilling, Soaking	Hypertension	Mauritius	[113]
						Hypertension	Nigeria	[125]
			Bun Balash/Soreib	Seed	Decoction, Infusion	Diabetes	Sudan	[159,227]
			Bessisan	Seed	Decoction	Diabetes	Togo	[173]
*Senna siamea* Lam.	Caesalpiniaceae		Cassia, Kenoun	Root/Bark	Decoction	Diabetes	Benin	[47]
			Zangarati	Root	Decoction	Diabetes	Togo	[173]
*Senna singueana* (Del.) Lock	Caesalpiniaceae			Leaf, Stem bark		Diabetes	Burkina Faso	[226]
			Mukengeka	Leaf, Stem Bark	Decoction	Diabetes	Kenya	[96,226]
				Leaf, Stem bark		Diabetes	Tanzania	[226]
*Solanum americanum*	Solanaceae		Mulunda	Leaf	Decoction	Diabetes	DR Congo	[67]
			Shwiga	Leaf		Diabetes	Tanzania	[169]
*Solanum melongena* L.	Solanaceae		Anguive		Cooking	Diabetes	Mauritius	[113]
			Nya	Fruit	Cooking as soup	Diabetes	Nigeria	[132]
*Solanum nigrum* L.	Solanaceae		Makeke	Fruit	Decoction	Diabetes	DR Congo	[66]
			Managu	Leaf	Infusion	Diabetes	Kenya	[98]
			Munafoqow	Whole plant	Boiling	Cardiac complaints	Somalia	[212]
*Spondias mombin* L.	Anacardiaceae	Hog plum	Egui iwewe, Akikontin	Leaf, Bark, Root, Fruit	Powder, Maceration, Decoction, Infusion	Diabetes	Benin	[47]
				Stem bark, Root		Hypertension	Nigeria	[123]
			Aklicon	Leaf	Decoction	Diabetes	Togo	[173]
*Sida acuta*	Malvaceae	Broom weed, Horn bean	Mudundu	Bark	Decoction	Diabetes	DR Congo	[67]
			Aihenmmwin, Iseketu	Leaf, Stem	Decoction, Infusion	Diabetes, Hypertension	Nigeria	[123,126]
*Sida rhombifolia* L.	Malvaceae		Zimben, Ndjicje	Leaf	Paste, Maceration	Hypertension	Cameroon	[60,228]
				Leaf	Maceration	Hypertension	DR Congo	[228]
*Solanum incanum* L.	Solanaceae	Poison berry	Tangalanga	Leaf	Decoction	Diabetes, Heart ache	Cameroon	[56,59]
			Uengule	Fruit	Boiling	Diabetes	Eritrea	[72]
			Mukondu, Mutungu	Leaf	Decoction	Diabetes	Kenya	[96]
*Solanum lycopersicum* L.	Solanaceae	Tomato	Tomato	Leaf	Maceration, Filtration	Hypertension	Central African Republic	[64]
				Fruit	Decoction	Diabetes	Cote d’Ivoire	[70]
			Pomme d’amour	Fruit	Juice	Cardiovascular disease	Mauritius	[113]
			Tumatur		Eat fruit	Diabetes	Nigeria	[128]
			Timati			Hypertension	Togo	[170]
*Solanum tuberosum* L.	Solanaceae					Diabetes	Cameroon	[59]
			Pomme de terre	Tuber	Juice	Diabetes	Mauritius	[113]
*Sonchus oleraceus* L.	Compositae		Muthunga	Leaf	Chewing, Boiling	Diabetes	Kenya	[98]
			Epinard	Leaf	Juice	High Cholesterol level	Mauritius	[113]
*Spathodea campanulata* P.Beauv.	Bignoniaceae		Cifulula, Langalanga, Mbalimbali	Bark	Decoction	Diabetes	DR Congo	[67]
			Tulipier du Gabon	Leaf, Stem bark	Decoction	Diabetes	Gabon	[88]
*Stachytarpheta angustifolia*	Verbenaceae		Iru alangba	Leaf, Whole plant		Hypertension	Nigeria	[34]
			Tchoumboulouzou			Hypertension	Togo	[170]
*Steganotaenia araliacea* Hochst.	Apiaceae	Carrot tree	Mewets denagl	Leaf, Seed	Decoction	Diabetes	Eritrea	[72]
			Muvuavui	Leaf	Decoction	Diabetes	Kenya	[96]
			Omuwanula	Leaf	Decoction	Diabetes	Uganda	[31]
*Sterculia setigera* Delile	Malvaceae		Akporo, Azilokoue	Bark	Decoction, Maceration	Diabetes	Benin	[47]
			Pumpugga	Stem bark	Decoction	Heart disorders	Burkina Faso	[54]
				Pulp, Gum		Heart trouble	Togo	[229]
*Stereospermum kunthianum* Cham.	Bignoniaceae		Hounsadii	Bark		Diabetes	Benin	[47]
				Leaf	Decoction	Diabetes	Cote d’Ivoire	[70]
*Striga hermonthica* (Delile) Benth.	Orobanchaceae		Ki-waogo	Whole plant	Juice	Heart disorders	Burkina Faso	[54]
			Boda, Al-buda	Aerial part, Whole plant	Maceration	Diabetes	Sudan	[156,159]
*Strophanthus hispidus* DC.	Apocynaceae		Chaaro, Adikoun	Root	Decoction	Diabetes	Benin	[47]
			Getsele, Ndegle	Root		Heart conditions	Guinea-Bissau	[94]
*Strychnos henninggsii* Gilg.	Loganiaceae	Red bitter berry, Coffee hard pear, Coffee- bean Strychnos, Hard pear	Muteta	Leaf	Decoction	Diabetes	Kenya	[96]
			Umanana, Bosolienhout, Muramba-kolodza	Bark	Powder	Diabetes	South Africa	[147]
*Strychnos pungens* Soler.	Loganiaceae		Mukome	Leaf, Fruit, Root		Heart pains	Tanzania	[166]
				Root	Decoction	Diabetes	Zambia	[177]
*Strychnos spinosa* Lam.	Loganiaceae		Katinpoaga	Leaf	Powder	Heart disorders	Burkina Faso	[54]
			Sansa, Kisongole	Bark, Root		Diabetes	DR Congo	[69]
			Umm Bekhesa	Fruit	Eaten	Hypertension	Sudan	[159,227]
			Kpengbele			Diabetes	Togo	[170]
*Swartzia madagascariensis* Desv.	Fabaceae			Root	Decoction	Diabetes	Benin	[47]
				Root	Decoction	Diabetes	Zambia	[177]
*Syzygium cumini* (L.) Skeels	Myrtaceae		Telezia	Fruit	Decoction	Diabetes	DR Congo	[66]
			Jamblon	Leaf, Fruit, Seed	Decoction, Consume ripe Fruits, Juice	Diabetes	Mauritius	[113]
			Mudisi, Mzambarau, Black plum, Mzambarau	Seed, Bark, Stem		Diabetes	Tanzania	[166,169]
*Syzygium guineense*	Myrtaceae		Ko kissa	Bark	Decoction	Hypertension	Mali	[230]
				Leaf		Diabetes	Nigeria	[230]
			Muvengi			Diabetes	Tanzania	[169]
*Tacca leontopetaloides* (L.) Kuntze	Taccaceae		Ossangni, Zinwohouehe	Bulb, Tuber	Powder, Decoction	Diabetes	Benin	[47]
			Nanitiwou			Diabetes	Togo	[170]
*Tamarindus indica* L.	Fabaceae	Tamarind tree, Tamarind	Egui ariran, Jevivi	Bark	Decoction	Diabetes	Benin	[47]
			Pusga	Stem bark	Charred	Heart disorders	Burkina Faso	[54]
				Leaf, Root, Stem bark	Decoction	Diabetes	Cote d’Ivoire	[70]
			Humer	Fruit	Extract	Diabetes	Eritrea	[72]
			Dyabbhe	Leaf, Stem bark	Decoction	Diabetes, Hypertension	Guinea	[90,92]
			Kithumula	Fruit, Bark, Root	Decoction	Diabetes	Kenya	[96]
			Tamarin	Fruit	Juice	Hypertension	Mauritius	[113]
			Tsamiya	Bark, Root	Decoction	Diabetes	Nigeria	[128]
			Mkwedu, Mkwaju	Leaf, Root	Juice, Decoction	Heart pains	Tanzania	[231]
			Enkoge, Omuhuwa	Fruit pulp	Juice	Hypertension	Uganda	[174]
*Tapinanthus globiferus* (A.Rich.) Tiegh.	Loranthaceae		Lisua-la-kote	Leaf, Flower	Decoction	Diabetes	Cameroon	[56]
			Afomo, Kauchi	Leaf	Decoction	Diabetes, Hypertension	Nigeria	[124]
*Tarchonanthus camphoratus* L.	Asteraceae		Galqaddo	Leaf	Soaking, Crushing	Diabetes	Djibouti	[71]
				Root	Cooking	Diabetes	South Africa	[141]
*Tephrosia capensis*	Fabaceae		Pelo-li-maroba	Root		Heart palpitations/problems	Lesotho	[42]
				Root	Decoction	Hypertension	South Africa	[99]
*Terminalia catappa* L.	Combretaceae		Madame, Kalanga ya Wazungu	Leaf	Decoction	Diabetes	DR Congo	[66]
				Leaf	Decoction	Diabetes	Cote d’Ivoire	[70]
			Badamier	Leaf	Decoction	Diabetes	Mauritius	[113]
			Yovositi	Bark	Decoction	Diabetes	Togo	[173]
*Terminalia brownii* Fresen.	Combretaceae	Terminalia	Weiba	Stem bark, Leaf	Decoction	Diabetes	Eritrea	[72]
			Muuuku/Kiuuku	Stem bark	Decoction	Diabetes	Kenya	[96]
			Olbukoi	Bark		Hypertension	Tanzania	[166]
*Terminalia sericea*	Combretaceae	Silver terminalia, Silver cluster leaf	Mogonono	Stem bark	Powder	Diabetes	Botswana	[53]
			Vaalboom, Geelhout, Mususu	Bark		Diabetes	South Africa	[147]
			Mhungweluwala	Stem bark, Root	Powder	Diabetes	Tanzania	[169,231]
*Tetrapleura tetraptera* (Schum. & Thonn.) Taub.	Fabaceae	Ring worm bush	Aidan, Lindja	Fruit, Clove	Decoction, Powder	Diabetes	Benin	[47]
			Ndjapa, Nkwon, Sasas, Akpwa, Esesse	Bark	Maceration, Decoction	Hypertension	Cameroon	[60]
			Gyaga	Seed, Leaf, Stem Bark, Root	Decoction, Infusion	Diabetes	Gabon	[88]
			Prekese	Fruit	Decoction	Diabetes, Hypertension	Ghana	[35]
			Uhiokiriho, Asuwon	Fruit	Decoction	Cardiovascular activities, Diabetes	Nigeria	[126,187]
*Theobroma cacao* L.	Malvaceae		Cocoa ahaban	Leaf	Decoction	Hypertension	Ghana	[35,89]
			Coco			Diabetes, Hypertension	Togo	[170]
*Tithonia diversifolia*	Asteraceae		Cilula	Leaf	Decoction	Diabetes	DR Congo	[67]
			Daisy	Flower, Leaf	Chewing, Decoction	Diabetes	Gabon	[87,88]
			Tetegbe	Whole plant	Decoction	Diabetes	Togo	[173]
*Tribulus terrestris* L.	Zygophyllaceae		Qumutu Gala	All parts	Concoction	Heart disease	Ethiopia	[79]
			Diraisa	Root	Maceration	Diabetes	Sudan	[159]
*Trichilia emetica* var. laevicarpa Pellegr.	Meliaceae		Ichin igbe, Chivi	Root	Decoction	Diabetes	Benin	[47]
			Kkirs-taanga	Root	Decoction	Obesity	Burkina Faso	[54]
			Umathuzini, Umkhuhlu	Leaf, Fruit, Bark, Stem, Root	Decoction, Poultice	Hypertension	South Africa	[99,153]
			Adjendjegbizou, Adjindjinkpizou	Root	Powder, Decoction	Diabetes, Hypertension	Togo	[170,171]
			Akwirakwir	Root		Heart problem	Uganda	[175]
*Trifolium burchellianum*	Fabaceae		Moroko	Root		Heart problems	Lesotho	[42]
			Usithathi	Leaf, Stem, Root	Infusion	Hypertension	South Africa	[99]
*Trigonella foenum-graecum* L.	Fabaceae	Fenugreek	Abe’ake	Seed	Powder	Diabetes	Eritrea	[72]
			Methi	Seed	Decoction, Soaking	Lowering blood sugar level, Diabetes, High Cholesterol level	Mauritius	[113,115]
			Hilba	Seed	Decoction, Dessert	Diabetes	Sudan	[156,157,160]
*Tulbaghia acutiloba*	Alliaceae		Konofolo/Sefotha-fotha	Whole plant		Hypertension	Lesotho	[42]
			Ishaladi Lezinyoka	Bulb, Flower, Whole plant	Decoction, Infusion	Hypertension	South Africa	[99]
*Urtica dioica*	Urticaceae	Common nettle, Stinging nettle	Chachingi	Leaf	Tincture, Infusion	Diabetes	DR Congo	[67]
					Decoction	Diabetes	Kenya	[95]
				Leaf, Root	Infusion	Diabetes	South Africa	[147]
*Uvaria chamae* P.Beauv.	Annonaceae		Yaha, Ayadaha	Root	Decoction, Maceration, Powder	Diabetes	Benin	[47]
			Boyle	Leaf	Decoction	Diabetes, Hypertension	Guinea	[90,92]
			Agbana	Root	Decoction	Diabetes	Togo	[173]
*Vangueria apiculata* K.	Rubiaceae		Kirongomani, Mtigunda	Stem bark, Leaf	Decoction	Diabetes	Tanzania	[169]
			Matugunda	Roots	Decoction	Hypertension	Uganda	[38]
*Vangueria infausta*	Rubiaceae		Um Tulwa, Umvilo	Leaf, Bark	Infusion, Maceration	Hypertension	South Africa	[99]
			Amabungo, Mabungo	Stem bark, Leaf, Fruit, Seed	Decoction	Diabetes	Tanzania	[169]
*Vangueria madagascariensis* J.F.Gmel.	Rubiaceae		Vavangue	Leaf	Decoction	Diabetes, Hypertension	Mauritius	[113]
			Kir kir, Soum Eyowm	Fruit, Root	Maceration	Diabetes, Hypertension	Sudan	[156,159]
*Vernonia amygdalina* Delile	Compositae	Bitter leaf	Aroman, Amanvive	Bulb, Bark, Root, Leaf	Decoction, Powder, Maceration, Trituration	Diabetes	Benin	[47]
				Leaf	Decoction	Diabetes, Hyperglycaemia	Cameroon	[45,56,59]
			Kilulukunju, Mubirizi	Leaf	Decoction	Diabetes	DR Congo	[66,67]
			Grawa	Leaf, Stem Bark	Extract	Diabetes	Eritrea	[72]
			Ndole	Leaf	Chewing, Decoction	Diabetes	Gabon	[87,88]
			Oriwo, Ewuro, Etidod	Leaf, Stem, Root	Infusion, Cooked as soup	Diabetes, Hypertension	Nigeria	[34,45,122,123,125,132,134,192]
			Bitter Leaf			Hypertension, Obesity	Sierra Leone	[138]
			Umhlungu-hlungu	Leaf	Pulverization	Diabetes	South Africa	[53]
			Souwaka, Aloma	Leaf	Decoction	Diabetes, Hypertension	Togo	[170,173]
*Vernonia colorata*	Asteraceae			Leaf, Root		Heart failure	DR Congo	[45]
				Leaf	Decoction	Diabetes	Cote d’Ivoire	[70]
				Leaf, Root		Diabetes	South Africa	[45]
				Leaf, Root		Heart failure	Tanzania	[45]
			Ahalizoma	Leaf	Decoction	Diabetes	Togo	[173]
*Vernonia oligocephala*	Asteraceae	Silver leaved vernonia	Umhlungu-hlungu	Leaf, Twig, Root	Decoction, Infusion	Diabetes	South Africa	[45,53,147]
						Diabetes	Swaziland	[45]
*Vigna unguiculata* (L.) Walp.	Fabaceae		Benga	Flower	Maceration	Kid obesity, Heart disorders	Burkina Faso	[54]
			Ekoki, Konn, Wonda m’bale, Guin	Leaf	Paste, Maceration	Hypertension	Cameroon	[60]
					Decoction	Diabetes	Cote d’Ivoire	[70]
*Vitex doniana* Sweet	Lamiaceae	Black plum		Stem		Hypertension	Mali	[200]
			Dunya	Bark	Decoction	Diabetes	Nigeria	[128]
			Fonyi	Leaf, Root	Decoction	Diabetes	Togo	[173]
*Voacanga africana* Stapf ex Scott-Elliot	Apocynaceae		Binse	Root	Decoction	Hypertension	Central African Republic	[64]
			Ondou, Ontueles	Root	Maceration	Diabetes	Gabon	[87,88]
			Igi dodo	Stem		Hypertension	Nigeria	[34]
			Mberebere	Root	Decoction	Spasms of the heart	Tanzania	[231]
*Vitellaria paradoxa* C. F. Gaertn.	Sapotaceae		Emi, Limoutin	Bark	Decoction	Diabetes	Benin	[47]
			Taanga	Stem bark	Cream	Heart disorders	Burkina Faso	[54]
			Nkuto dua, Nkudua/mankade	Stem bark, Root	Decoction	Diabetes, Hypertension	Ghana	[35,89]
			Somou	Stem bark	Decoction, Powder	Diabetes, Hypertension	Togo	[170,171]
*Waltheria indica* L.	Malvaceae	Sleepy morning	Yankufa	Leaf	Maceration	Diabetes	Nigeria	[128]
			Adoueti	Root	Decoction	Diabetes	Togo	[173]
*Withania somnifera* (L.)	Solanaceae	Winter cherry	Agol	Root, Leaf	Root Immersion, Leaf’ juice	Diabetes	Eritrea	[72]
			Mwianzo	Root	Infusion	Diabetes	Kenya	[96]
			Mkubya	Root	Boiling	Diabetes	Tanzania	[169]
*Ximenia caffra*	Ximeniaceae			Whole plant		Obesity	Swaziland	[232]
				Root		Hypertension	Tanzania	[232]
*Xylopia aethiopica* (Dunal) A. Rich.	Annonaceae	African pepper, Ethiopian pepper	Oroun, Kpejelekoun	Fruit	Decoction, Maceration, Powder	Diabetes	Benin	[47]
			Mugana	Fruit, Leaf	Decoction	Diabetes	Gabon	[87,88]
			Guile	Leaf, Fruit	Decoction, Infusion	Diabetes, Hypertension	Guinea	[90,92,233]
			Unea, Eru Alomo	Leaf, Root, Fruit, Seed	Decoction	Diabetes; Hypertension	Nigeria	[34,123,233]
				Fruit	Decoction	Diabetes	Senegal	[233]
			Souzi	Fruit	Decoction	Diabetes	Togo	[170,233]
*Xysmalobium undulatum* (L.) W.T.Aiton	Apocynaceae	Milk bush, Milkwort, Wild cotton, Wave-leaved xysmalobium	Poho-ts’ehla	Root		Diabetes	Lesotho	[42]
			Bitterhout, Bitterwortel, iShinga	Root	Infusion, Decoction	Diabetes	South Africa	[147]
*Zanthoxylum chalybeum* Engl.	Rutaceae		Mukenea	Stem bark	Decoction, Infusion	Diabetes	Kenya	[96]
			Mulungulungu, Munungu, Oluisuki	Leaf, Bark, Root		Heart pains	Tanzania	[166]
				Root	Maceration	Diabetes	Zambia	[177]
*Zea mays* L.	Poaceae	Maize	Gbado, Gbade		Decoction	Diabetes	Benin	[47]
			Bazeuh, Go’o, Fonn, Mbassi, Geufeut	Female flower, ‘Corn beard’	Decoction	Diabetes, Hypertension	Cameroon	[59,60]
			Ndjo, Nkodji	Flowers	Boiling, Filtration	Hypertension	Central African Republic	[64]
			Muhindi, Cigonji	Filaments of Stamens	Decoction	Diabetes, Hypertension	DR Congo	[67]
			Maize, Putu	Fiber, Corn silk, Leaf stigma	Decoction	Diabetes	Gabon	[87,88]
				Seed		Hypertension	Nigeria	[123]
*Zingiber officinale* Roscoe	Zingiberaceae	Ginger	Atale, Dote	Root	Decoction, Maceration, Powder	Diabetes	Benin	[47]
			Jenjebel, Zingible	Seed, Root	Tea, Powder	Diabetes	Eritrea	[72,74]
			Nungutsi mabala	Rhizome, Root	Maceration	Diabetes	Gabon	[88]
			Gingembre	Root	Infusion	High Cholesterol Level	Mauritius	[113]
			Unien	Rhizome		Hypertension	Nigeria	[123,192]
*Ziziphus mistol* Griseb.	Rhamnaceae		Mugulga	Root	Decoction	Diabetes	Burkina Faso	[54]
				Leaf	Decoction	Diabetes	Cote d’Ivoire	[70]
			Sdar-lahbyl	Leaf	Powder, Maceration, Infusion	Diabetes, Hypertension	Mauritania	[112]
*Ziziphus mucronata* Willd	Rhamnaceae	Buffalo-thorn	Kiimes-mugla	Root	Decoction	Obesity	Burkina Faso	[54]
			Motalo, Nceseni, Mokgaloaloe	Leaf, Root	Tea	Diabetes	South Africa	[145,147]
			Mugugunu	Root, Bark		Hypertension	Tanzania	[166]

**Table 3:** Details of five most preferred medicinal plants in sub-Saharan African countries for the treatment of cardiovascular diseases and their associated risk factors.

Medicinal Plants	English Name	Families	SSA Countries with Reported Use	Number of SSA Countries	Rank
*Allium sativum* L.	Garlic	Amaryllidaceae	Benin, Burkina Faso, Cameroon, DR Congo Cote d’Ivoire, Eritrea, Ethiopia, Gabon, Guinea, Kenya, Madagascar, Mauritius, Nigeria, Sierra Leone, South Africa, Sudan Tanzania, Togo, Uganda, Zambia	20	1st
*Persea americana* Mill.	Avocado	Lauraceae	Benin, Cameroon, DR Congo, Cote d’Ivoire, Gabon, Ghana, Guinea, Kenya, Mauritius, Nigeria, South Africa, Swaziland, Tanzania, Togo, Uganda	15	2nd
*Moringa oleifera* Lam.	Drumstick tree	Moringaceae	Benin, Burkina Faso, Eritrea Ghana, Guinea, Kenya, Mauritius, Nigeria, Sierra Leone, South Africa, Tanzania, Togo, Uganda, Zambia	14	3rd
*Mangifera indica* L.	Mango	Anacardiaceae	Benin, Cameroon, DR Congo, Eritrea, Gabon, Ghana, Guinea, Kenya, Mauritius, Nigeria, Togo, Zambia, Zimbabwe	13	4th
*Allium cepa* L.	Onion	Amaryllidaceae	Benin, Burkina Faso, Cameroon, DR Congo, Eritrea, Ethiopia, Gabon, Mauritius, Nigeria, South Africa, Sudan, Togo	12	5th

**Table 4:** Major bioactive compounds in the five most preferred medicinal plants for the treatment of cardiovascular diseases in sub-Saharan Africa.

Rank	Medicinal Plants	Major Bioactive Compounds	Chemical Structure	Mechanism of Action of Cardioprotective Potential	References
1st	*Allium sativum* L.	Organosulfur compounds such as diallylthiosulfonate (allicin), diallyl sulfide, diallyl disulfide, diallyl trisulfide, E/Z-ajoene, S-allyl-cysteine, and S-allyl-cysteine sulfoxide (alliin).	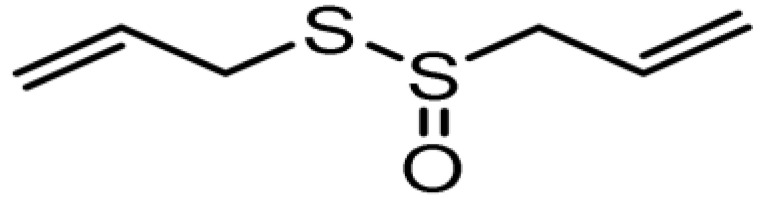 Allicin (C_6_H_10_OS_2_) [S-(2-propenyl-2-propene-1-sulfinothioate]*The most biologically active sulfur-containing compound	Inhibit platelet aggregation. Reduce blood pressure. Reduce total cholesterol. Reduce low-density lipoprotein cholesterol. Improve vasodilatation.	[248,249,250]
2nd	*Persea americana* Mill.	Polyphenols, carotenoids, tocopherols, and sterols.	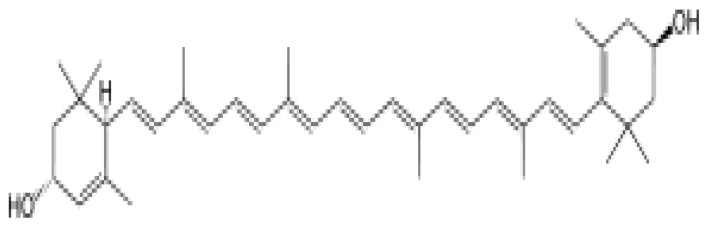 Lutein (C_40_H_56_O_2_) *The predominant carotenoid	Reduce blood pressure. Reduce triglycerides level. Reduce total cholesterol. Reduce low-density lipoprotein cholesterol. Increase high-density lipoprotein cholesterol. Reduce plasma glucose level.	[251,252]
3rd	*Moringa oleifera* Lam.	Leaf (phenolic compounds), seeds (glucosinolates), stems and flowers (alkaloids, phenolic compounds and glucosinolates).	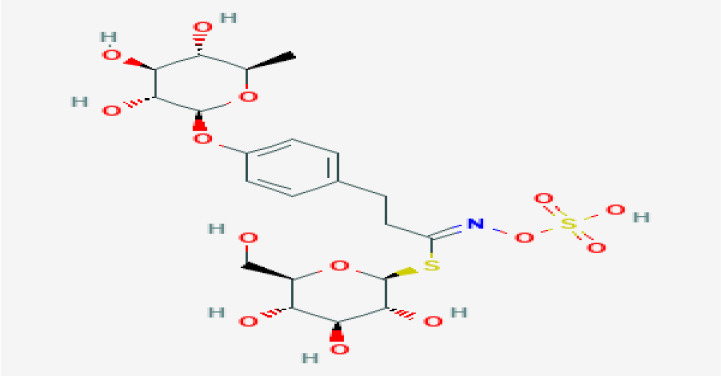 Glucomoringin (C_21_H_31_NO_14_S_2_) 4-[(α-L-rhamnosyloxy)-benzyl]-glucosinolate *The most predominant glucosinolate in the leaves, stem, flowers, pods, and seeds	Reduce blood pressure. Reduce triglycerides level. Reduce total cholesterol. Reduce low-density lipoproteincholesterol. Increase high-density lipoproteincholesterol. Reduce blood glucose level.	[253,254,255,256,257,258]
4th	*Mangifera indica* L.	Gallotannins, gallic acid and its derivatives, mangiferin, flavonoids, catechin and phenolic acids.	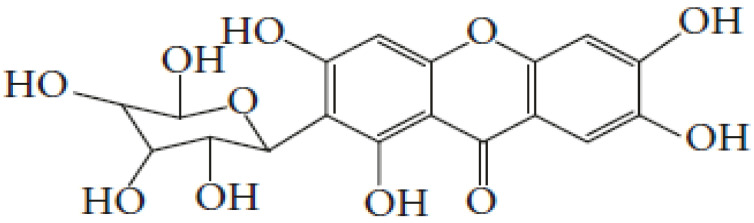 Mangiferin (C_19_H_18_O_11_) (2-β-D-glucopyranosyl-1, 3, 6, 7-tetrahydroxyxanthone) *Major component in mango stem bark extract	Reduce serum total cholesterol level and glucose absorption.	[4,219,259,260,261,262]
5th	*Allium cepa* L.	Flavonoids (particularly flavonols),frutooligosaccharides and sulfur compounds. Characterised for its flavonol, quercetin, and quercetin derivatives.	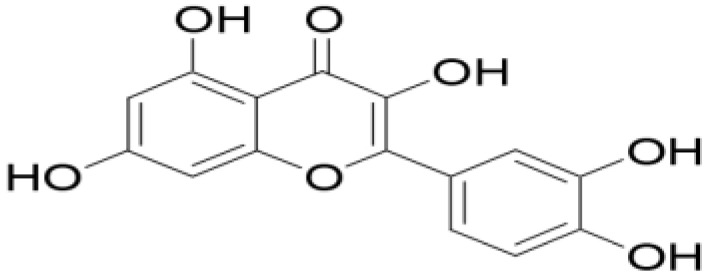 Quercetin (C_15_H_10_O_7_)	Inhibit platelet aggregation. Reduce serum triglycerides and cholesterol levels. Alleviate hyperglycemia.	[4,263,264,265,266,267]

## Data Availability

Details of the data used in the present study are already provided herein.
